# Organ Preservation and Survival by Clinical Response Grade in Patients With Rectal Cancer Treated With Total Neoadjuvant Therapy: A Secondary Analysis of the OPRA Randomized Clinical Trial

**DOI:** 10.1001/jamanetworkopen.2023.50903

**Published:** 2024-01-09

**Authors:** Hannah M. Thompson, Dana M. Omer, Sabrina Lin, Jin K. Kim, Jonathan B. Yuval, Floris S. Verheij, Li-Xuan Qin, Marc J. Gollub, Abraham Jing-Ching Wu, Meghan Lee, Sujata Patil, Aram F. Hezel, Jorge E. Marcet, Peter A. Cataldo, Blase N. Polite, Daniel O. Herzig, David Liska, Samuel Oommen, Charles M. Friel, Charles A. Ternent, Andrew L. Coveler, Steven R. Hunt, Julio Garcia-Aguilar

**Affiliations:** 1 Colorectal Service, Department of Surgery, Memorial Sloan Kettering Cancer Center, New York, New York; 2 Department of Epidemiology & Biostatistics, Memorial Sloan Kettering Cancer Center, New York, New York; 3 Department of Radiology, Memorial Sloan Kettering Cancer Center, New York, New York; 4 Department of Radiation Oncology, Memorial Sloan Kettering Cancer Center, New York, New York; 5 Department of Quantitative Sciences, Cleveland Clinic, Cleveland, Ohio; 6 James P. Wilmot Cancer Center, University of Rochester, Rochester, New York; 7 Department of Surgery, University of South Florida, Tampa; 8 Department of Surgery, University of Vermont, Burlington; 9 Department of Surgery, University of Chicago, Chicago, Illinois; 10 Department of Surgery, Oregon Health & Science University, Portland; 11 Department of Surgery, Cleveland Clinic, Cleveland, Ohio; 12 Department of Surgery, John Muir Health, Walnut Creek, California; 13 Department of Surgery, University Hospital, University of Virginia Health System, Charlottesville; 14 Department of Surgery, Creighton University Medical Center, Omaha, Nebraska; 15 Department of Medicine, University of Washington, Seattle; 16 Department of Surgery, Washington University, St Louis, Missouri

## Abstract

**Importance:**

Assessing clinical tumor response following completion of total neoadjuvant therapy (TNT) in patients with locally advanced rectal cancer is paramount to select patients for watch-and-wait treatment.

**Objective:**

To assess organ preservation (OP) and oncologic outcomes according to clinical tumor response grade.

**Design, Setting, and Participants:**

This was secondary analysis of the Organ Preservation in Patients with Rectal Adenocarcinoma trial, a phase 2, nonblinded, multicenter, randomized clinical trial. Randomization occurred between April 2014 and March 2020. Eligible participants included patients with stage II or III rectal adenocarcinoma. Data analysis occurred from March 2022 to July 2023.

**Intervention:**

Patients were randomized to induction chemotherapy followed by chemoradiation or chemoradiation followed by consolidation chemotherapy. Tumor response was assessed 8 (±4) weeks after TNT by digital rectal examination and endoscopy and categorized by clinical tumor response grade. A 3-tier grading schema that stratifies clinical tumor response into clinical complete response (CCR), near complete response (NCR), and incomplete clinical response (ICR) was devised to maximize patient eligibility for OP.

**Main Outcomes and Measures:**

OP and survival rates by clinical tumor response grade were analyzed using the Kaplan-Meier method and log-rank test.

**Results:**

There were 304 eligible patients, including 125 patients with a CCR (median [IQR] age, 60.6 [50.4-68.0] years; 76 male [60.8%]), 114 with an NCR (median [IQR] age, 57.6 [49.1-67.9] years; 80 male [70.2%]), and 65 with an ICR (median [IQR] age, 55.5 [47.7-64.2] years; 41 male [63.1%]) based on endoscopic imaging. Age, sex, tumor distance from the anal verge, pathological tumor classification, and clinical nodal classification were similar among the clinical tumor response grades. Median (IQR) follow-up for patients with OP was 4.09 (2.99-4.93) years. The 3-year probability of OP was 77% (95% CI, 70%-85%) for patients with a CCR and 40% (95% CI, 32%-51%) for patients with an NCR (*P* < .001). Clinical tumor response grade was associated with disease-free survival, local recurrence-free survival, distant metastasis-free survival, and overall survival.

**Conclusions and Relevance:**

In this secondary analysis of a randomized clinical trial, most patients with a CCR after TNT achieved OP, with few developing tumor regrowth. Although the probability of tumor regrowth was higher for patients with an NCR compared with patients with a CCR, a significant proportion of patients achieved OP. These findings suggest the 3-tier grading schema can be used to estimate recurrence and survival outcomes in patients with locally advanced rectal cancer who receive TNT.

**Trial Registration:**

ClinicalTrials.gov Identifier: NCT02008656

## Key Points

**Question:**

Can a 3-tier grading schema be used to estimate clinical tumor response after total neoadjuvant therapy (TNT) for patients with locally advanced rectal cancer?

**Findings:**

This secondary analysis of the Organ Preservation in Patients with Rectal Adenocarcinoma trial (a phase 2, randomized clinical trial) assessed clinical tumor response grade after TNT among 304 patients using 3 grades and found clinical tumor response grade was significantly associated with survival and organ preservation.

**Meaning:**

These findings suggest that the 3-tier grading schema can be used to counsel patients regarding their expected organ preservation and survival outcomes on the basis of their individual clinical tumor response grade.

## Teaser

This secondary analysis of a randomized clinical trial assesses organ preservation and survival outcomes for patients with rectal cancer according to a 3-tier clinical tumor response grading schema.

## Introduction

The watch-and-wait (WW) strategy is a newer treatment option for patients with locally advanced rectal cancer with a clinical complete response (CCR) to neoadjuvant therapy. WW is based on the assumption that patients with a CCR do not have viable cancer cells left in the bowel wall or regional lymph nodes and allows patients to forgo the potential morbidity of a surgical resection.^1,2^ Previous work has shown that WW for patients with a CCR is clinically feasible and oncologically safe.^3,4,5,6,7,8^ However, a CCR does not always represent complete tumor eradication, and some patients offered WW at the end of neoadjuvant therapy develop tumor regrowth during surveillance. Although most cases of regrowth are salvageable with total mesorectal excision (TME), concerns persist about the potential for tumor spread from clinically occult cancer cells during this surveillance period.^3,4,8,9^

Clinical response is initially assessed at a fixed interval after neoadjuvant therapy using digital rectal examination, endoscopy, and magnetic resonance imaging (MRI). However, the time that it takes to achieve a complete response can vary. Although tumor and treatment characteristics seem to be associated with differences in tumor response, there is evidence suggesting delayed assessment after neoadjuvant therapy increases the rate of response.^10,11,12^ The rate of response also depends on the criteria used to define a CCR. Assessing response early and applying strict response criteria could potentially deprive patients with a substantial response, but not a complete response, of the potential benefit of organ preservation (OP).^13,14,15,16^ Thus, the potential benefit of maximizing response by offering WW to patients with less than a CCR must be balanced against the theoretical risk of tumor progression in patients who delay surgery and never achieve a sustained complete response.

To maximize the possibility of OP, the Organ Preservation in Patients with Rectal Adenocarcinoma (OPRA) trial—a multicenter, nonblinded, phase 2 randomized clinical trial—introduced a 3-tier grading schema to clinically categorize tumor response.^17^ We have previously reported OP and oncological outcomes for the entire cohort.^5^ Here, we discuss the rates of OP and survival based on the clinical tumor response grade.

## Methods

### Patients

This secondary analysis of the OPRA randomized clinical trial was approved by a centralized institutional review board at Memorial Sloan Kettering Cancer Center as well as the participating centers’ individual institutional review boards, and it followed the Consolidated Standards of Reporting Trials (CONSORT) reporting guideline for randomized clinical trials. The OPRA trial enrolled patients aged 18 years and older with biopsy-proven clinical stage II or III rectal adenocarcinoma. Written informed consent was obtained from eligible patients. Participants were randomized to receive total neoadjuvant therapy (TNT) in the form of either induction systemic chemotherapy followed by chemoradiation or chemoradiation followed by systemic chemotherapy. The patient selection criteria, TNT regimens, and results have previously been published.^5,17^ The trial protocol is available in Supplement 1.

### Clinical Tumor Response Assessment

Clinical tumor response was assessed at 8 (±4) weeks following TNT by the treating surgeon using digital rectal examination and flexible endoscopy. Surgeons completed a tumor assessment form (TAF) with predefined clinical and endoscopic features. Clinical tumor response grade was determined on the basis of a 3-tier grading schema to classify patients into the following groups: CCR, near complete response (NCR), and incomplete clinical response (ICR) (eFigure 1 in Supplement 2). The features corresponding to the lowest clinical tumor response tier defined the patient’s overall clinical tumor response grade. MRI was also performed at restaging either before or after flexible sigmoidoscopy. The recommendation to proceed with surgery or WW was made by the surgeon and documented on the TAF. The MRI results may have influenced surgeons’ recommendations, but the contribution of the MRI to the decision process was not recorded on the TAF. The protocol indicated that in cases of response discrepancy between the endoscopic and MRI images, priority should be given to endoscopic imaging, and the decision should be based on the endoscopic imaging.^5^ The final decision was made by the individual investigators, and the protocol did not require case discussion at a tumor board or documentation of the discussion.

### Management After TNT

After completing TNT, patients with an ICR were recommended to undergo TME. Patients with an NCR or CCR were offered the option of entering the WW protocol. Patients under WW were followed at frequent intervals using digital rectal examination, flexible sigmoidoscopy, MRI, carcinoembryonic antigen testing, and computed tomography as described previously.^17^ Those with a subsequent tumor regrowth during surveillance were recommended salvage TME. Patients who underwent TME at restaging or during WW were followed according to National Comprehensive Cancer Network guidelines.^18^

### Outcomes

Disease-free survival (DFS), distant-metastasis-free survival (DMFS), local-recurrence-free survival (LRFS), overall survival (OS), and OP were defined as previously described and reported by response grade based on an intention-to-treat analysis.^5^ Briefly, events for the DFS analysis included death for any cause, distant metastasis, locoregional failure, and new invasive primary colorectal cancer. OP was defined as TME-free survival with the clinical tumor response grades assessed in an intention-to-treat manner. Patients who were recommended TME and refused, underwent local excision, or had disease progression were considered to have undergone TME. Patients with an ICR were recommended TME per protocol; patients with ICR who declined surgery and/or were followed under the WW protocol were considered to have undergone TME. All survival outcomes (DFS, DMFS, LRFS, and OS) were defined to start on the restaging date; if the restaging date was not available, 1 month prior to the TME date was the proxy restaging date. The end date for each survival outcome was defined as the first occurrence of the respective survival event, and patients without events were censored at the end of their available follow-up.

### Statistical Analysis

Demographic, clinical, and treatment baseline characteristics were summarized using median (IQR) for continuous variables and frequency and percentages for categorical variables. Characteristics were compared by the 3 clinical tumor response grades using the Fisher exact test for categorical variables and the Kruskal-Wallis rank sum test for continuous variables. Kaplan-Meier curves provided point estimates, and comparisons between clinical tumor response grades were made using the log-rank test. All analyses were performed using R statistical software version 4.2.2 (R Project for Statistical Computing). *P* values were 2-sided and were considered statistically significant if <.05. Data analysis occurred from March 2022 to July 2023.

## Results

**Figure 1.**
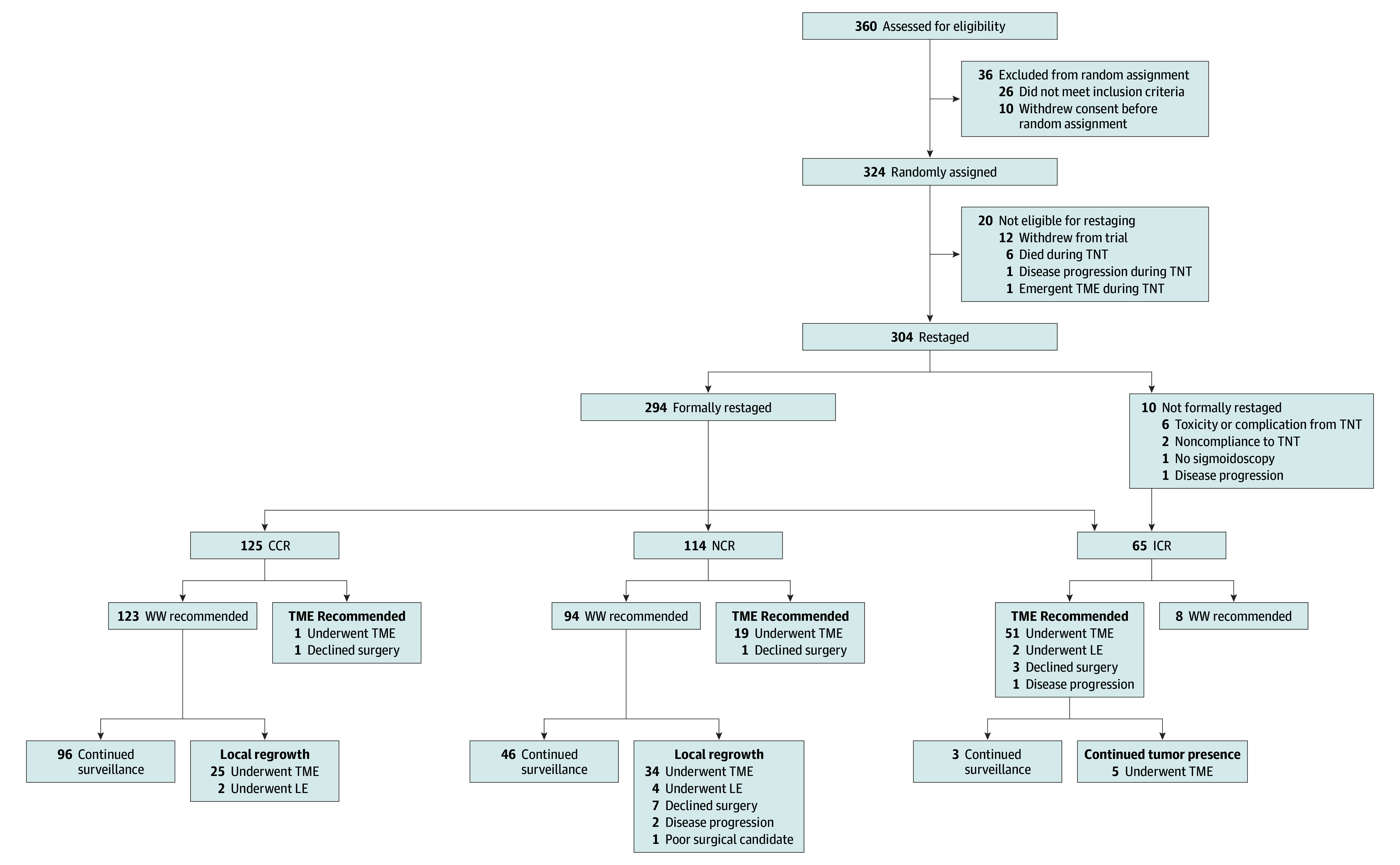
Diagram of the Clinical Tumor Response Grades Based on Intention-to-Treat CCR indicates clinical complete response; ICR, incomplete clinical response; LE, local excision; NCR, near complete response; TME, total mesorectal excision; TNT, total neoadjuvant therapy; WW, watch-and-wait.

A total of 324 patients were randomized between April 2014 and March 2020. Twenty were excluded from analysis (Figure 1).^5^ Of the 304 analyzable patients, 294 had a restaging TAF and were grouped on the basis of their clinical tumor response grade. The remaining 10 patients did not have completed restaging TAFs due to toxicities or complications during TNT (6 patients), patient noncompliance (2 patients), inability to undergo flexible sigmoidoscopy (1 patient), or concerns for progression (1 patient). These patients were all recommended to have TME and were assigned to the ICR group based on intention to treat (Figure 1).

Of the 304 patients, there were 125 patients with a CCR (41.1%; median [IQR] age, 60.6 [50.4-68.0] years; 76 male [60.8%]), 114 patients with an NCR (37.5%; median [IQR] age, 57.6 [49.1-67.9] years; 80 male [70.2%]), and 65 patients with an ICR (21.4%; median [IQR] age, 55.5 [47.7-64.2] years; 41 male [63.1%]) (Table 1). We found no differences between the clinical tumor response grades based on age, sex, or tumor distance from the anal verge. There were more patients whose lymph node status was positive in the ICR group (51 of 65 patients [78.5%]) compared with the NCR (86 of 114 patients [75.4%]) and the CCR groups (80 of 125 patients [64.0%]), but the differences were not statistically significant. Two patients in the ICR group did not receive chemotherapy, and 1 patient did not receive chemoradiation. The proportion of patients receiving all their intended cycles of FOLFOX (fluorouracil, oxaliplatin, and leucovorin) or CAPEOX (capecitabine and oxaliplatin) was lower in the ICR group (50 of 65 patients [76.9%]) compared with the NCR group (112 of 114 patients [98.2%]) or the CCR group (114 of 125 patients [91.2%]). Time from the end of TNT to restaging did not vary between groups.

**Table 1.** Baseline Demographics and TNT Results Based on Clinical Tumor Response

Characteristic	Clinical complete response (n = 125)	Near complete response (n = 114)	Incomplete clinical response (n = 65)
Age, median (IQR), y	60.6 (50.4-68.0)	57.6 (49.1-67.9)	55.5 (47.7-64.2)
Sex			
Female	49 (39.2)	34 (29.8)	24 (36.9)
Male	76 (60.8)	80 (70.2)	41 (63.1)
Tumor distance from anal verge, median (IQR), cm^a^	4.5 (3.3-7.0)	4.0 (3.0-5.9)	4.5 (3.0-6.3)
cT classification			
cT1-cT2	16 (12.8)	11 (9.6)	5 (7.7)
cT3	95 (76.0)	87 (76.3)	51 (78.5)
cT4	14 (11.2)	16 (14.0)	9 (13.8)
cN classification			
cN-negative	45 (36.0)	28 (24.6)	14 (21.5)
cN-positive	80 (64.0)	86 (75.4)	51 (78.5)
Treatment group			
Induction chemotherapy followed by chemoradiation	55 (44.0)	60 (52.6)	31 (47.7)
Chemoradiation followed by consolidation chemotherapy	70 (56.0)	54 (47.4)	34 (52.3)
Chemotherapy received			
Fluorouracil, oxaliplatin and leucovorin	98 (78.4)	86 (75.4)	42 (64.6)
Capecitabine and oxaliplatin	21 (16.8)	26 (22.8)	17 (26.2)
Both	6 (4.8)	2 (1.8)	4 (6.2)
None	0	0	2 (3.1)
Received intended chemotherapy cycles	114 (91.2)	112 (98.2)	50 (76.9)
Radiosensitizing therapy			
Capecitabine	105 (84)	96 (84.2)	54 (83.1)
Fluorouracil	19 (15.2)	18 (15.8)	8 (12.3)
None	1 (0.8)	0	3 (4.7)
Radiation dose, median (IQR), cGy^b^	5400 (5040-5600)	5400 (5040-5400)	5400 (5000-5400)
Time from end of TNT to clinical response assessment, median (IQR), wk^c^	7.6 (5.9-9.0)	8.0 (6.0-9.7)	7.7 (5.7-9.5)
Time from start of TNT to clinical response assessment, median (IQR), wk	34.3 (32.3-36.4)	35.0 (32.2-36.8)	34.7 (32.0-37.5)

Abbreviations: cN, clinical node; cT, clinical tumor; TNT, total neoadjuvant therapy.

^a^ One patient did not have distance from anal verge provided.

^b^ One patient did not receive radiation.

^c^ Weeks from the last date of TNT; one patient was unknown.

The surgical and pathological characteristics of the 144 patients requiring surgery are presented in Table 2. Of the 125 patients with a CCR, 28 (22.4%) underwent surgery, whereas 58 of the 114 patients with an NCR (50.9%) and 58 of 65 patients with an ICR (89.2%) underwent surgery. There were no significant differences in the proportion of patients who underwent TME or local excision between the clinical tumor response grades. More patients in the ICR group had node-positive (ypN+) disease (16 of 58 patients [27.6%]) compared with the CCR group (4 of 28 patients [14.3%]) and the NCR group (6 of 58 participants [10.3%]), but the difference did not reach statistical significance.

**Table 2.** Surgical Characteristics Based on Clinical Tumor Response

Characteristic	Patients who underwent surgery, No. (%) (N =144)			
	Clinical complete response (n = 28)	Near complete response (n = 58)	Incomplete clinical response (n = 58)	*P* value^a^
Resection type				
Total mesorectal excision	26 (92.9)	53 (91.4)	56 (96.6)	.50
Local excision	2 (7.1)	5 (8.6)	2 (3.4)	
Pathological tumor classification (ypT)				
T0	3 (10.7)	5 (8.6)	4 (6.9)	.80
Tis	2 (7.1)	3 (5.2)	1 (1.7)	
T1	2 (7.1)	5 (8.6)	4 (6.9)	
T2	8 (28.6)	22 (37.9)	17 (29.3)	
T3	11 (39.3)	20 (34.5)	30 (51.7)	
T4	2 (7.1)	2 (3.4)	2 (3.4)	
Missing	0	1 (1.7)	0	
Pathological nodal classification (ypN)				
N-negative	22 (78.6)	47 (81)	40 (69.0)	.13
N-positive	4 (14.3)	6 (10.3)	16 (27.6)	
N-absent^b^	2 (7.1)	5 (8.6)	2 (3.4)	

^a^ Significance evaluated with the Fisher exact test.

^b^ Absent lymph nodes from the local excision surgical specimen.

Of the 125 patients with a CCR, 2 were offered upfront TME; 1 patient was offered upfront TME for a rectal stricture that precluded effective surveillance with a subsequent pathologic complete response (PCR), and the other patient was offered upfront TME based on the site’s tumor board recommendations. Of note, the second patient declined resection and never developed tumor regrowth. Both patients remained without evidence of disease during their follow-up periods. Of the 125 patients with CCR, 123 (98.4%) were offered WW, and 27 of those offered WW (22.0%) developed local regrowth. A total of 25 patients in the CCR group underwent salvage TME, and 2 had salvage local excision. The survival events for those 27 patients are outlined in the eTable in Supplement 2. Of the remaining 96 patients with a sustained CCR until the end of follow-up, 5 (5.2%) developed distant metastases, with 4 subsequently dying from disease. One patient died from unrelated causes.

Of the 114 patients with an NCR at restaging, 20 (17.5%) were recommended surgery; 19 underwent TME and 1 underwent local excision. Three of the 19 patients who underwent TME had a PCR. Of the 114 patients with an NCR, 94 (82.5%) were offered WW, and 48 of those offered WW (51.1%) had local regrowth; of those patients, 34 underwent TME, 4 underwent local excision, 8 refused TME, and 2 were not considered candidates for TME due to disease progression. Two of the 34 patients who underwent TME were found to have a PCR. The eTable in Supplement 2 describes the survival events for these patients. Forty-six patients with an NCR continued WW during the follow-up and preserved their rectum; 6 of those patients (13.0%) developed distant metastases with 2 subsequent deaths, and 2 patients died without any reported local or systemic disease.

Finally, of the 65 patients in the ICR group, 57 (87.7%) were recommended TME at restaging. Fifty-one patients underwent TME, 2 underwent a local excision, 3 refused surgical resection, and 1 patient was not considered a surgical candidate due to disease progression. Of the 51 patients who underwent TME, 4 had a PCR. Survival events for patients with an ICR are presented in the eTable in Supplement 2. Although the protocol recommended TME surgery for all patients with ICR at restaging, 8 of 65 patients in the ICR group (12.3%) were followed under WW for different reasons (borderline response, rectovaginal fistula, or discrepancy between clinical observations and pathology on a tissue biopsy). Of these 8 patients with an ICR, 5 eventually required TME for tumor persistence or regrowth. The remaining 3 patients were managed nonoperatively because they demonstrated improved clinical response over time. Of note, 2 of these 3 patients developed lung metastases, and 1 subsequently died. For the intention-to-treat analysis, these patients were considered to have undergone TME at the time of restaging.

**Figure 2.**
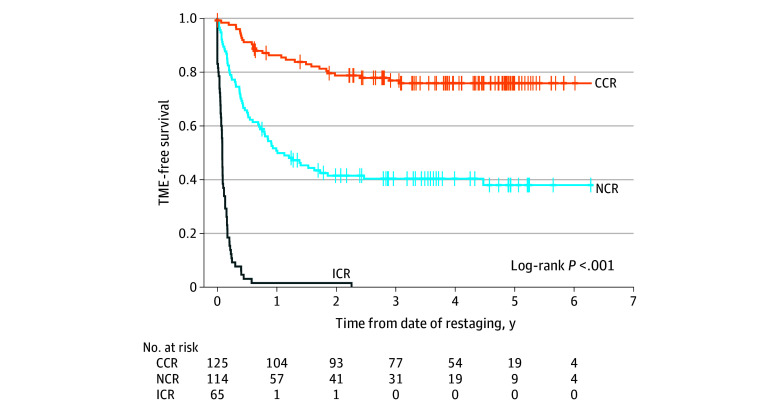
Organ Preservation According to Clinical Tumor Response Grade by Intention-to-Treat CCR indicates clinical complete response; ICR, incomplete clinical response; NCR, near complete response; TME, total mesorectal excision.

The rate of OP at 3 years was 47% (95% CI, 41%-53%) for the entire cohort (eFigure 2 in Supplement 2), 77% (95% CI, 70%-85%) for the CCR grade, and 40% (95% CI, 32%-51%) for the NCR grade (*P* < .001) (Figure 2). The median (95% CI) time to TME from restaging was 1.01 (0.75-4.48) years for the NCR group and was not reached for the CCR group. The risk of local regrowth at 2 years was 20% (95% CI, 13%-27%) for the CCR group and 49% (95% CI, 40%-61%) for the NCR group (eFigure 3 in Supplement 2).

**Figure 3.**
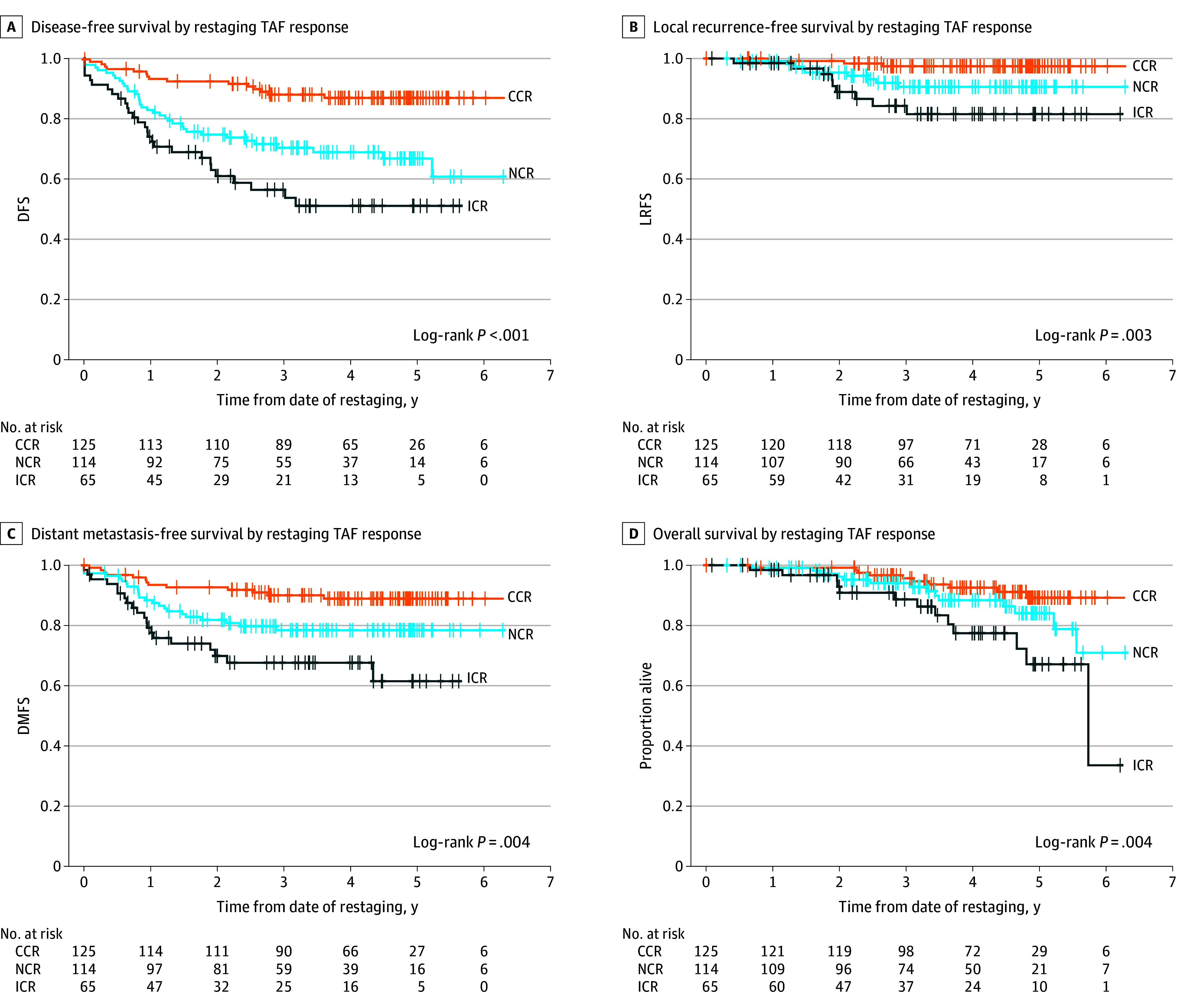
Disease-Free Survival (DFS), Local Recurrence-Free Survival (LRFS), Distant Metastasis-Free Survival (DMFS), and Overall Survival (OS) The figure shows DFS (A), LRFS (B), DMFS (C), and OS (D) according to clinical tumor response grade by intention to treat. CCR indicates clinical complete response; ICR incomplete clinical response; NCR, near complete response; TAF, tumor assessment form.

DFS at 3 years for the entire cohort was 74% (95% CI, 69%-79%) (eFigure 2 in Supplement 2). There was a significant difference in DFS (*P* < .001) between the clinical tumor response grades (CCR group, 88% [95%CI, 82%-94%]; NCR group, 69% [95% CI, 61%-78%]; ICR group, 56% [95% CI, 44%-71%) (Figure 3). We also found differences for LRFS, DMFS, and OS between the clinical tumor response grades (Figure 3).

## Discussion

In this secondary analysis of a randomized clinical trial, we found that clinical tumor response grade at restaging was associated with OP and survival in patients with locally advanced rectal cancer treated with neoadjuvant therapy. Although many patients with an NCR who were offered WW after neoadjuvant therapy ultimately developed tumor regrowth and required TME, a significant portion achieved OP. In addition, we observed a direct association of clinical tumor response grade with tumor recurrence and patients’ survival, suggesting that clinical tumor response at restaging has prognostic implications.

The clinical criteria used to select patients for WW have historically been restrictive to avoid undertreating tumors potentially harboring viable cancer cells after neoadjuvant therapy.^15,19^ Additionally, the optimal timing of surgery after neoadjuvant therapy has been controversial given the concern (although largely unseen) for increased technical surgical difficulty and perioperative morbidity with longer intervals.^10,20,21,22,23,24,25,26^ However, tumor response can take months^10,26,27^ (ie, longer than the typical 6 to 8 weeks used as a standard interval between completion of TNT and TME surgery). Thus, an early assessment of clinical response using strict response criteria can lead to unnecessary TME in patients who may have otherwise developed a CCR.^13,26,28^ To maximize the opportunity for OP, the OPRA trial introduced the concept of an NCR to define a group of patients without gross residual tumor but with clinical, endoscopic, and MRI characteristics not meeting the accepted criteria of a CCR.^17^ In our study, most patients achieved a CCR or NCR when clinical tumor response was assessed at an median of 7 weeks from completion of TNT and were initially offered WW. Consistent with the retrospective literature,^3,8,29,30,31^ most patients with a CCR never developed local regrowth and achieved sustained OP. The rate of local regrowth for the CCR group was comparable with previous retrospective case series^3,32^ reporting only on CCR patients. Of the patients with an NCR, almost one-half developed local regrowth by 5 years and ultimately required TME. However, this finding means that approximately one-half of patients with an NCR avoided surgery. These results are consistent with a recent retrospective study^13^ that reported a 47% OP rate in patients with rectal cancer who were given additional time to respond after neoadjuvant therapy.

A concern with OP strategies is the potential increased risk for distant metastases in patients with an initial CCR or NCR who are offered WW and subsequently develop a local regrowth.^8,33^ In our study, the rate of distant metastasis in patients with a sustained CCR who were offered WW was lower compared with patients who were offered WW and developed local regrowth. However, this finding does not imply that a metastasis necessarily originates from tumor regrowth. Our series also showed that the rate of distant metastasis among patients with a CCR or NCR who developed tumor regrowth was similar to patients recommended TME immediately after restaging. These results could suggest that the risk of metastasis for patients without a sustained complete response is similar independent of immediate surgery for incomplete response or delayed surgery for tumor regrowth. Furthermore, these results emphasize the favorable outcomes of patients with a sustained complete response compared with patients with residual tumor at the primary site. It is possible to speculate that lack of response, manifested as tumor persistence or regrowth, represents aggressive biology and a greater risk of metastasis. Interestingly, our data also showed that the rate of distant metastases after sustained clinical tumor response in patients who achieved OP and did not experience tumor regrowth was higher in the NCR group than in the CCR group. There is not an obvious explanation for this finding. We cannot exclude the possibility that some of the NCR patients with an apparent sustained CCR and distant metastasis may also have had occult but viable cancer cells at the primary tumor site that could manifest with longer follow-up. However, the proportions of distant metastases in both groups, 5.2% in the CCR group and 13.0% in the NCR group, are in the range of distant metastases seen in patients with rectal cancer with PCR after chemoradiation and TME.^1,34,35^

There is ample evidence suggesting that pathologic tumor response to preoperative chemoradiation has prognostic value in patients with rectal cancer.^1,34,35,36^ In pathologic specimens from patients with stage II and III rectal cancers treated with chemoradiation, Park et al^34^ reported that PCR (ypT0N0) was associated with excellent outcomes with few distant metastases. In addition, an intermediate pathologic response (ypT1N0-ypT2N0) had improved oncologic outcomes compared with a poor response (ypT3, ypT4, or N+). Similarly, Fokas et al^35^ showed stratification of pathologic response with a simplified 3-tier tumor regression schema, which was associated with outcomes in patients with locally advanced rectal cancer treated with neoadjuvant therapy and TME surgery.^36^ The results of the OPRA trial mirror these pathologic studies and suggest that clinical stratification of tumor response after TNT has similar prognostic implications; this also supports the idea that the lack of clinical tumor response to neoadjuvant therapy reflects an inherent aggressive biology of the tumor that is manifested by a propensity to resist neoadjuvant therapy and to metastasize. Our study hypothesizes that across clinical tumor response grades, prognostication is largely independent of surgical management of the primary tumor.

### Limitations

There are some limitations to our study. First, the clinical tumor response grades were based on the surgeon’s assessment of the endoscopic and digital rectal examinations at restaging. The contribution of the MRI studies was not fully ascertained in this study. However, the study protocol recommended giving endoscopic assessment precedence over MRI imaging in tumor response decision making.^5^ Further work is needed to improve the diagnostic accuracy overall. Merging endoscopic imaging and MRI may provide insight into the optimal identification of patients most likely to achieve long-term OP. Additionally, other tools, such as artificial intelligence or narrow band imaging, may be coupled with endoscopy and provide additional methods for improving the current accuracy. Second, although the study protocol standardized the definitions for clinical tumor response into 3 grades, the interpretation of the images and assignment to a response grade was subjective.^37^ Although central review of the images may have provided different results, the approach used in the OPRA trial more closely mirrors the image interpretation and decision-making process that would be implemented in the community. Third, the time of assessment of clinical tumor response after TNT was chosen somewhat arbitrarily on the basis of clinical experience. It is possible that a different timing for the assessment of clinical tumor response would have provided different results.

## Conclusions

In this secondary analysis of the OPRA trial, there was a higher probability of tumor regrowth for patients with an NCR compared with a CCR, but a significant proportion of patients achieved a sustained CCR and OP. The 3-tier grading schema has prognostic implications for recurrence and survival in patients with locally advanced rectal cancer who received TNT and should be used to counsel patients regarding their expected outcomes.

## Notes

Supplement 1.
Trial Protocol

Supplement 2. **eFigure 1.** Three-Tier Schema for Grading Tumor Response **eTable.** Local and Distant Metastases for TME at Restaging Versus TME After Local Regrowth in Relation to Clinical Tumor Response **eFigure 2.** Rates of Organ Preservation and Disease-Free Survival for the Full Cohort **eFigure 3.** Rates of Local Regrowth

Supplement 3.
Data Sharing Statement
