# Error in Byline

**DOI:** 10.1001/jamanetworkopen.2024.2456

**Published:** 2024-02-21

In the Original Investigation titled “Organ Preservation and Survival by Clinical Response Grade in Patients With Rectal Cancer Treated With Total Neoadjuvant Therapy: A Secondary Analysis of the OPRA Randomized Clinical Trial,”^1^ published January 9, 2024, there was an error in an author’s name in the byline, which should have appeared as Floris S. Verheij, BSc. This article has been corrected.^1^
