# Error in Results

**DOI:** 10.1001/jamanetworkopen.2025.6079

**Published:** 2025-03-19

In the Original Investigation titled “Organ Preservation and Survival by Clinical Response Grade in Patients With Rectal Cancer Treated With Total Neoadjuvant Therapy: A Secondary Analysis of the OPRA Randomized Clinical Trial,”^1^ published January 9, 2024, there was an error in the penultimate paragraph of the Results, in which the 95% CI for risk of local regrowth at 2 years among patients with a clinical complete response was incorrectly reported as 70% to 85% rather than 13% to 27%. This article has been corrected.^1^
