# Supplementary material for: Organ Preservation and Survival by Clinical Response Grade in Patients With Rectal Cancer Treated With Total Neoadjuvant Therapy: A Secondary Analysis of the OPRA Randomized Clinical Trial
Source: JAMA Netw Open. 2024 Jan 9;7(1):e2350903. doi: 10.1001/jamanetworkopen.2023.50903 (PMC10777257; doi:10.1001/jamanetworkopen.2023.50903)
Supplement: Supplement 1. — Trial Protocol [file jamanetwopen-e2350903-s001.pdf]

MSK PROTOCOL COVER SHEET

A phase II multicenter randomized trial evaluating 3-year disease-free survival in patients with locally advanced rectal cancer treated with chemoradiation plus induction or consolidation chemotherapy, and total mesorectal excision or non-operative management.

Principal Investigator/Department: Julio Garcia Aguilar, MD/Surgery-Colorectal.

Table of Contents

1.0 PROTOCOL SUMMARY AND/OR SCHEMA 3
2.0 OBJECTIVES AND SCIENTIFIC AIMS 5
3.0 BACKGROUND AND RATIONALE 5
4.0 OVERVIEW OF STUDY DESIGN/INTERVENTION 12
4.1 Design 13
4.2 Intervention 13
5.0 THERAPEUTIC/DIAGNOSTIC AGENTS 14
6.0 CRITERIA FOR SUBJECT ELIGIBILITY 16
6.1 Subject Inclusion Criteria 16
6.2 Subject Exclusion Criteria 17
7.0 RECRUITMENT PLAN 18
8.0 PRETREATMENT EVALUATION 19
9.0 TREATMENT/INTERVENTION PLAN 20
10.0 EVALUATION DURING TREATMENT/INTERVENTION 34
11.0 TOXICITIES/SIDE EFFECTS 36
12.0 CRITERIA FOR THERAPEUTIC RESPONSE/OUTCOME ASSESSMENT 44
13.0 CRITERIA FOR REMOVAL FROM STUDY 45
14.0 BIOSTATISTICS 46
15.0 RESEARCH PARTICIPANT REGISTRATION AND RANDOMIZATION PROCEDURES 50
15.1 Research Participant Registration 50
15.2 Randomization 51
16.0 DATA MANAGEMENT ISSUES 51
16.1 Quality Assurance 54
16.2 Data and Safety Monitoring 54
16.3 Regulatory Documentation 60
16.4 Noncompliance 62
17.0 PROTECTION OF HUMAN SUBJECTS 57
17.1 Privacy 58
17.2 Serious Adverse Event (SAE) Reporting 59
17.3 Serious Adverse Event (SAE) Reporting for Participating Sites .... **Error! Bookmark not defined.**
18.0 INFORMED CONSENT PROCEDURES 60
18.1 For participating sites 60

19.0 REFERENCES 60
20.0 APPENDICES 66

1.0 PROTOCOL SUMMARY AND/OR SCHEMA

1.1 Acronyms

Following are select acronyms related to treatment and assessments specific to this study.

- ACT: Adjuvant chemotherapy
- APR: Abdominoperineal resection
- CAA: Coloanal anastomosis
- cCR: Clinical Complete Response
- CNCT: Consolidation neoadjuvant chemotherapy
- CRT: Chemoradiation therapy
- DRE: Digital rectal exam
- DM: Distant metastasis
- INCT: Induction neoadjuvant chemotherapy
- LARC: Locally advanced rectal cancer
- NCT: Neoadjuvant chemotherapy
- NOM: Non-operative management
- pCR: Pathologic Complete Response
- TME: Total mesorectal excision
- TNT: Total neoadjuvant therapy

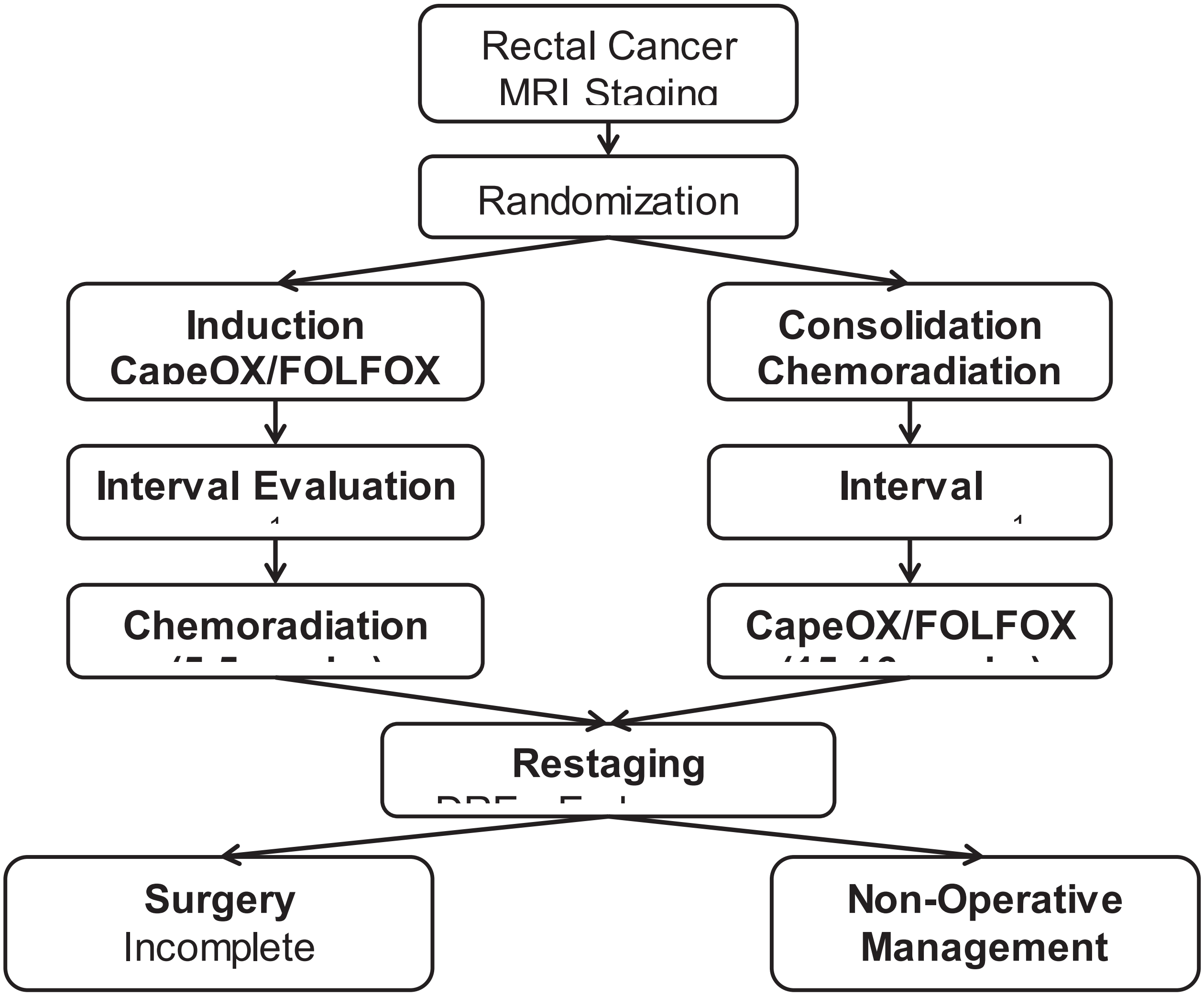

[1] Patients with tumor progression at the interval evaluation will be treated according to standard practice as per treating institution guidelines.

[2] Rectal MRI at the interval evaluation is required for MSKCC patients only. MRI at the interval evaluation is recommended but not required for participating sites. Sites are encouraged to perform DW-MRI during the interval evaluation, but it is not required. Sites that elect to perform DW-MRI are asked to submit image results to MSKCC. See section 9.5 for instructions.

This is a multicenter phase II study investigating the efficacy of total neoadjuvant therapy (TNT) and selective non-operative management (NOM) in patients with locally advanced rectal cancer (LARC). Patients will be staged at baseline using MRI pelvis for rectal cancer (hereafter MRI Rectum). Patients with clinical Stage II (T3-4, N-) or Stage III (any T, N+) MRI-staged rectal cancer and would be considered to require a complete total mesorectal excision (TME) at baseline will be randomized to one of two arms. Both arms will receive 5-FU or capecitabine-based CRT. Arm 1 will receive FOLFOX/CapeOX before CRT (induction neoadjuvant chemotherapy or INCT) and Arm 2 will receive FOLFOX/CapeOX after CRT (consolidation neoadjuvant chemotherapy or CNCT). An interval evaluation will be conducted

after completion of INCT in Arm 1, and after completion of CRT in Arm 2. Patients with tumor progression at the interval evaluation will be treated according to standard practice per institutional guidelines. Those with stable disease or evidence of response will complete neoadjuvant therapy. Patients in both arms will be re-staged after completion of all neoadjuvant therapy. Patients with incomplete response will undergo a TME. Patients with complete or near complete clinical responses will be treated with NOM. Criteria for determining complete, near complete, and incomplete response are outlined in Section 9.3.2. Patients in both groups (TME and NOM) will be followed according to NCCN guidelines for five years from the last date of neoadjuvant therapy. Patients in the NOM group will be evaluated using rectal MRIs and endoscopic exam (see section 10). Patients in the NOM group who undergo subsequent TME will be followed according to current NCCN guidelines. All patients will be followed based on intent-to-treat. Disease-free survival will be measured after three years.

2.0 OBJECTIVES AND SCIENTIFIC AIMS

2.1 Primary Objective

1. To evaluate 3-year disease-free survival (DFS) in patients managed with TNT and TME or NOM, compared with standard historical controls managed according to standard of care (CRT and TME followed by adjuvant chemotherapy [ACT]).

2.2. Secondary Objectives

1. To compare outcomes between patients in the two study arms, with respect to rates of organ preservation, compliance with the neoadjuvant protocol, and adverse events.

2. To measure patient-reported functional outcomes and quality of life (QoL) in patients with LARC treated with TNT and NOM, and compare them to patients treated with TNT and TME.

2.3 Correlative Studies Objectives

1. To investigate the diagnostic performance of conventional and diffusion-weighted magnetic resonance imaging (DW-MRI) in identifying patients with LARC treated with TNT, who may benefit from NOM.

2. To evaluate the feasibility of using circulating tumor DNA and miRNA profiles in plasma to monitor tumor response to TNT in rectal cancer patients treated in both protocol arms.

3. Use of genomic analysis by next generation sequencing to profile distal rectal cancer treated with neoadjuvant chemotherapy and radiation.

4. Investigation of the molecular mechanisms of tumor resistance to neoadjuvant therapy by genomic analysis of rectal cancer before and after treatment.

3.0 BACKGROUND AND RATIONALE

3.1 Current Standard Management for Locally Advanced Rectal Cancer

The treatment of patients with non-metastatic, LARC includes pre-operative CRT, TME, and post-operative ACT.[1] This intense trimodality treatment provides local tumor control in most

patients; but almost one-third of them ultimately die from distant metastasis (DM).[2] In addition, most survivors experience significant impairment in their QoL, due primarily to removal of the rectum.[3] Consequently, the current challenges in the treatment of LARC are: how to improve survival by reducing the risk of DM; and how to improve QoL in surviving patients by preserving the rectum.

3.2 Improvement in survival

3.2.1 Tumor recurrence in rectal cancer patients

Historically, local recurrence was the main problem for LARC patients. After decades of improvement in local tumor control through advances in imaging, surgical technique, radiation and chemotherapy, DM is now the most common form of tumor recurrence and the ultimate cause of death in these patients.

DM after curative intent surgery develops from micrometastases, which are clinically undetectable at the time of diagnosis. ACT aims to eradicate micrometastasis in patients who are otherwise destined to develop tumor recurrence. Evidence supporting postoperative ACT in patients with high-risk colon cancer is very solid, and the current standard of care for patients with stage III colon cancer includes 6 months of ACT starting within 8 weeks after surgery.[4] However, the sensitizing chemotherapy delivered during CRT has limited systemic effect. Therefore, similar to patients with colon cancer, patients with LARC at risk for DM are treated with postoperative ACT at systemic doses.[5] Unfortunately, one-third of these patients still develop DM.[1, 6] The addition of new chemotherapy agents such as bevacizumab or cetuximab to FOLFOX/CapeOx has failed to improve survival compared to FOLFOX/CapeOX alone.[7, 8] [9] Alternative approaches are needed.

3.2.2 Neoadjuvant chemotherapy (NCT)

Many LARC patients who are eligible for ACT never begin it (17%-28%), and few receive the planned dose without delay (37% to 52%).[6, 10, 11] This is because otherwise eligible patients develop postoperative complications, are unfit for further treatment, or plainly refuse it. Many refuse ACT because it may delay reversal of the diverting loop ileostomy, which is temporarily left in patients undergoing sphincter-saving procedures in order to reduce the risk of pelvic sepsis. Delivering chemotherapy before rather than after surgery will not only start treatment of occult micrometastasis several months earlier, but will increase treatment compliance. This will potentially enhance the efficacy of chemotherapy in preventing DM, and ultimately improve survival. Other benefits of neoadjuvant chemotherapy (NCT) include increased response of the primary tumor, early identification of non-responders, and timely removal of the diverting loop ileostomy.[12, 13]

NCT in rectal cancer patients has been delivered as induction, before CRT, or as consolidation, after CRT but before TME. Each regimen has potential advantages and disadvantages.

3.2.2.1 Induction neoadjuvant chemotherapy (INCT)

A number of European studies have investigated the efficacy of INCT in LARC patients treated with CRT, but the results are difficult to interpret due to study heterogeneity.[10, 12, 14-19] In most studies, however, 84% to 97% of patients completed INCT. Additionally, INCT did not impact compliance with CRT or the probability of an R0 resection. Reported rates of pathologic complete response (pCR) ranged from 7% to 29%. Two prospective trials have randomized patients to receive INCT, CRT, and TME, or CRT, TME and ACT. In the larger

trial, all 54 patients in the induction group started INCT and 51 (94%) completed all cycles. In contrast, only 37 of 49 (76%) patients in the adjuvant group started ACT, and only 28 (57%) received all cycles with or without dose reductions. pCR rates were 14% in the INCT group compared to 13% in the ACT group.[10] In another trial comparing INCT and CRT with CRT alone, 96% of patients completed the study per protocol. The pCR rate did not differ between study arms.[19] Unfortunately, the follow-up in most INCT studies has been too short, reporting survival outcomes at 12 or 18 months only, or not reporting them at all. Therefore, the effect of INCT on local recurrence and survival in LARC patients treated with CRT remains unknown.

The group at MSKCC led by Dr. Leonard Saltz has the largest experience in the United States with INCT in LARC. In the MSKCC 07-021 trial, 32 patients with stage II and III rectal cancer were treated with FOLFOX and Avastin. Two patients did not complete treatment due to toxicity, the rest had R0 resections, and 8 (25%) had a pCR. One patient died after surgery.[20] Recently, the group has reported on a series of 44 stage III and IV rectal cancer patients treated with INCT, CRT and TME. Forty-two (95%) completed the treatment per protocol. Some patients with stage IV disease did not undergo TME due to disease progression or patient refusal, but of the 37 who had TME 8 achieved a pCR (22%).[21] These data suggest that INCT is safe in LARC patients, and that most patients completed treatment per protocol.

3.2.2.2 Consolidation Neoadjuvant Chemotherapy (CNCT)

The Timing of Rectal Cancer Response to Chemoradiation (TIMING Trial; NIH NCT00335816, PI: Garcia-Aguilar) has investigated the effect of delivering 2, 4, or 6 cycles of CNCT (FOLFOX) after CRT in patients with LARC. The study completed accrual in 2012. The study found that adding 2, 4, or 6 cycles of FOLFOX after CRT increased the pCR rates to 25%, 30%, and 38%, respectively, compared to CRT alone (18%). Eighty percent of patients completed CNCT without interruption. CNCT did not increase the rate of adverse events or surgical complications.[22]

Habr-Gama et al also studied the effects of CNCT in rectal cancer patients treated with 5-FU-based CRT, by delivering 3 cycles of bolus 5-FU over 9 weeks after CRT. Patients with clinical complete response (cCR) at protocol completion were observed, and those without cCR had TME. Of the 29 patients included in the study, 28 (97%) completed all 3 cycles; 19 (65%) had a sustained cCR and avoided surgery for at least 12 months. However, follow-up was too short, and the possibility of delayed tumor relapse in some patients cannot be excluded.[23] These studies suggest that CNCT after CRT is well tolerated, with most patients completing treatment as scheduled. Additionally, CRT followed by CNCT results in higher pCR rates compared to CRT alone, without increasing the rate of postoperative complications. Similar to the INCT studies, however, no meaningful survival data is available.

3.2.2.3 INCT compared to CNCT

This will be the first study to perform a prospective comparison between patients treated with INCT and CNCT. INCT addresses occult micrometastasis earlier but delays the delivery of CRT, defying the principle that the interval from the start of treatment to the end of radiation should be as short as possible. On the other hand, CNCT delays treatment of micrometastasis for several weeks compared to INCT but does not delay CRT, and may increase tumor response by reducing the time from initiation of therapy to completion of radiation. By assessing tumor response and other measurable outcomes during and after treatment, we may be able to compare INCT and CNCT in terms of organ preservation,

treatment compliance and adverse events. We will also be able to determine the impact of each component of TNT on tumor response.

3.3 Reducing morbidity and preserving QoL by avoiding over-treatment

3.3.1 Morbidity associated with multimodality therapy of rectal cancer

Despite recent advances in surgical technique and perioperative care, TME remains a formidable operation associated with some mortality, significant morbidity, and long-lasting sequelae that permanently impair QoL. Mortality for TME ranges from 0.8% to 2.4%. Between 23% and 35% of patients develop perioperative complications.[1, 6, 11] The autonomic pelvic nerves are often damaged during TME; as a result, up to 39% of these patients develop urinary complications, and 45% have sexual dysfunction.[24] [25] In addition, a TME for distal LARC leads to one of two undesirable situations. If the tumor is too close to the anal verge for sphincter preservation, the only option is abdominoperineal resection of the rectum (APR) with a permanent colostomy. Unfortunately, many APR patients develop perineal wound separation that delays ACT, often indefinitely.[26] Additionally, APR patients suffer numerous stoma-related complications such as odor, leakage, peristomal irritation, and parastomal hernia.[27-29] Patients with tumors that are amenable to sphincter preservation require CAA and a diverting loop ileostomy. In a recent study of patients undergoing CAA for LARC, 72% reported frequency, urgency, soiling, and inability to defer defecation for 15 minutes.[30] These functional alterations have significant impact on QoL.[31, 32] While QoL after APR and CAA may not be as markedly different as many patients believe, those undergoing APR experience difficulties in several QoL domains relating to sexual function and body image, which influences their decision-making. [33, 34] Therefore, when given the chance to avoid a stoma, patients almost uniformly opt for a CAA despite its functional sequelae. Finally, many APR and CAA patients require further surgery for late complications such as bowel obstruction or incisional hernia.[24] Treatment alternatives to APR and CAA will improve functional outcomes and QoL for some rectal cancer patients.

3.3.2 Rectal cancer response to neoadjuvant therapy

A number of LARC patients have a pCR to CRT. The rate of pCR ranges from 9% to 44%, depending on the dose of radiation, the use of sensitizing chemotherapy, earlier tumor stage, and the interval between completion of neoadjuvant therapy and surgery. Patients with a pCR have lower rates of tumor recurrence and improved survival compared to non-pCR patients, [35] raising questions about the added value of surgery for these individuals.[12, 36-38] As most of the mortality, morbidity, and long-term sequelae from multimodality therapy are related to excision of the rectum, avoiding TME selectively in patients who obtain a sustained response to CRT will reduce over-treatment and improve QoL.

3.3. 3 Reducing morbidity from over-treatment

The elevated morbidity associated with the trimodality treatment of LARC has fueled a heated debate about whether all patients require such intensive treatment.[12, 36-38] LARC located in the upper rectum is associated with a lower risk of local recurrence, compared to tumors located in the distal rectum.[39] The PROSPECT trial, sponsored by the Alliance cancer group and opened to accrual at MSKCC, aims to reduce overall morbidity by changing ACT to NCT and eliminating CRT from the treatment regimen for cancer located in the upper rectum. Similar to the PROSPECT trial, our study aims to reduce over-treatment by delivering TNT to LARC patients and sparing TME to those with a sustained clinical

response to CRT. These patients may experience short- and long-term complications from TME, and would benefit most from NOM and organ preservation.

3.3. 4 Non-operative management (NOM) in rectal cancer patients

NOM of rectal cancer patients who respond to CRT has received increased attention in recent years, and now tops the list of research topics for colorectal cancer disease management teams around the world.[40]

The largest experience with NOM of rectal cancer comes from Habr-Gama's group. In their protocol, they assess clinical tumor response 8 weeks after CRT.[41] Patients with persistent tumor undergo TME; those with a significant clinical response undergo monthly evaluations with digital rectal exam (DRE), proctoscopy, CEA levels, and biopsy of suspicious lesions. Patients with evidence of tumor relapse are directed to surgery, while patients with a sustained cCR after one year continue surveillance every 3 months for an additional year and every 6 months thereafter. Twenty-seven percent of rectal cancer patients treated according to this protocol have a sustained cCR and are spared TME. Local relapse during follow-up developed in 10% of patients treated with NOM, but all had curative TME. A group from the Netherlands reported their experience with NOM in 21 patients with cCR as determined on clinical exam, MRI and endoscopic biopsy.[42] After a mean follow-up of 25 ± 19 months, 1 patient developed local relapse but was able to undergo curative salvage surgery. The other 20 patients are alive without disease. At MSKCC, rectal cancer patients with a cCR have been treated expectantly on a case-by-case basis, according to a NOM protocol.[43] A total of 33 patients have been treated with NOM and followed for a median of 23 months. Six patients have developed relapse, and all underwent curative salvage surgery. Three patients also developed DM.[43]

The combined experience of these series suggests that NOM may be a reasonable alternative to TME for patients with distal rectal cancer who develop a sustained cCR to neoadjuvant therapy. While some patients with an apparent clinical response later develop local relapse, the experience from the series published so far indicates that most are diagnosed early during surveillance and undergo curative salvage surgery.

While promising, these results have stirred controversy because of variability in patient selection criteria, definitions of response, and follow-up agendas. This will be the first prospective, multicenter trial in the United States to investigate the feasibility of selective NOM in rectal cancer patients with a sustained response to neoadjuvant therapy with systematic, prospective collection of response data (endoscopic and radiologic, see 9.3.2). The study is particularly well-suited to investigate selective NOM, because patients treated with TNT are more likely to have a pCR compared to patients treated with CRT alone.

3.4 Bowel, bladder, sexual function, and QoL in LARC patients treated with TNT with or without TME.

Both CRT and TME affect long-term function and QoL in LARC patients, but the impact of each treatment modality is not well understood. The effects of radiation and CRT on function and QoL have been investigated in patients treated for cervical, prostate and anal cancer (who typically do not undergo TME). However, the radiation fields for these tumors are different, and probably affect bowel function differently than in rectal cancer patients. To date, there have been no studies reporting on function or QoL in rectal cancer patients treated with CRT and NOM.

Our group has extensive experience studying functional outcomes in rectal cancer patients. In 2005 we developed the Bowel Function Instrument, which is designed to measure function after sphincter-preserving surgery.[44] Subsequently, we completed a prospective study evaluating patient-reported outcomes in 261 rectal cancer patients treated at a single institution over a 24-month period, using validated instruments.[45] We found that patients undergoing multimodality therapy for LARC experience significant changes in sexual function, bladder function, and QoL. These changes are similar in patients with and without sphincter preservation. While QoL improves over time, function appears to be permanently affected in all patients. Patients with sphincter preservation experience some permanent deterioration of bowel function.[45] These alterations are particularly prevalent in patients treated with radiation and/or CAA. Patients with poor bowel function appear to have worse QoL compared to those with good bowel function. This may explain why patients with LARC who undergo CAA have significantly lower QoL, compared to patients who undergo LAR or APR.

Studies on decision-making in rectal cancer therapy suggest that patients are willing to accept a slightly higher local recurrence rate in order to avoid a colostomy and/or poor function.[46, 47] Unfortunately, there is no data on the function and/or QoL in LARC patients treated with NOM compared to TME. It is likely that patients and healthcare providers minimize the functional changes associated with CRT and NOM while focusing on functional impairment after surgical management. Therefore the capture of data on functional outcomes and QoL is paramount. It will provide an accurate portrayal of the differences in functional sequelae associated with both options, leading to truly informed patient decision-making. This will be the first study to investigate function and QoL in LARC patients treated with NOM or TME after TNT.

3.5 Identifying patients who will benefit from non-operative management (NOM)

A number of LARC patients with a cCR to CRT still have cancer cells in their surgical specimens. The lack of a reliable method for identifying those residual cancer cells is the main obstacle to NOM in patients treated with neoadjuvant therapy. Clinical examination tends to underestimate tumor response to CRT.[48] Morphologic imaging modalities such as endorectal ultrasound (ERUS) and computed tomography (CT) provide a rough estimate of tumor regression, but cannot reliably distinguish post-treatment edema, fibrosis, and necrosis from residual tumor. [49] [22, 50] Functional studies such as FDG-PET with or without simultaneous CT are valuable in assessing partial tumor response, but are not sensitive enough to identify pCR.[51]

MRI has become the primary imaging tool for staging rectal cancer, but its role in assessing tumor response after CRT is still under investigation.[50, 52] Morphologic MRI criteria using conventional sequences for assessment of rectal cancer response to neoadjuvant therapy are based on the reduction of signal intensity, relative to pre-treatment images, that occur when tumor is replaced by fibrosis. However, changes in signal intensity correlate poorly with pCR. Several studies have shown that MRI tumor regression grade (mrTRG) is associated with good tumor response and improved survival in LARC patients treated with CRT. [50] Other studies have reported that a MRI-measured change in tumor volume (mrΔvolume) greater than -70% after CRT is associated with tumor response.[53, 54] However, the number of patients with pCR in these series was too small to make meaningful comparisons.

Diffusion weighted (DW) MRI is a functional imaging tool that captures the dynamic cellular-level motion of water, providing images with higher signal-to-noise ratio compared to conventional morphologic sequences like T_2-w MRI. Using special motion-probing magnetic

gradients, DW-MRI derives its image contrast from differences in the motion of water molecules in various tissues. The degree of restriction of water molecules is inversely correlated to tissue cellularity and cell membrane integrity. Restriction of the diffusion of water molecules can be considered a surrogate marker of tissue cellularity. A measurement of the restriction of the tumor's water molecules, known as the apparent diffusion coefficient (ADC), provides a semi-quantitative estimate of tissue cellularity.[55]

A number of investigators have studied the diagnostic performance of DW-MRI in assessing rectal cancer response to CRT.[42, 56-61] Most of these studies have used both qualitative (visual analysis of areas with high signal intensity) and/or quantitative (ADC measurements) evaluation(s) to define pCR. Kim et al reported that qualitative analysis of DW imaging improved the diagnostic accuracy of cCR compared to conventional MRI.[57] These results were later confirmed by Lambregts et al, who reported improvement in the diagnosis of cCR to CRT using DW imaging, compared to conventional MRI sequences.[42] These authors also reported a reduction in the number of equivocal cases, as well as improved interobserver agreement with DW imaging. A group in the Netherlands used DW-MRI to calculate tumor volume. They found that post-CRT DR-MRI volumetry and pre-post Δvolume were accurate in diagnosing pCR in patients with LARC treated with CRT.[62] Patients who obtain pCR have a higher ADC in post-CRT DW-MRI, and a greater change in ADC from pre-treatment to post-treatment DW-MRI, compared to non-pCR patients.[57, 60, 61] These data suggest that both visual analysis of signal intensity and changes in the ADC values in DW-MRI after neoadjuvant therapy, compared to baseline, may help identify patients who will benefit from NOM.

In this study we will prospectively evaluate the diagnostic performance of several conventional T_2-w and DW-MRI measurements to identify patients treated with TNT who will benefit from NOM. In previous studies, conventional and DW-MRI were performed at baseline, early in the course of CRT, and again 4 to 8 weeks after CRT. A unique aspect of our proposal is the timing of MRI studies within the treatment schema. Patients in both the INCT and CNCT arms will have conventional and DWI-MRI studies at baseline and at the time of tumor re-staging, approximately 30-40 weeks after the initiation of TNT. We expect that delaying evaluation until completion of TNT will increase the probability of identifying patients with a complete response, and help establish objective criteria to select patients for NOM. The results of the interval MRI examinations may also help elucidate the contribution of each component of TNT to tumor regression.

3.6 Circulating tumor DNA and miRNA

Several studies have shown that detectable circulating tumor DNA (ctDNA) before treatment or after surgical resection is associated with poor prognosis in colorectal cancers.[63, 64] Diehl and his colleagues found that circulating tumor DNA (ctDNA) measurements could be used to reliably monitor tumor dynamics in subjects with colorectal cancer who were undergoing surgery or chemotherapy, suggesting that ctDNA is a promising predictive marker in cancer patients.[65] They concluded that ctDNA seemed more sensitive and reliable than the standard marker, carcinoembryonic antigen (CEA), in the follow-up of patients with CRC. However, the information on using ctDNA to monitor tumor response to multimodality therapy in early or advanced colorectal cancer is limited. In this study we will investigate the feasibility of using ctDNA to monitor tumor response to CRT, and relapse after an apparent complete response.

miRNAs are small single-stranded non-coding RNAs (18-24 nucleotides in length) that negatively regulate their target gene expression by binding to complementary sites in their 3'-UTR. miRNAs play important roles in various biological processes such as development,

differentiation, proliferation, apoptosis, metabolism and morphogenesis, stress response, and diseases, including cancer.[66] In human cancers, miRNAs are deregulated and involved in tumor initiation, progression and metastasis.[67-72] miRNAs are differentially expressed in tumor tissues relative to paired normal controls, and their expression profiles are unique to specific cancer types.[73] miRNA are found in the plasma of cancer patients in a remarkably stable form, due to their binding of the Ago2 protein.[74] A number of studies have investigated the potential role of miRNA as novel biomarkers for early detection, prognosis and therapeutic response of colorectal cancers.[75, 76] In this study, we will use deep sequencing to measure miRNA expression in the tumor and plasma from rectal cancer patients before and after neoadjuvant treatment, and at different points during follow-up, to assess their role as predictors of tumor persistence and as prognostic factors.

3.7 Tumor Genotyping

Genetic profiling of rectal tumors has many potential clinical benefits. Identification of patients whose tumors have a genetic profile indicating resistance to chemoradiotherapy may spare these patients unhelpful neoadjuvant treatment, and they may proceed directly to surgery. On the opposite end of the spectrum, identification of tumors with a genetic profile amenable to a complete clinical or pathological response may help identify patients for whom surgery is unnecessary. We have previously shown that rectal tumors with mutations in both the KRAS and p53 genes are less likely to respond to CRT.[77] DNA polymorphisms have also been associated with rectal cancer response to pCR and toxicity to neoadjuvant therapy.[77, 78] In addition, we found that somatic DNA copy number alterations are associated with tumor response and lymph node metastasis in rectal cancer patients treated with CRT and TME.[79, 80]

Similarly to tumor genotyping, gene expression analysis can be used to predict response of a tumor to chemoradiotherapy. Gene expression analysis is already in clinical use for identification of breast cancer patients who will benefit from adjuvant chemotherapy. Evidence exists that variations in gene expression in rectal cancer may also predict tumor response to neoadjuvant treatment.[81, 82] In this study, we plan to validate these biomarkers of tumor response in a larger group of patients treated with CRT and surgery.

3.8 Molecular mechanism of tumor resistance to neoadjuvant therapy

Residual cancer stem cells are postulated to be a cause of cancer recurrence, as well as a cause of resistance to radiotherapy and chemotherapy. Evidence indicates that cancer stem cells are capable of long-term survival, and have strong regenerative potential.[83] These stem cells also appear to be resistant to ionizing radiotherapy and chemotherapy, due in part to their increased capacity for DNA repair.[84] The crypt base columnar cell (CRC) is one such stem cell. It is located in the crypts of normal colonic and rectal mucosa, and has shown resistance to radiotherapy. CRCs are characterized by exclusive cell surface expression of leucine-rich-repeat-containing G-protein-coupled receptor 5 (Lgr5+), a Wnt target that is often dysregulated in colon cancer.[85] Quantifying CRC within the tumor by immunohistochemical staining for Lgr5+ expression may facilitate the identification of tumor resistance to chemoradiotherapy, while CRC cells within the residual tumor scar in patients with a cCR may indicate that these patients are destined to recur either locally or distantly. Other cell markers postulated to be colonic stem cell markers include CD24, CD44, CD133, CD166, Olmf4, Aldh1, Integrins, Bmi1 and Musashi1.[86]

4.0 OVERVIEW OF STUDY DESIGN/INTERVENTION

4.1 Design

This is a multicenter phase II study investigating the efficacy of TNT and selective NOM in patients with LARC. Patients with MRI-staged Stage II (uT3-4, uN-) or Stage III (any T, N+) rectal cancer and would be considered to require a complete TME at baseline will be randomized to receive FOLFOX/CapeOX before (Arm 1 or INCT) or after (Arm 2 or CNCT) 5-FU or capecitabine-based CRT. An interval evaluation will be conducted after completion of INCT in Arm 1, and after completion of CRT in Arm 2. Patients with tumors progression at the time of the interval evaluation will be treated according to standard practice per treating institution guidelines. Those with evidence of stable disease or tumor response will complete neoadjuvant therapy. Patients in both arms will be re-staged after completion of all neoadjuvant therapy. Patients with incomplete response will undergo a TME. Patients with complete or near complete clinical response will be treated with NOM. Criteria for determining complete, near complete and no incompleteresponse are outlined in Section 9.3.2. Patients in both groups (TME and NOM) will be followed according to NCCN guidelines. For study purposes, all patients will be followed for five years from the last date of treatment. For patients in the TME group, last date of treatment will be the date of surgery. For NOM patients, including those who undergo subsequent TME, last date of treatment is the last date of neoadjuvant treatment. Patients in the NOM group will be evaluated using rectal MRI and endoscopic exam (see section 10). Patients in the NOM group who undergo subsequent TME will be followed according to current NCCN guidelines. All patients will be followed based on intent-to-treat. Disease-free survival will be measured after three years.

The study is designed to test the hypothesis that patients with LARC treated with TNT and TME or NOM will have an improved 3-year DFS compared to patients with similar tumors treated with CRT, TME and ACT. We will measure QoL in patients treated with TNT and TME with patients treated with TNT alone. We will also compare INCT to CNCT with respect to organ preservation at 3 years, compliance with treatment, adverse events, and surgical complications.

4.2 Intervention

The neoadjuvant chemotherapy regimen is prescribed specifically as 8 cycles of FOLFOX or 5 cycles of CapeOX over a period of approximately 15-16 weeks. The CRT regimen consists of the standard algorithms: a total of 5400-5600 cGy of radiation (4500 cGy to the pelvis, with an integrated boost to the primary tumor and involved nodes of 500 cGy, followed by an optional 400-600 cGy boost to the primary tumor and involved nodes) delivered in 27-28 fractions, respectively, of 180-200 cGy each over a 5-6 week period. Starting on the first day of RT, patients receive 5-FU administered by continuous infusion or capecitabine for the duration of the radiotherapy.

An interval evaluation will be conducted after completion of INCT in Arm 1, and after completion of CRT in Arm 2. Patients with tumor progression at interval evaluation will be treated according to standard practice as per treating institution guidelines; those with evidence of response or stable disease will complete neoadjuvant therapy. Patients in both arms will be re-staged after completion of all neoadjuvant therapy. Those with a clinical response insufficient to warrant non-operative management safely at the time of re-staging evaluation will undergo a TME. Patients with near complete clinical response or clinical complete response will be treated with NOM. Patients in both arms (TME and NOM) will be followed according to NCCN guidelines. Patients in the NOM group will be evaluated using rectal MRIs and endoscopic exam (see section 10). Patients in the NOM group who undergo subsequent TME will be followed according to current NCCN guidelines.

Pathology assessment of the surgical specimens will be performed according to the College of American Pathologists guidelines. Completeness of the mesorectal excision, tumor regression grade (TRG), and tumor staging will be categorized according to the criteria specified in the current edition of the AJCC Staging Manual.[87]

Safety will be assessed by documenting adverse events during TNT and after surgery. Adverse events will be graded according to NCI CTCAE version 4.0, and surgical complications will be graded according to the Clavien-Dindo classification.[88] Refer to Appendix N to see the classification scale.

Tissue and blood samples may be collected from all study patients. When clinically feasible, we will collect tumor tissue at baseline, and tumor and normal tissue samples from patients undergoing a TME. We will also collect blood at baseline and at different time points during and after treatment to measure circulating DNA and miRNA.

5.0 THERAPEUTIC/DIAGNOSTIC AGENTS

5.1 Oxaliplatin (OXAL)

5.1.1 Description

Oxaliplatin is an organoplatinum complex in which the platinum atom is complexed with 1,2-diaminocyclohexane with an oxalate ligand as a leaving group. Platinum content is 48.1% to 50.1%.

5.1.2 OXAL Drug Preparation/Administration/Dispensing

The powder is reconstituted by adding 10 ml (for the 50 mg, vials) or 20 ml (for the 100 mg vials) of water for injection, or dextrose 5% in water, which yields a 5 mg/ml solution. This solution is further diluted for infusion with 250 ml to 500 ml dextrose 5% in water. The reconstitution or final dilution must never be performed with a sodium chloride solution. Needles, syringes, catheters, or intravenous administration sets containing aluminum should not be used with oxaliplatin. As with other platinum compounds, contact with aluminum may result in a black precipitate. Oxaliplatin will be administered by intravenous infusion over 120 minutes or according to standard institutional guidelines. Infusion time may be prolonged (up to 6 hours) in patients experiencing pharyngolaryngeal dysesthesia.

5.1.3 Procedure for Handling Drug Spills

Consult the current OXAL prescriber information for detailed instructions on handling drug spills.

5.1.4 Storage and Stability

Intact vials should be stored at room temperature. Reconstituted solutions are stable for 24 hours under refrigeration. Solution further diluted in DSW is stable for 6 hours at room temperature or 24 hours under refrigeration.

5.1.5 Source of Drug

OXAL is commercially available as a multisource product. For this study, locally obtained commercial supplies of OXAL will be used.

5.1.6 Drug Accountability

Because the commercial drug will be used, accounting for OXAL drug supplies is not specifically required in this study.

5.2 5-Fluorouracil (5-FU)

5.2.1 Description

5-FU is a fluoropyrimidine antimetabolite considered to act primarily as an inhibitor of thymidylate synthase. 5-FU is supplied as a colorless-to-faint yellow solution in 10-mL single-use vials. Each 10 mL of solution contains 500 mg 5-FU, with pH adjusted to approximately 9.2 with sodium hydroxide.

5.2.2 5-FU Drug Preparation/Administration/Dispensing

5-FU is administered directly as an IV bolus injection and as a continuous infusion. No dilution is required.

5.2.3 Procedure for Handling Drug Spills

Consult the current 5-FU prescriber information for detailed instructions on handling drug spills.

5.2.4 Storage and Stability

5-FU vials may be stored at room temperature, but should be protected from light.

5.2.5 Source of Drug

5-FU is commercially available as a multisource product. For this study, locally obtained commercial supplies of 5-FU should be used.

5.2.6 Drug Accountability

Because the commercial drug is used, accounting for 5-FU drug supplies is not specifically required in this study.

5.3 Leucovorin

5.3.1 Description

Leucovorin is a mixture of the diastereoisomers of 5-formyl derivative of tetrahydrofolic acid. The active component is the (-)-L-Isomer known as Citrovorum factor. It is useful as an antidote to drugs which act as folic acid antagonists.

5.3.2 Leucovorin Drug Preparation/Administration/Dispensing

Leucovorin may be reconstituted with Bacteriostatic Water for Injection (BWI) or with Sterile Water for Injection. Solutions should be further diluted in DSW, 0.9% NaCl or Ringers solution for infusion over 2 hours. Leucovorin will be administered as a 400 mg/m2 IV infusion over 2 hours post oxaliplatin administration or according to standard institutional

guidelines. Leucovorin may also be administered concurrently with oxaliplatin, via same infusion lines or according to standard institutional guidelines.

5.3.3 Procedure for Handling Drug Spills

Consult the current Leucovorin prescriber information for detailed instructions on handling drug spills.

5.3.4 Storage and Stability

Intact vials should be stored at room temperature and protected from light. Solutions reconstituted with BWI are stable for at least 7 days at room temperature.

5.3.5 Source of Drug

Leucovorin is commercially available as a multisource product. For this study, locally obtained commercial supplies of Leucovorin should be used.

5.3.6 Drug Accountability

Because the commercial drug will be used, accounting for Leucovorin drug supplies is not specifically required in this study.

5.4 Capecitabine (Xeloda®)

5.4.1 Product description

Capecitabine is an oral prodrug of 5-fluorouracil (5-FU). 5-FU is a fluoropyrimidine antimetabolite considered to act primarily as an inhibitor of thymidylate synthase.

5.4.2 Formulation

Xeloda® is supplied as biconvex, oblong film-coated 500 mg tablets.

5.4.3 Storage requirements

Store tightly closed at 25 degrees C (77 degrees F); stable for brief periods at 15 to 30 degrees C (59 to 86 degrees F).

5.4.4 Route of administration

Tablets should be taken orally within 30 minutes after meals to improve absorption.

5.4.5 Source of Drug

Capecitabine is commercially available as a multisource product. For this study, locally obtained commercial supplies of Capecitabine should be used.

6.0 CRITERIA FOR SUBJECT ELIGIBILITY

6.1 Subject Inclusion Criteria

- Histologically confirmed diagnosis of adenocarcinoma of the rectum.
- Clinical Stage II (T3-4, N-) or Stage III (any T, N+) based on MRI
- Rectal tumor at baseline which would be considered to require complete TME
- No evidence of distant metastases
- No prior pelvic radiation therapy
- No prior chemotherapy or surgery for rectal cancer
- Age $\geq$ 18 years. The minimum legal age of consent for select Canadian provinces is 19
- No active infections requiring systemic antibiotic treatment (oral antibiotics are acceptable at the discretion of the treating physician)
- ECOG Performance status 0-2
- Women with childbearing potential (WOCBP) who are negative for pregnancy test (urine or blood) and who agree to use effective contraceptive method. A woman of childbearing potential is defined of one who is biologically capable of becoming pregnant. Reliable contraception should be used from trial screening and must be continued throughout the study.
- Patients must read, agree to, and sign a statement of Informed Consent prior to participation in this study. Patients who do not read or understand English are eligible and may be consented according to institutional and federal regulations.
- ANC > 1.5 cells/mm3, HGB > 8.0 gm/dl, PLT > 150,000/mm3, total bilirubin ≤ 1.5 x ULN (except in patients with Gilbert's Syndrome who must have total bilirubin ≤ 3.0 x ULN), AST≤ 3 x ULN, ALT ≤ 3 x ULN.

6.2 Subject Exclusion Criteria

- Recurrent rectal cancer
- Primary unresectable rectal cancer. A tumor is considered unresectable when invading adjacent organs and an en bloc resection will not achieve negative margins.
- Creatinine level greater than 1.5 times the upper limit of normal.
- Patients who have received prior pelvic radiotherapy.
- Patients who are unable to undergo an MRI.
- Patients with a history of any arterial thrombotic event within the past 6 months. This includes angina (stable or unstable), MI, TIA, or CVA.
- Patients with a history of venous thrombotic episodes such as deep venous thrombosis, pulmonary embolus occurring more than 6 months prior to enrollment may be considered for protocol participation, provided they are on stable doses of anticoagulant therapy. Similarly, patients who are anticoagulated for atrial fibrillation or other conditions may participate, provided they are on stable doses of anticoagulant therapy.
- Other Anticancer or Experimental Therapy. No other experimental therapies (including chemotherapy, radiation, hormonal treatment, antibody therapy, immunotherapy, gene therapy, vaccine therapy, angiogenesis inhibitors, matrix metalloprotease inhibitors, thalidomide, anti-VEGF/Flk-1 monoclonal antibody or other experimental drugs) of any kind are permitted while the patient is receiving study treatment.
- WOCBP who are unwilling or unable to use an acceptable method of avoiding pregnancy for the entire study period.
- Women who are pregnant or breast-feeding.

- Patients with any other concurrent medical or psychiatric condition or disease which, in the investigator's judgment, would make them inappropriate candidates for entry into this study.
- Patients with a history of a prior malignancy within the past 5 years, except for adequately treated basal cell or squamous cell skin cancer or in situ cervical cancer.

7.0 RECRUITMENT PLAN

Potential research subjects will be identified by a member of the patient's treatment team, the protocol investigator, or research team at Memorial Sloan Kettering Cancer Center (MSKCC) or other participating institution. If the investigator is a member of the treatment team, s/he will screen their patient's medical records for suitable research study participants and discuss the study and their potential for enrolling in the research study.

The principal investigator may also screen the medical records of patients with whom they do not have a treatment relationship for the limited purpose of identifying patients who would be eligible to enroll in the study and to record appropriate contact information in order to approach these patients regarding the possibility of enrolling in the study.

During the initial conversation between the investigator/research staff and the patient, the patient may be asked to provide certain health information that is necessary to the recruitment and enrollment process. The investigator/research staff may also review portions of their medical records in order to further assess eligibility. They will use the information provided by the patient and/or medical record to confirm that the patient is eligible and to contact the patient regarding study enrollment. If the patient turns out to be ineligible for the research study, the research staff will destroy all information collected on the patient during the initial conversation and medical records review, except for any information that must be maintained for screening log purposes.

In most cases, the initial contact with the prospective subject will be conducted either by the treatment team, investigator or the research staff working in consultation with the treatment team. The recruitment process outlined presents no more than minimal risk to the privacy of the patients who are screened and minimal PHI will be maintained as part of a screening log. For these reasons, we seek a (partial) limited waiver of authorization for the purposes of (1) reviewing medical records to identify potential research subjects and obtain information relevant to the enrollment process; (2) conversing with patients regarding possible enrollment; (3) handling of PHI contained within those records and provided by the potential subjects; and (4) maintaining information in a screening log of patients approached (if applicable).

The limited waiver will apply only to MSKCC. Any participating sites that require a limited waiver must obtain it from their own local IRB/Privacy Board (PB) via a separate protocol addendum or request. It is the responsibility of the MSKCC staff to confirm the participating data collection sites(s) have a limited waiver approved by their local IRB(s)/PB(s).

All patients meeting the eligibility requirements will be considered for enrollment regardless of sex, race, or religion. Eligibility criteria may not be waived by the investigator. Discussions regarding protocol enrollment and patient eligibility may begin with any of the investigators named on the protocol or their associates. Patients will be made aware of the protocol, its specific aims and objectives, and the potential risks and benefits that the patient may incur. Patients will be required to read, agree to, and sign an IRB-approved Informed Consent form prior to registration. There will be no financial compensation for patients enrolling in this protocol. Over 120 new patients with stage II or III rectal cancer are treated at MSKCC every year.

The study will be opened at other high-volume rectal cancer centers. The investigators from those institutions have embraced the concepts of TNT and NOM, and demonstrated their ability to accrue, having enrolled over 270 patients to the TIMING trial (PI: Garcia-Aguilar). Many of these investigators also accrued to the ACOSOG Z6041 trial (PI: Garcia-Aguilar) investigating the efficacy of CRT and local excision in patients with T2N0 rectal cancer.[89] It is expected that approximately 87 patients will be recruited at MSKCC and the remaining 238 will be recruited at the outside centers. Accrual rates are expected to vary across sites based on location and available staff support. Patients will be screened and enrolled at the participating sites. It is recommended that patients have all of their treatment at the primary enrolling site. However, they may receive some of their treatment at affiliated sites. An affiliated site is defined as one that is under the IRB of the enrolling institution.

Please refer to Section 15.0, Research Participant Registration and Randomization Procedures, for details on enrollment procedures.

8.0 PRETREATMENT EVALUATION

8.1 Overview of Pre-Treatment Evaluation

All patients will undergo pre-treatment staging via physical examination, procto/sigmoidoscopy, MRI, and pathologic assessment of the tumor. When clinically feasible, MSKCC patients will also have a biopsy and photograph taken of the primary tumor. Participating sites are encouraged to take photographs of the primary tumor when feasible, which should be submitted to the MSKCC Study coordinator, as outlined in section 16.0.1

Pre-treatment tumor measurements will be specified and recorded as baseline. Rectal MRI will be performed to obtain baseline evaluation of tumor, and will include standard measurements of tumor and nodal size as specified by the treating radiologist. Each patient will be evaluated by their medical oncologist to determine his/her suitability for therapy with FOLFOX/CapeOX, and by their radiation oncologist to determine his/her suitability for IMRT or other conformal radiation techniques.

8.2 Specific Evaluations to be completed within 45 calendar days (+/- 15 days) prior to treatment:

- Comprehensive evaluation by Surgical Oncology including:
    - History and physical examination
    - Endoscopic examination
    - Digital rectal exam
    - MSKCC Only: primary tumor biopsy and photograph, when clinically feasible
- Comprehensive evaluation by Medical Oncology
- Comprehensive evaluation by Radiation Oncology
- MRI of the rectum[1]
- CT Scan of chest, abdomen, and pelvis[1]

[1] Evalutions may be performed at outside institutions

  - o Contrast is preferred, but not required for CT of the chest. Unless contraindicated, contrast is REQUIRED for CT of the abdomen and pelvis.
  - o MRI of abdomen and pelvis with contrast may be used if CT scan results are inconclusive or if CT scan with contrast is contraindicated. A CT of the chest (contrast preferred, but not required) is required when an MRI of the abdomen and pelvis is performed.
  - o PET/CT scan ecompassing the chest, abdomen, and pelvis may be performed in lieu of a CT scan with contrast, but CT chest/abdomen/pelvis with contrast is preferred.

- Carcinoembryonic antigen (CEA)[1]
- EKG[1]
- Performance status (ECOG)
- Complete blood count with leukocyte differential[1]
- Comprehensive metabolic panel, at the discretion of the treating physician[1]
- Pathologic confirmation of adenocarcinoma at treating institution
- Pregnancy test for subjects of childbearing potential[1]

9.0 TREATMENT/INTERVENTION PLAN

9.1 Neoadjuvant Chemotherapy

There are several variations of the basic FOLFOX regimen. The specific version of the FOLFOX regimen that will be used in this trial is modified, or mFOLFOX6. This entails administration of 5-FU and leucovorin all on day 1 (in contrast to the FOLFOX4 regimen, where this is divided over 2 days) and administration of 85 mg/m^2 of oxaliplatin. Instead of referring to the regimen as mFOLFOX6, we refer to this neoadjuvant regimen, more simply, as FOLFOX.

Patients will receive 8 cycles of FOLFOX, administered every other week. FOLFOX will be given on Day 1 of each cycle. Patients will receive oxaliplatin 85 mg/m^2 IV (over 120 minutes or according to standard institutional guidelines), leucovorin 400 mg/m^2 IV (over 120 minutes or according to standard institutional guidelines), 5-FU 400 mg/m^2 IVP, and 5-FU 1200 mg/m^2/day CIVI over 2 days (total dose of 2400 mg/m^2 over 46-48 hours) for a total of 16 weeks/8 cycles.

For patients receiving CapeOX; Oxaliplatin will be delivered at 130 mg/ m^2 over two hours day 1 or according to standard institutional guidelines, and capecitabine at 1000mg/m^2 BID days 1-14. They will be repeated on a 21 day cycle, for a total of 15 weeks/5 cycles (maximum total cumulative oxaliplatin dose 650 mg/m^2). To track compliance, patients will be asked to complete a pill diary for each cycle of CapeOX (Appendix G).

In an effort to prevent adverse events, all patients will be treated with anti-emetics and supportive agents as per institutional guidelines. For patients treated outside of MSKCC, supportive and antiemetic agents may be administered per that institution's standard

practices. If the institution does not have a standard practice, then these may be administered at the discretion of the primary oncologist.

The FOLFOX and chemoradiotherapy regimens used in this clinical trial represent the standard care for treatment of patients with locally advanced rectal cancer. Therefore, all clinicians recruiting study subjects have considerable experience with this regimen. The toxicity of each specific agent is outlined below. The regimens used in this clinical trial also represent standard care for patients with locally advanced rectal cancer.

9.1.1 Neoadjuvant Chemotherapy Review/Quality Control

The following should be submitted to the MSKCC Study Coordinator (ski13213@mskcc.org or fax 646-227-7267) for all patients:

- Within 1 week of starting chemotherapy
  - Pre-treatment blood work/lab values
  - Chemotherapy order form
- Within two (2) weeks after completing chemotherapy
  - Blood work/lab values during treatment
  - Chemotherapy flow sheets (FOLFOX) or pill diaries (CapeOX)

The submitted flow sheets and pill diaries will be reviewed centrally for quality assurance purposes for the first 2 patients enrolled at each site. After the first two patients at each site, flow sheets/pill diaries will be reviewed centrally for quality assurance purposes on a case-by-case basis.

9.2 Chemoradiotherapy

9.2.1 Radiation Therapy Equipment and General Techniques

Linear accelerators with a minimum energy of 6MV will be used. A multiple-field technique using intensity modulated radiotherapy (IMRT) planning is recommended. 3-dimensional conformal radiotherapy (3d-CRT) using a 3 or 4-field plan is also acceptable. Radiation will be delivered 5 business days/week, once per day, at 180-200 cGy/day. Daily image guidance can be used, but is not required, to ensure accurate targeting of the tumor and draining lymph node regions.

9.2.2 Simulation and Immobilization

Radiation simulation utilizing CT-based planning will be performed prior to RT. If possible, patients will be treated in the prone position with a full bladder technique (patients drink 16 oz of water 1 hour prior to scan and 1 hour prior to daily treatment) if tolerated; supine position is acceptable if prone positioning is not tolerable for the patient. A suitable immobilization device (such as Aquaplast mold or Alphacradle) should be used if treating with IMRT. IV contrast should be administered to patients without contrast allergy or compromised kidney function. If IV contrast is not used, oral small bowel contrast should be administered. CT images will be obtained using 2.5-3 mm slice thickness.

9.2.3 Contouring and Target Volume Delineation

Gross tumor volume (GTV): This includes the primary tumor and any pelvic nodes believed to be involved grossly by metastatic disease. Assessment of the primary tumor and nodal

disease may be made on the basis of procto/sigmoidoscopy, CT, PET-CT, MRI, or ERUS. The entire rectal circumference at the level of the tumor should be included as GTV.

Clinical target volume (CTV): This includes the GTV and the following nodal groups: perirectal nodes; presacral nodes; internal iliac nodes; and common iliac nodes below the L5-sacral junction. If the tumor is a clinical T4 with anterior extension into an adjacent organ (i.e. prostate, cervix, bladder, vagina), the CTV should include the external iliac nodal regions. For patients with tumors invading the anal sphincter, the CTV should include the bilateral inguinal nodes and external iliac nodes. Please refer to the RTOG Anorectal Contouring Atlas:
(http://www.rtog.org/LinkClick.aspx?fileticket=DgflROvKQ6w%3d&tabid=231)

Organs at risk: Small bowel, large bowel, bladder, vagina, external genitalia, and femoral heads should be contoured.

<u>IMRT Planning</u>:

Planning target volume 4500 (PTV 45): This will provide a margin around the CTV to compensate for the inter- and intra-fraction uncertainty consequent to daily setup uncertainty, and to potential internal organ motion. By definition, the PTV will consist of a 5 mm expansion around the CTV.

Planning target volume 50 (PTV 50): The PTV for the integrated boost volume is an expansion of the GTV by 1 cm–2cm, modified to include the presacral region (posterior mesorectal region).

Optional Boost: planning target volume Boost (PTV Boost): An additional boost of 2-3 fractions of 200 cGy may be delivered to the primary tumor, bringing the total dose to the primary tumor to 5400cGy to 5600cGy over a total of 27 to 28 fractions, respectively. This boost volume typically is the same as the PTV50, though margins may be made smaller to exclude bowel from the field. If the boost volume would encompass a significant volume of small bowel, this optional boost should not be given (i.e. the total primary tumor dose should remain 50Gy). .

<u>3 or 4-Field Treatment Planning for 3D-CRT:</u>

For the first course, use of a posterior-anterior (PA) field and opposed laterals is recommended. An anterior-posterior (AP) field may be used at the discretion of the treating radiation oncologist if it results in improved dose homogeneity without increasing the small bowel dose unnecessarily.

Field borders for PA (and AP if used) field:
- Inferior – At least 3 cm below the inferior extent of the cancer, or at least 1cm below the level of the pelvic floor for cancers located ≤5 cm from the anal verge based on digital rectal examination or MRI, whichever is lower (refer to the RTOG Anorectal Contouring Atlas referenced above). The anal verge should be identified by a marker on simulation.
- Lateral – 1.5-2 cm lateral to the bony pelvis taken at its widest point.
- Superior - L5-S1 junction.

Field borders for opposed lateral fields:
- Superior - To correspond to PA fields.
- Inferior - To correspond to PA fields.

- Anterior - This will cover the lower common and internal iliacs as defined on CT scans. Anatomically this will extend anteriorly to approximately the anterior one-third of the acetabulum. For T4 tumors extending anteriorly or tumors with anal canal involvement, IMRT planning is recommended.
- Posterior – Behind the sacrum.

The initial cone down field of 3 fractions to 540cGy shall have a 2-3 cm margin around the GTV but must include the whole of the sacral hollow.

An optional, additional boost of 2-3 fractions of 180cGy (360cGy – 540cGy) can be added to bring the total dose to 5400-5580cGy, using similar guidelines as described in the IMRT section.

.

9.2.4 Treatment Planning Techniques

All treatment planning will be performed with computerized dosimetry and the dose should be prescribed to the isodose line that covers the treatment volume at risk. The PTV45 should be encompassed within the isodose surface corresponding to 95% of the prescription dose for that volume. Calculations shall take into account the effect of tissue heterogeneities.

9.2.4.1 Prescription Dose and Fractionation

<u>IMRT Planning</u>:

- PTV45: The total dose to the PTV45 will be 4500 cGy delivered in 25 fractions of 180 cGy per day.
- PTV50: An integrated boost will be delivered to the GTV with margin delivered in 25 fractions at 200 cGy per day (total dose 5000 cGy).
- Optional PTV Boost: A cone-down dose of 400-600 cGy will be delivered to PTV Boost in 2-3 fractions of 200 cGy per day (total dose 5400-5600 cGy).

<u>3 or 4-Field Treatment Planning</u>:

- PTV45: The total dose to the PTV45 will be 4500 cGy delivered in 25 fractions of 180 cGy per day.
- PTV50.4: Cone-down will be delivered to the GTV with margin delivered in 3 fractions at 180 cGy per day (total dose 5040 cGy).
- Optional PTV Boost: A cone-down dose of 360 cGy – 540cGy will be delivered to PTV Boost in 2-3 fractions of 180 cGy per day (total dose 5400-5580 cGy).

9.2.4.2 Dose Constraints for Target Volumes and Organs at Risk (OAR)

For patient without boost:

PTV45:

Max dose < PTV50 max dose
V(5000cGy)< 10% (exclusive of the PTV50 volume)
D(95%) ≥ 4500cGy
V(4275cGy) ~ 100%

PTV50:

Max dose < 5500cGy
D(95%) ≥ 5000cGy

For patients with boost:
PTV45:
Max dose < PTV50 max dose
V(5000cGy)< 10% (exclusive of the PTV50 volume)
D(95%) ≥ 4500cGy
V(4275cGy) ~ 100%

PTV50:
Max dose ≤ PTVmax boost dose
D(95%) ≥ 5000cGy

PTVBoost
Max dose < 5940cGy (for Prescription dose of 54Gy) – 6160cGy(for Prescription dose of 56Gy)

Critical structures
Small Bowel: V45Gy < 100cc, V50Gy < 10cc
Large Bowel: V45Gy < 135cc, V50Gy < 45cc
(Note: if bowel constraints cannot be met without compromising PTV coverage, the optional boost should NOT be given. In this circumstance, the use of IMRT and minimal acceptable margins is also strongly encouraged. If, due to patient anatomy, bowel constraints cannot be met without compromising PTV coverage, PTV coverage should be maintained and the plan will typically be reviewed and approved as a deviation.)

Bladder:
Max bladder dose < Max PTV dose
Mean bladder dose < 35Gy (guideline, not to be prioritized over PTV coverage)

Vagina (females):
No more than 85% of the volume can receive 4500cGy – V(4500cGy) ≤ 85%
(Note: if this constraint cannot be met without compromising PTV coverage, due to proximity of the GTV to the vagina, then PTV coverage should be maintained and the plan will typically be reviewed and approved as a deviation.)

Femurs:
Max ≤5000cGy

Cauda:
Max ≤ 5000cGy

9.2.5. Radiation Therapy Review/Quality Control

Within one week of starting radiotherapy, simulation and treatment planning data must be submitted to the coordinating center, including:

- Copies of pre-treatment CT and MRI reports identifying the location of the primary rectal tumor.
- Isodose distributions for the composite treatment plan in the axial, sagittal and coronal planes at the center of the treatment or planning target volume. The planning target volume, isocenter and the normalization method must be clearly indicated.

- Dose volume histograms (DVH) for the composite treatment plan for all target volumes and required organs at risk. A DVH shall be submitted for the organs at risk specified above.
- Dose constraints are as per section 9.2.4.2. Please complete the corresponding dosimetry form and submit to the MSKCC Study Coordinator (ski13213@mskcc.org or fax 646-227-7267).
- Treatment planning system summary report that includes the monitor unit calculations, beam parameters, calculation algorithm, and volume of interest dose statistics.

Simulation and treatment planning data should be submitted to MSKCC via the Secure File Transfer Protocol (SFTP) account. This account may be accessed through the following link: https://securetrans01.mskcc.org/webclient/Login.jsf. If SFTP submissions are not feasible, hardcopies can be mailed to the MSKCC Study Coordinator at the address below or emailed securely to (ski13213@mskcc.org).

Attn: MSKCC Study Coordinator
Memorial Sloan Kettering Cancer Center
Department of Surgery, Colorectal Service
633 3rd Ave, 15th Floor
New York, NY 10017

9.2.6 Chemosensitization during radiotherapy

Continuous infusion 5-FU or capecitabine are acceptable alternatives for chemosensitizing during radiation therapy.

5-FU 225 mg/m^2 per day by continuous IV infusion 7 days per week, administered concurrently with RT.

Capecitabine 825 mg/m^2 bid orally; 5 days per week on days of planned RT, orally administered concurrently with RT (Total capecitabine daily dose may be rounded to the nearest 500 mgs to allow for tablet size. If uneven number of tablets (i.e. 7 tablets daily), then the larger number will be taken with the morning dose (i.e. 4 tablets q a.m. and 3 tablets q p.m.)

Patients receiving capecitabine or 5-FU should be counseled in the management of diarrhea, mucositis, the potential for chest pain, tachycardia or other rhythm disturbances, and rash on the hands/soles. Precaution and limited sun exposure are to be advised. The importance of adherence to capecitabine and the need to alert providers in the event of any malfunction of infusion pump equipment must be reviewed with the patient. Patients will be asked to maintain a pill diary (Appendix H) to track compliance of capecitabine taken during radiation.

9.2.6.1 Sensitizing Chemotherapy Review/Quality Control

The following should be submitted to the MSKCC Study Coordinator (ski13213@mskcc.org or fax 646-227-7267) within 2 weeks of CRT completion for all patients:

- Flow sheets for the patients who receive 5-FU
- Pill diaries for the patients who receive capecitabine

The submitted flow sheets and pill diaries will be reviewed centrally for quality assurance purposes for the first 2 patients enrolled at each site. After the first two patients at each site, flow sheets/pill diaries will be reviewed centrally for quality assurance purposes on a case-by-case basis.

9.3 Treatment Evaluation

9.3.1. Treatment evaluation during TNT

Patients in Arm I will undergo a digital rectal exam and proctoscopic exam within 4 weeks (+/- 14 days) after the last day of chemotherapy of the last cycle (disconnect date of FOLFOX cycle 8 or the last day of capecitabine of CapeOX cycle 5). Patients in Arm 2 will undergo digital rectal exam and proctoscopic exam within 4 weeks (+/- 14 days) after the last day of radiation. If the tumor cannot be reached on digital rectal exam, this should be noted on the checklist and evaluation. Thus, the below criteria for digital rectal exam should only be applied for palpable tumors.

The primary tumor and the regional lymph nodes will be evaluated by digital rectal exam, and evaluated and measured by endoscopic exam. MSKCC patients will also undergo an MRI exam. MRI at the interval evaluation is optional for sites.

The following criteria should be used to best describe response during neoadjuvant treatment. The characteristic(s) which define the lowest level of response (furthest to the left on the schema below) will be used to define response. Checklists will be provided and the data will be entered into a central database.

	**Progressive Disease**	**Stable Disease or Partial Response**	**Near Complete Response**	**Complete Response**
**Endoscopic Exam**	• Increase in tumor size	• Partial response<br>• No change in size	• Irregular mucosa<br>• Small mucosal nodules or minor mucosal abnormality<br>• Superficial ulceration<br>• Mild persisting erythema of the scar	• Flat, white scar<br>• Telangiectasia<br>• No ulcer<br>• No nodularity
**Digital Rectal Exam**	• Palpable tumor nodules	• Palpable tumor nodules	• Smooth induration or minor mucosal abnormalities	• Normal
**MRI-T2W**	•	• No change in extent of T2 scar if present or further regression in thickness of T2 scar or intermediate tumor<br>• No new immediate signal not thought to be mural edema	• Mostly dark T2 signal, some remaining intermediate signal AND/OR<br>• Partial regression of lymph nodes	• Normal appearing bowel wall without any fibrosis in the tumor bed<br>• Only dark T2 signal, no intermediate T2 signal<br>• No visible lymph nodes or

		• Stable or further decrease in nodal size (no new nodes)		very few, small (<5mm nodes)
**MRI-DW**	•	• Stable or continued diminution in signal (no new focal signal)	• Significant regression of signal on B800-B1000	• No visible signal on B800-B1000 AND/OR<br>• Uniform, linear signal in wall above tumor is acceptable

Patients who present with stable disease, partial response, near-complete or complete clinical response should continue TNT. Patients who present with tumor progression should be treated according to standard practice per institutional guidelines. The total time between treatment arms can be up to but not more than 8 weeks.

9.3.2. Treatment evaluation at completion of TNT

Patients in both groups are required to undergo tumor assessment and re-staging by endoscopic exam, digital rectal exam, rectal MRI, and evaluation of potential metastatic disease by CT of the chest, abdominal, and pelvis, within 8 weeks (+/- 4 weeks) after completion of TNT (last day of radiation for Arm 1, and disconnect date for FOLFOX or last capecitabine for CapeOX in Arm 2).

The following criteria should be used to determine tumor response to TNT at the end of treatment. The characteristic(s) which define the lowest level of response (furthest to the right on the schema below) will be used to define response. Checklists will be provided and the data will be entered into a central database.

	**Complete Response**	**Near Complete Response**	**Incomplete Response**
**Endoscopic Exam**	• Flat whitish scar<br>• Telangiectasia<br>• No ulcer<br>• No nodularity	• Irregular mucosa<br>• Small mucosal nodules<br>• Superficial ulceration<br>• Mild persisting erythema of the scar	• Visible tumor
**Digital Rectal Exam**	• Normal	• Smooth induration or minor mucosal abnormalities	• Palpable tumor nodules
**MRI-T2W**	• Normal appearing bowel wall without any fibrosis in the tumor bed<br>• Only dark T2 signal, no intermediate T2 signal<br>• No visible lymph nodes or very few, small (<5mm nodes)	• Mostly dark T2 signal, some remaining intermediate signal AND/OR<br>• Partial regression of lymph nodes	• More intermediate than dark T2 signal, no T2 scar AND/OR<br>• No regression of lymph nodes

**MRI-DW**	• No visible signal on B800-B1000<br>AND/OR<br>• Uniform, linear signal in wall above tumor is acceptable	• Significant regression of signal on B800-B1000	• Insignificant regression of signal on B800-B1000

*Incomplete response indicates that tumor has not shrunk sufficiently to implement non-operative management safely. This category applies to tumors which do not meet the Complete Response or Near Complete Response criteria.

In general, clinical examination will prevail over imaging assessment of primary tumor response.

Patients with complete or near complete clinical response at the time of re-staging after TNT will be entered into the NOM protocol. Patients with incomplete response will proceed to TME. Incomplete response is defined as having neither a near complete response nor complete response.

The MSKCC PI and radiologist are available to assist with resolving local discrepancies.

9.3.3 Treatment decision during subsequent re-evaluations (NOM patients only)

Patients with sustained response compared to the previous evaluation will remain in the NOM follow-up arm. Patients with progressive disease in relation to their previous evaluation will undergo TME. Checklists for follow-up assessments will be provided and the data will be entered into a central database.

Biopsies of the primary tumor sites for patients on the NOM arm may be performed as clinically indicated by the investigators. A new positive biopsy (surgical, thru-cut or FNA) result for adenocarcinoma is considered to be tumor recurrence.

For follow-up rectal MRIs, the characteristics listed in the schema below should be used for evaluating patients for signs of re-growth in conjunction with exam by the treating surgeon. The characteristics listed below do not apply to mucinous tumors.

	**Signs of Re-Growth**
**MRI-T_2W**	❑ New intermediate T_2 signal, no T_2 scar<br>AND/OR<br>❑ New lymph nodes<br>AND/OR<br>❑ Increased size of lymph nodes
**MRI-DW**	❑ New signal on B800-B1000

9.3.4 Guidelines for tumor measurement

9.3.4.1 Endoscopic Exam

The length of the tumor is defined as the difference between the distance of the proximal and distal margins of the lesion in relation the anal verge.

Participating sites are encouraged to take photographs of the primary tumor site when feasible and submit these photographs to MSKCC. Photographs should be submitted in color when possible. All patient identifiers must be removed and the patient's unique study ID number included. Electronic copies of photographs should be submitted using the study SFTP account or emailed securely to the MSKCC Study Coordinator (ski13213@mskcc.org). Alternatively, hardcopies can be mailed to the MSKCC Study Coordinator at the address below.

Attn: MSKCC Study Coordinator
Memorial Sloan Kettering Cancer Center
Department of Surgery, Colorectal Service
633 3rd Ave, 15th Floor
New York, NY 10017

The treating surgeon will be asked to send endoscopic and clinical exam notes to MSK within 7-10 business days of the exam. Source documentation will be sent to the MSKCC Study Coordinator (ski13213@mskcc.org or fax 646-227-7267). The submitted endoscopic and clinical exam notes will be reviewed centrally at MSK for quality assurance purposes for the first 2 patients enrolled at each site. After the first two patients at each site, endoscopic and clinical exam notes will be reviewed centrally for quality assurance purposes on a case-by-case basis.

9.3.4.2 MRI/CT

Standard and DW-MRI sequences will be obtained in 1.5T or 3T units by using a phased-array body coil. All imaging studies will be interpreted by expert radiology staff at the patient primary treatment center for patient eligibility, clinical staging, and tumor response, according to standard clinical criteria. Central radiology review will be performed to ensure consistency with tumor staging and assessment of response. Images will be submitted on discs to the coordinating center (MSKCC). Baseline images should be submitted within 5 business days of the consent with report. Images for the first two patients at each site will be reviewed by central radiology review at MSKCC for quality control purposes. For interval evaluation and restaging evaluation exams, images should be submitted within 10 business days of the exams with reports. Submitted reports should indicate N staging. N staging of N- or N+ are accepted. Discs will be submitted de-identified and in the DICOM format. All patient identifiers must be removed and the unique case number included. Disks should be mailed to the MSKCC Study Coordinator at the following address.

Attn: MSKCC Study Coordinator
Memorial Sloan Kettering Cancer Center
Department of Surgery, Colorectal Service
633 3rd Ave, 15th Floor
New York, NY 10017

9.4 Biospecimens

Patients will be consented to use their normal and tumor tissue, including blood, as part of this study. Because the results generated by the analysis of the specimens are not currently

anticipated to have relevance to patients or family members, the results will not be disclosed to patients or their families.

Tumor biopsies will be obtained in the colorectal surgery clinic via endoscopic exam at baseline, in all patients when clinically feasible. Alternatively, tumor biopsies may be obtained in the operating room if a biopsy during endoscopic exam is not possible. Specimens will be sent to pathology. Additional samples will be taken from the surgical TME specimens. Sections will be reviewed by the study pathologist at MSK to ensure that samples contain adequate cellularity. Ten slides at 10 microns each will be collected from each tissue block.

For research purposes, one to two additional 10 ml EDTA tube(s) of blood will be collected when clinically feasible from patients before the initiation of treatment (for DNA isolation and plasma) and at different time points during treatment and follow-up. These time points will correspond to times when labs are drawn according to standard practice. The tubes will be transported to the laboratory, where the serum and plasma will be separated and stored.

In the course of this research it is possible that some patients whose tumors are analyzed through investigational “next-generation” profiling in a research (non-CLIA) environment will be found to have somatic or germline mutations in genes that are known to be associated with an increased risk of cancer or other diseases. It will be stated in the consent that the participants will not receive any specific results from research tests. The consent will tell participants that if they wish to have genetic testing done for personal reasons than they should make an appointment with the MSK Clinical Genetics Service.

If in the course of this research a research finding is obtained that, in the opinion of the investigator, may be critical to the preventive care of the participant or their family, the investigator can communicate that finding to the IRB Genomic Advisory Panel (GAP). The finding will be reviewed by the GAP to determine whether the incidental finding should be discussed with the participant. For MSK, in the event that the GAP determines that the finding should be discussed with the participant, and the participant has consented to be re-contacted, then the treating/consenting physician shall be contacted by the panel and asked to refer the participant to the Clinical Genetics Service for further discussion of the research finding.

The following information must be provided to GAP for review:

- Participant Name/MRN #
- Type of Biospecimen (tissue, blood, saliva)
- Incidental Finding
- Collection Protocol #
- Contact: rtmgapirb@mskcc.org

9.4.1 Tumor Genotyping

The Geoffrey Beene Translational Oncology Core Facility will be responsible for processing of clinical samples including DNA and RNA extraction, genotyping and gene expression analysis. This facility is located on the 6th floor of the Mortimer Zuckerman Research Building at MSKCC, and comprises five processing rooms fully equipped to perform state-of-the-art genome-scale molecular profiling. The Core Manager, Dr. Adriana Heguy, is an expert in genetics and genomics, and has vast experience in the management and analysis

of large data sets generated by automated high throughput technologies, acquired in biotechnological and academic settings. The Core is staffed with four technicians and one bioinformatician. The main automation equipment consists of one Biomek FX with capacity for setting up 27,648 PCR reactions daily, two Biomek NX, and one Biomek 3000, all custom-configured for high throughput DNA extractions, high throughput set-up of PCR reactions and for cherry-picking, an automated plate sealer and a Duncan water bath thermal cycler with three computer controlled water baths to modulate the temperatures of the PCR reaction, and a robotic arm to move the plates between water baths, with capacity for running 24 plates (9,216 PCR reactions) in ~ 2 hours. The Core laboratory also has a full Sequenom MassARRAY® compact system with Server and RT workstation, including: MassARRAY Analyzer Compact MALDI-TOF mass spectrometer for separation, detection and characterization of the analytes, the Nanodispenser RS 1000, and a Matrix PlateMate 2X3 for the post-PCR liquid handling steps. It is licensed to run genotyping, as well as DNA methylation analysis, using the MassARRAY system. The Core also has a NanoString nCounter system, including the nCounter Prep Station and the nCounter Analyzer for gene expression, copy number variation and miRNA, using color-coded molecular barcodes that can hybridize directly to nucleic acids.

9.4.3 Stem Cell Analysis

Tumor stem cell assessment will be made using immunohistochemical techniques. Following fixation of the tumor specimens in paraffin blocks at the hospital pathology laboratory, sections of the tumor will be cut from the blocks and stained with the stem cell markers Lgr5, CD24, CD44, CD133, CD166, Olmf4, Aldh1, Integrins, Bmi1 and Musashi following optimization of these antibodies. All immunohistochemistry will be performed in Dr. Paty's lab.Slides will be analyzed for expression of the respective stem cell markers.

9.5. MRI

All MRIs must meet the following minimum requirements. Appendix F lists in detail the technical and quality requirements for MRI Scans.

- 1.5 or 3.0 Tesla machine
- Use of phased array coil
- T2-Weighted axial, sagittal, and coronal images small field of view: 14-24 cm
- T2-Weighted axial and sagittal images maximum slice thickness: 3-4mm (5mm slice will not be accepted)
- T2-Weighted images should not be fat saturated
- Large field of view axial T1 and T2 from aortic bifurcation to inguinal region slice thickness: 5-7mm accepted

Baseline MRI images will be collected for the first two patients enrolled at each participating site to assess image quality. Baseline MRI images should be submitted to the MSKCC Study Coordinator within 5 business days of patient consent. If the MRI does not meet quality standards, feedback will be returned to the site with 48 hours. Quality and central imaging review will be performed at MSK. For detailed technical and quality requirements for MRI scans, please see Appendix F.

At baseline only, MRIs may be performed at a local imaging center external to a participating site, however they must be reviewed by the site radiologist and submitted to MSK for central review and approval. Disks should be submitted at least two weeks prior to treatment start, or

to allow enough time to re-scan the patient prior to treatment start, if necessary. This is in the event that outside MRIs do not follow the MRI requirements outlined in Appendix F.

MRI images taken at interval evaluation are required for all MSK patients and recommended for participating sites. For all patients with images taken at the interval and re-staging evaluations we are asking sites to submit these images to MSK for central review. For radiologic exams done during the interval and restaging evaluations, images should be submitted within 5 business days of the date of review by the participating site radiologist. These images should be de-identified, labeled with the unique study number, and submitted to the MSKCC Study Coordinator on disks in DICOM format. Submitted MRI reports should indicate staging and if nodes are positive or negative. N staging of N- or N+ are accepted. If the MRI does not meet the technical and clinical standards outlined in Appendix F, feedback will be returned to the site within 24 hours. Local and central interpretation will be tracked.

Discrepancies between clinical examination and imaging will be resolved by the local investigator. In the event that the MSKCC radiologist disagrees with a participating site interval or re-staging assessment and feels that it may place the patient at undue risk at the interval or restaging , these discrepancies will be communicated back to the participating site PI and radiologist. No response will be communicated back to the participating site if, upon review of submitted images, there are no discrepancies in assessment.

9.5.1 Volumetry

Tumor will be identified on 3 types of sequences for volumetry: T2W, DWI and DCE. On each axial slice where tumor is recognized, a free-hand ROI will be traced to enclose tumor and any necrosis, excluding vessels, artifact and luminal contents. The area, automatically calculated by the picture archiving and communications systems (PACS), will be multiplied by number of slices and slice thickness to derive the cm^3 volume.

9.5.2 DW-MRI

DW-MR images will be acquired with the standard single-shot spin-echo EPI (SE-EPI) sequence, with a pair of rectangular-shaped gradient pulses along three orthogonal axes. The nominal DW-MRI parameters will be: TR=3000 ms, TE= minimum, acceleration factor = 2, number of averages (NEX) = 4, FOV 24–36 cm, matrix 128 x 128. The scan location, orientation, and coverage will mimic the DCE-MR acquisition. DW images will be acquired with at least three diffusion-sensitizing factors (or b-values): 0, 400, and 800 s/mm^2. The imaging time for this portion of the exam will be 7-8 minutes.

Based on the standard mono-exponential model of diffusion, apparent diffusion coefficient (ADC) will be calculated at each b-value: ADC_{400}, and ADC_{800}. In addition, a revised mono-exponential model using data from all three b-values will be used to estimate true diffusion D, and perfusion fraction f. The true diffusion coefficient provides an additional DW-MRI parameter and is less influenced than the ADC by b-value selection. All analysis will be performed using in-house dedicated software written in Matlab (MATLAB®, MathWorks, Natick, MA, USA). Functional maps of all estimated parameters (ADC, D, and f) will be constructed and made available for further analysis. A ROI is drawn by the radiologist to include the entire tumor at its greatest cross-sectional diameter.

Using custom-made software, the 25th and 50th (median) percentile values, kurtosis, and skewness of the ROI histograms are computed. Kurtosis provides a quantitative measure of how sharply peaked a histogram is compared with the histogram of a normal distribution. Accordingly, whereas a normal distribution has a kurtosis of 0, a more peaked histogram has

a positive kurtosis value. Skewness provides a quantitative measure of the degree of asymmetry of a histogram: a perfectly symmetric histogram has a skewness of 0; a histogram with a long right tail has a positive skewness; a histogram with a long left tail has a negative skewness.

9.6 Bowel Function and Quality of life Assessment

Function and QoL will be assessed at registration, at re-staging after TNT, and at the following long term follow-up time points: 3-6, 9-12, 21-24 and 36 months +/-30 days Patients will receive one survey packet, including 32-45 items depending on patient gender and the presence of a stoma. We will use the 19-item Bowel Function Index (BFI) (available at http://www.ncbi.nlm.nih.gov/pubmed/15868235) to assess bowel function.[44] The psychometric properties of the instrument have been previously published [44] and all subscales and total score show good internal consistency (Cronbach's alpha: Frequency 0.75, Dietary 0.75, Soilage 0.78) and test-retest reliability (TOTAL 0.84 , Frequency 0.74, Dietary 0.62, and Urgency/Soilage 0.87).

All patients without a stoma will also complete 5 additional questions from the Low Anterior Resection Scale. The Low Anterior Resection Score (LARS) is a newly developed and validated 5 question index that was specifically developed to categorize bowel dysfunction after low anterior resection in Europe. Most recent use of the LARS data shows a good correlation and a high sensitivity (72.5%) and specificity (82.5%) for major LARS (poor function). Discriminant validity showed significant differences between groups expected groups. [97, 98] The use of LARS will allows us to compare our results to European groups. In the LARS Score patients are asked to describe symptoms which best describe daily life. There is no specific time period, but at most the averages are over the past week. The LARS score has a range of 0-42 with 0-20 being No LARS, 21-29 being Minor LARS, and 30-42 being Major LARS.

APR patients will complete 10 items from the BFI which is currently being used by others (2R01CA106912-04) and appears to have good psychometric properties. We will also use the 21-item Stoma QoL for patients undergoing an APR for descriptive purposes.[90] To assess sexual function, we will use gender-specific questionnaires: 5-item International Index of Erectile Function (IIEF-5), and 6-item Female Sexual Functioning Index. [91-93] Bladder function will be assessed using two items from validated questionnaires (the Prostate Health Related QOL questionnaire and the International Prostate Symptom Score (IPSS).

We will assess global health-related QoL using the EuroQOL5D-5L (available at http://www.euroqol.org/home.html), which evaluates domains of pain, anxiety, physical function, ambulation and activities of daily living, as well as the linear analogue scale.[94] These instruments, also embedded in the PROSPECT trial, reproducibly assess health-related QoL in a succinct manner. The entire functional assessment can be completed in less than 30 minutes, with an average of 15-20 minutes per patient. Patients may be contacted by phone for follow-up surveys. MSKCC patients may also be contacted via e-mail using the WebCore system. For patients who undergo TME after re-staging, QoL surveys should be administered at their 6, 12, 24, and 36 month follow-up visits. Patients placed on NOM who undergo subsequent TME for recurrent or persistent disease will re-start their post-treatment QoL survey cycle following surgery for up to five years from the last date of neoadjuvant therapy. These instruments are included in Appendices A-E.

10.0 EVALUATION DURING TREATMENT/INTERVENTION

**Evaluations during treatment – ARM 1 (INCT)**					
	Baseline/ Pre-treatment	Chemotherapy[1]	Interval Evaluation	Radiation Therapy	Re-Staging Evaluation
	45 calendar days ± 15 days prior to treatment start	FOLFOX or CapeOX	4 weeks ± 14 days from completion of chemotherapy		8 weeks ± 4 weeks after completion of radiation therapy
Eligibility/Consent	x				
Physical Exam/ECOG[2]	x				
DRE	x		x		x
Procto/Sigmoidoscopy	x		x		x
MRI Rectum[3]	x		x		x
CT CAP[4]	x				x
EKG	x				
CBC & differential[5]	x				
Comp & CEA[6]	x		x		x
Pregnancy Test	x				
Research blood[7]	x		x		x
QoL[8]	x				x

**Evaluations during treatment – ARM 2 (CNCT)**					
	Baseline/ Pre-treatment	Radiation Therapy	Interval Evaluation	Chemotherapy[1]	Re-Staging Evaluation
	45 calendar days ± 15 days prior to treatment start		4 weeks ± 14 days from completion of Radiation therapy	FOLFOX or CapeOX	8 weeks ± 4 weeks after completion of chemotherapy
Eligibility/Consent	x				
Physical Exam/ECOG[2]	x				
DRE	x		x		x
Procto/Sigmoidoscopy	x		x		x
MRI Rectum[3]	x		x		x
CT CAP[4]	x				x
EKG	x				
CBC & differential[5]	x				
Comp & CEA[6]	x		x		x
Pregnancy Test	x				
Research blood[7]	x		x		x
QoL[8]	x				x

[1] FOLFOX is 8 cycles, and each cycle is 14 days. CapeOX is 5 cycles, and each cycle is 21 days.

[2] ECOG status is required only at baseline. Patients will be evaluated by medical oncology during chemotherapy every cycle or as needed. Patients will be evaluated by radiation oncology during radiation therapy as needed.

[3] MSKCC patients only: MRI Rectum is required at the interval evaluation. MRI Rectum is recommended, but not required, for patients enrolled at participating sites.

[4] CT of the Chest, Abdomen and Pelvis. This is done at baseline and after completion of TNT to rule out metastatic disease.

[5] Complete Blood Count (CBC) & automated differential may be performed after baseline at the discretion of the treating physician.

[6] Comprehensive metabolic panel may be performed after baseline at the discretion of the treating physician. CEA is required at baseline and restaging but is optional at interval evaluation.

[7] MSKCC patients only: One to two additional 10 ml EDTA tube(s) of blood may be collected before treatment starts and after TNT is completed (Arms 1 and 2). One to two additional 10 ml tube(s) may be taken at an additional time point during treatment at the time labs are taken according to MSKCC standard practice.

[8]The QoL is required for patients who are fluent in reading and speaking English accrued at US and English-speaking Canadian sites. Sites must administer appendices approved by MSKCC's IRB, as indicated by the MSKCC IRB/PB watermark.

**Evaluations during follow-up for NOM patients**										
Months after treatment	3-6	9-12	15-18	21-24	30[1]	36[1]	42[1]	48[1]	54[1]	60[1]
History and Physical	X	X	X	X	X	X	X	X	X	X
Procto/Sigmoidoscopy[4]	X	X	X	X	X	X	X	X	X	X
MRI Rectum[4]	X	X		X		X		X		X
CT CAP		X		X		X		X		X
CEA levels[2]	X	X	X	X	X	X	X	X	X	X
QoL[3]	X	X		X		X				

[1] +/- 30 days

[2] After 36 months, CEA will be evaluated every 6 months up to five years, based on NCCN guidelines. MSKCC Only: one to two additional 10 ml EDTA tube(s) of blood will be collected at these time points for research purposes.

[3] Patients may be contacted by phone for follow-up QoL surveys. The QoL is required for patients who are fluent in reading and speaking English accrued at US and English-speaking Canadian sites. Sites must administer appendices approved by MSKCC's IRB as indicated by the MSKCC IRB/PB watermark.

[4]Procto/sigmoidoscopy and MRI must be completed at treating institution

11.0 TOXICITIES/SIDE EFFECTS

FOLFOX and 5-FU are FDA-approved, and are used as part of standard treatment in the US for patients with locally advanced rectal cancer. Therefore, all clinicians recruiting study subjects have considerable experience with this regimen. The toxicity of each specific agent is outlined below. The regimen used in this clinical trial also represents the standard care for patients with locally advanced rectal cancer.

11.1 Anticipated Toxicities – Oxaliplatin

Likely:
- Inflammation of the nerves, resulting in numbness, tingling, etc.
- Numbness and tingling when touching items that are cold (neuropathy)
- Loss of strength and energy
- Nausea
- Decreased production of red blood cells, possibly requiring transfusion

Less Likely:
- Diarrhea
- Loss of appetite
- Fever
- Coughing
- Vomiting
- Pain
- Back pain
- Constipation
- Headache
- Dizziness
- Difficulty sleeping
- Abdominal pain
- Inflammation of the mouth
- Feeling of tightness in the chest
- Fluid retention and swelling
- Reactions at injection site
- Runny nose and watery eyes
- Bone and joint pain
- Upper respiratory infection
- Abnormal limb stiffness
- Decreased production of white blood cells, possibly causing Infection
- Decreased number of platelets, possibly causing bleeding
- Elevated liver function tests

Rare, but serious:
- Blood clots
- Dehydration
- Nose bleeds

- Rash
- Allergic reaction
- Chest pain
- Low blood potassium
- Acid reflux
- Changes in taste perception
- Redness and pain in the skin of the hands and feet (hand-foot syndrome)
- Reddening of the face
- Inflammation of the throat
- Hair loss (reversible)
- Difficulty urinating
- Increased tearing (eyes)

The Sanofi scale, developed to grade sensory neuropathies, will be used to evaluate oxaliplatin-associated sensory neuropathies.

Toxicity Scale for the Sensory Neuropathies Associated with Oxaliplatin	
**Grade**	**Symptoms**
Grade 0	None
Grade 1	Paresthesias/dysesthesias* of short duration that resolve and do not interfere with function.
Grade 2	Paresthesias/dysesthesias* interfering with function, but not in activities of daily living (ADL)
Grade 3	Paresthesias/dysesthesias* with pain or with functional impairment that also interfere with ADL.
Grade 4	Persistent paresthesias/dysesthesias* that are disabling or life threatening.
* May be cold-induced.	

Comparison of the Symptoms and Treatment of Pharyngo-Laryngodysesthesias and Platinum Hypersensitivity Reactions		
**Clinical Symptoms**	**Pharyngo-Laryngeal Dysesthesias**	**Platinum Hypersensitivity**
Dyspnea	present	present
Bronchospasm	absent	present
Laryngospasm	absent	present
Anxiety	present	present
O2 saturation	normal	decreased
Difficulty swallowing	present (loss of sensation)	absent
Pruritus	absent	present
Urticaria/rash	absent	present
Cold-induced symptoms	yes	no
BP	normal or increased	normal or decreased
Treatment	anxiolytics, observation in a controlled clinical setting until symptoms abate or at the physician’s discretion	Oxygen, steroid, epinephrine, bronchodilators; fluids and vasopressors, if appropriate

Neutropenia has been reported in 73% of patients receiving oxaliplatin with 5-FU and Leucovorin (44% grade 3 or 4). Grade 3 or 4 thrombocytopenia is reported to occur in 4% of patients receiving the combination.

11.2 Anticipated Toxicities – 5-fluorouracil

Likely:
- Darkening of the skin and nail beds, dry, flaky skin
- Decreased red blood cells (anemia), the oxygen carrying cells, which could make you feel tired
- Decreased white blood cells (leukopenia), the infection-fighting cells, which could put you at risk for infection
- Nausea
- Vomiting
- Sores in mouth or on lips
- Thinning hair
- Diarrhea
- Brittle nails
- Increased sensitivity to sun

Less likely:
- Darkening and stiffening of the vein used for giving the drug
- Headache
- Weakness
- Muscle aches
- Heartburn (for which you could be given other medication)
- Nausea and vomiting (for which you could be given other medication)
- Loss of appetite
- Hair loss, which is expected to be temporary. Hair is expected to grow back after chemotherapy is stopped.
- Skin irritation, itchy skin rash (for which you could be given skin medication)
- Hand-foot syndrome--characterized by numbness, tingling and painful blistering on the palms of hands or soles of the feet. Usually temporary, but some may be long-lasting. These may be made worse by exposure to cold temperature and cold objects. These effects can be minimized by avoiding exposure to cold (including not drinking cold beverages) for up to 5 days after your infusion.)

Rare but serious:
- Difficulty walking
- Irritation of the eyes
- Increased tearing of eyes
- Blurred vision
- Low blood pressure (for which you could be given other medication)
- Chest pain (You will be evaluated by your doctor to determine the cause. If angina (chest pain) is presented it can be temporary with treatment, but can in the short term lead to a life-threatening heart attack)
- Changes in tests that measure heart rhythms (Temporary side effect; observed and use heart medications for control)
- Decrease in the bone marrow function. Low white blood counts increase the risk of infection and might require antibiotics and possibly hospitalization. Low red blood counts (anemia) would make you feel tired and might require transfusions. Low platelet counts increase the risk of bruising and bleeding and might require transfusions.

11.3 Anticipated Toxicities – Leucovorin

Side effects are uncommon, and are associated with prolonged use. These include an allergic reaction, with symptoms such as skin rash, hives, and itching, and/or breathing problems such as wheezing. Medicines are available to help you if these should occur. Although these side effects are extremely rare, anaphylactic reactions can be serious and are potentially fatal. Leucovorin may interfere with the effects of anti-seizure medications such as phenobarbital, phenytoin, and primidone. When leucovorin is given together with 5-FU, side effects may increase.

11.4 Anticipated toxicities - Capecitabine (Xeloda®)

Likely:
- Diarrhea or loose stools
- Inflammation and/or painful sores in the mouth that may make swallowing difficult (mucositis)
- Nausea
- Redness or sores on palms of the hands or soles of the feet (palmar-plantar erysdysthesia, or “hand-foot syndrome”)
- Dry skin (xerosis)
- Itching sensation (pruritus)

Less likely:
- Decreased red blood cells (anemia), the oxygen carrying cells, which could make you feel tired
- Decreased white blood cells (leukopenia), the infection-fighting cells, which could put you at risk for infection
- Decreased number of platelets (thrombocytopenia), the blood-clotting cells, which could put you at increased risk of bleeding
- Vomiting
- Stomach or abdominal pain
- Loss of appetite, not eating (anorexia)
- Constipation
- Heartburn
- Fatigue
- Generalized weakness and loss of strength
- Hair loss
- Rash
- Red, sore eyes
- Fever
- Sensation of light-headedness or vertigo (dizziness or spinning sensation)
- Headache
- Pain, including joint, muscle, or bone pain
- Infection

Rare but serious:
- Blood clots and/or bleeding
- Dehydration
- Abnormal liver function tests
- Heart attack
- Abnormal heartbeat

11.5 Anticipated Toxicity – Radiotherapy

Expected acute side effects of radiation therapy include:
- Abdominal cramping
- Diarrhea
- Skin erythema
- Dysuria
- Tenesmus
- Leukopenia
- Lymphopenia
- Thrombocytopenia

Late effects of radiation therapy include:
- Increased frequency of bowel movements
- Bowel urgency
- Infertility in women who have not undergone an ovarian transposition
- Early menopause in pre-menopausal women who have not undergone an ovarian transposition
- Vaginal dryness and narrowing

11.6 Surgical Complications

Surgery is part of the standard treatment of most patients with rectal cancer. The most common complications of surgery for rectal cancer include wound infection, pelvic infection, bleeding, bowel obstruction, vein thrombosis, pneumonia, cardiac arrhythmia, and heart attack. Long-term side effects include frequent bowel movements, urinary problems, sexual dysfunction, and hernia. Surgical complications will be graded by the treating surgeon according to the Clavien-Dindo classification of surgical complications. [88] Refer to Appendix N.

11.7 Dosage Modifications Based on Adverse Events

11.7.1 Dose Modifications for Radiotherapy

Uninterrupted treatment is planned. Treatment may be interrupted for acute toxicity. Radiation therapy will be held on any planned treatment day in which the patient exhibits Grade 3 toxicity, unless such toxicity cannot be reasonably attributed to pelvic radiation (i.e., stomatitis or hand-foot syndrome). Radiation will be held until the toxicity resolves to less than Grade 2, at which point radiation will be resumed. If radiation treatment is held, then chemotherapy should also be held. A toxicity which delays planned radiation therapy for greater than 2 weeks will be adjudicated as dose-limiting toxicity for the purposes of this trial. The patient will be examined at least once a week, or as indicated by the treating physician, during the course of radiation. CBC and platelets will be drawn weekly or as indicated at the discretion of the treating physician. RT interruption is to be minimized, and is allowed only for regional symptoms.

11.7.2 Dose modification for FOLFOX, CapeOX, and 5-FU and Capecitabine during radiation

FOLFOX, CapeOX, and 5-FU and capecitabine during radiation are standard regimens that have been widely used for the treatment of colon and rectal cancer for more than a decade. Investigators should refer to package and inserts and local pharmacy practices for a

complete list of potential toxicities, and adhere to best practices. If chemotherapy is held during radiation, radiation may be continued at physician discretion.

Treatment interruptions and modifications should be made at the discretion of the treating physician. The following dose reduction and modification tables should be used as a guide. If the treating physician feels that it is feasible to continue treatment without interruption, and/or use a modification other than what is indicated below, and without putting the patient at undue risk, then the treating physician may proceed with treatment as planned.

Dose Levels of sensitizing chemotherapy during radiation are as follows:

**Dose Level[1]**	**5-FU Infusion[2]**	**Capecitabine**
0	225 mg/m^2/day	1650 mg/m^2
-1	175 mg/m^2/day	1237 mg/m^2
-2	135 mg/m^2/day	825 mg/m^2
-3	100 mg/m^2/day	650 mg/m^2

1 Dose level 0 refers to the starting dose

2 There no dose level restrictions for either infusional 5-FU or capecitabine during chemoradiation. Patients will remain active in the study and on schedule irrespective of any dose modifications to 5-FU and capecitabine made by the treating physician.

Dose reductions for Oxaliplatin, Leucovorin, and 5-FU are as follows:

**Drug**	**Dose Level 0 (mg/m^2)**	**Dose Level -1 (mg/m^2)**	**Dose Level -2 (mg/m^2)**	**Dose Level -3 (mg/m^2)**
Oxaliplatin	85	65	50	0
Leucovorin	400	320	260	260
5-FU bolus	400	320	260	260
5-FU Infusion	1200	900	720	720

The following tables describe those modifications to be used for Day 1 of a new cycle of FOLFOX therapy and are suggested guidelines for treatment modifications. All dose modifications in a new cycle are based upon the most severe toxicity observed in the previous cycle. Any modifications to treatment should be made at the discretion of the treating physician.

Dose Modification for Oxaliplatin, 5-FU, and Leucovorin			
**Toxicity: CTCAE grade**	**Oxaliplatin**	**5-FU**	**LV**
**Neutropenia or thrombocytopenia**			
Grade 2	Hold to resolution to ≤grade 1. Resume at same dose.	Hold to resolution to ≤grade 1. Resume at same dose.	Hold to resolution ≤grade 1. Resume at same dose.
Grade 3 or 4	Hold to resolution to ≤grade 1. Resume at dose decreased by 1 level.	Hold to resolution to ≤grade 1. Resume at dose decreased by 1 level.	Hold to resolution to ≤grade 1. Resume at same dose.
**Neutropenic fever or infection**	Hold to resolution to ≤grade 1. Resume at dose decreased by 1 level.	Hold to resolution to ≤grade 1. Resume at dose decreased by 1 level.	Hold to resolution ≤grade 1. Resume at same dose.
**Diarrhea**			
Grade 3 or 4	Hold to resolution to ≤grade 2. Resume at dose decreased by 1 level.	Hold to resolution to ≤grade 2. Resume at dose decreased by 1 level.	Hold to resolution to ≤grade 2. Resume at same dose.
**Mucositis**			
Grade 2	No modification	No modification	No modification
Grade 3	No modification	Hold to resolution to ≤grade 2. Resume at dose decreased by 1 level.	Hold to resolution to ≤grade 2. Resume at dose decreased by 1 level.
Grade 4	Hold to resolution to ≤grade 2. Resume at dose decreased by 1 level.	Hold to resolution to ≤grade 2. Resume at dose decreased by 1 level.	Hold to resolution to ≤grade 2. Resume at dose decreased by 1 level.
**Vomiting**			
Grade 0-2	No modification	No modification	No modification
Grade 3	Hold to resolution to ≤grade 2. Resume at dose decreased by 1 level.	Hold to resolution to ≤grade 2. Resume at same dose.	Hold to resolution to ≤grade 2. Resume at same dose.
Grade 4	Hold to resolution to ≤grade 2. Resume at dose decreased by 1 level.	Hold to resolution to ≤grade 2. Resume at dose decreased by 1 level.	Hold to resolution to ≤grade 2. Resume at same dose.
**Pulmonary**			
Cough, dyspnea, hypoxia, pneumonitis or pulmonary infiltrates ≥grade 3	Hold oxaliplatin until interstitial lung disease is ruled out. Resume at same dose.	No modification	No modification
**Other non-hematologic toxicities**			
Grade 0-2	No modification	No modification	No modification

Grade 3 or 4	Hold to resolution to ≤grade 2. Resume at dose decreased by 1 level.	Hold to resolution to ≤grade 2. Resume at dose decreased by 1 level.	Hold to resolution to ≤grade 2. Resume at same dose.

Dose reductions for Oxaliplatin and Capecitabine are as follows:

Drug	Dose Level 0[1] (mg/m^2)	Dose Level -1 (mg/m^2)	Dose Level -2 (mg/m^2)	Dose Level -3 (mg/m^2)
Oxaliplatin	130	100	80	65
Capecitabine	1000	800	640	510

1 Dose Level 0 refers to the starting dose.

The above table describes those modifications to be used for Day 1 of a new cycle of CapeOx therapy and are suggested guidelines for treatment modifications. All dose modifications in a new cycle are based upon the most severe toxicity observed in the previous cycle. Any modifications to treatment should be made at the discretion of the treating physician.

11.7.2.1 Retreatment Criteria for 5-fluorouracil, Leucovorin, and Oxaliplatin

Subsequent to dose delays, patients may be re-treated with chemotherapy provided that all grade 3-4 toxicities have resolved to < grade 1.

11.7.2.2 Dose modifications of Leucovorin

There are no dose modifications for Leucovorin. If the 5-FU is skipped (bolus and infusion), then Leucovorin should also be held. If Leucovorin is not available, it is at the discretion of the treating physician to administer FOLFOX without Leucovorin or to use Fusilev (Levoleucovorin) as an alternative to Leucovorin. If Fusilev (Levoleucovorin) is used as a substitute for Leucovorin, the patient should receive a dose of 50% of the protocol-specific dose of racemic Leucovorin. In keeping with the protocol-specific Leucovorin dose of 400 mg/m^2, the Fusilev equivalent is 200 mg/m^2.

11.7.3 Dose modification for Hand-Foot Syndrome (HFS)

The Common Terminology Criteria for Adverse Events (CTCAE) version 4.0 will be used. Grade 1 HFS is defined as numbness, dysesthesia/paresthesia, tingling, painless swelling or erythema of the hands and/or feet, and/or discomfort which does not disrupt normal activities of daily living. Grade 2 HFS is defined as painful erythema and swelling of the hands and/or feet and/or discomfort affecting the patient’s normal activities of daily living. Grade 3 HFS is defined as moist desquamation, ulceration, blistering and severe pain of the hands and/or feet and/or discomfort that causes the patient to be unable to work or perform normal activities of daily living. If Grade 2 or 3 HFS occurs, administration of capecitabine should be interrupted until the event resolves or decreases to Grade 1. Subsequent doses of capecitabine should be decreased.

11.8 Supportive Therapies

11.8.1 Antidiarrheals

For symptoms of diarrhea and/or abdominal cramping that occur at any time during a treatment cycle with 5-FU, patients will be instructed to begin taking antidiarrheal medications at the physician discretion. Additional antidiarrheal measures may be used at the discretion of the treating physician. Patients should be instructed to increase fluid intake to help maintain fluid and electrolyte balance during episodes of diarrhea.

11.8.2 Hematopoietic Growth Factors

Use of granulocyte colony-stimulating factor (G-CSF, filgrastim, Neupogen®) is permitted at investigator discretion. The use of erythropoietin and its analogues, such as darbepoetin, is also permitted at the discretion of the treating physician. ASCO guidelines for the use of colony-stimulating factors should be consulted.

11.8.3 Other Concomitant Medications

Therapies considered necessary for the patient's well-being may be given at the discretion of the investigator. Other concomitant medications should be avoided except for analgesics, treatments for chronic medical conditions, or agents required for life-threatening medical problems. If possible, the use of drugs with laxative properties should be avoided because of the potential for exacerbation of diarrhea. Patients should be advised to contact the physician to discuss any laxative use. International normalized ratio (INR) elevations, bleeding, or both have been reported in some patients taking warfarin concurrently with 5-fluorouracil-containing chemotherapy. Patients taking warfarin should be monitored regularly for changes in prothrombin time (PT) or INR. Concomitant use of medications known to affect the conductive system, such as beta-blockers, calcium channel blockers, or digoxin, is allowed under investigator supervision.

12.0 CRITERIA FOR THERAPEUTIC RESPONSE/OUTCOME ASSESSMENT

The primary objective of this study, 3-year DFS, will be assessed on the basis of surveillance procto/sigmoidoscopy, physical examination, and follow-up radiologic scans. For patients who receive TME or NOM, follow-up scans will occur according to current NCCN guidelines for rectal cancer surveillance. Patients in the NOM group will be evaluated using rectal MRI and endoscopic exam (section 10). Patients in the NOM group who undergo subsequent TME will be followed according to current NCCN guidelines. 3-year DFS will be defined as the percentage of patients alive without recurrence of disease at 3 years measured from the date of randomization. Patients in the NOM protocol who develop relapse at the site of the primary tumor but undergo curative TME with negative resection margins will not be counted as having recurrence. However, any patients developing local recurrence after TME, performed either immediately after neoadjuvant therapy or after a period of NOM, will be counted as having recurrence. Scans will be reviewed at MSKCC to resolve any discrepancies in interpretation. Scans showing equivocal evidence of local recurrence will be evaluated with additional imaging as necessary. Biopsy confirmation of local recurrence will be indicated for the purposes of the protocol. Isolated elevation of CEA, in the absence of other evidence of recurrent disease, will not be considered indicative of either local or systemic relapse. Symptomatic evidence of local recurrence will be corroborated on the basis of imaging and/or physical examination. Distant recurrence will also be assessed using follow-up CT scans of the chest, abdomen and pelvis. The MSKCC radiologist will review scans to resolve any discrepancies in interpretation. Central radiology review at MSKCC will review all incoming images and reports, but will only submit feedback on cases in which scans must be repeated or, in the case of interval or re-staging evaluations, where progression is identified in disagreement with the site investigator’s assessment. Scans with equivocal evidence of distant recurrence will be evaluated via additional imaging, as

necessary. Biopsy confirmation of distant recurrence will be indicated for the purposes of the protocol.

Adverse events associated with CT and RT-CT will be assessed using the standard criteria described in section 11.0.

Surgical complications following TME will be assessed and recorded by the treating physician according to the Clavien-Dindo classification of surgical complications. [88] Refer to Appendix N to see the full scale.

13.0 CRITERIA FOR REMOVAL FROM STUDY

All patients will be followed for five years from the last date of treatment. Only patients that choose to withdraw consent will be removed from the study and will no longer be followed.

A patient has the right to withdraw from the study at any time for any reason without prejudice to his/her future medical care by the physician or at the institution.

Every effort will be made to evaluate patients for eligibility prior to enrollment. However, if it is discovered that a patient is found to be ineligible after enrollment, the patient will be removed from the study.

Patients may discontinue protocol treatment without being taken off study. Patients who do not complete protocol treatment will remain on study but will be treated at the discretion of their treating physician. Patients placed on the NOM arm who then undergo TME at a later date will remain on study and be followed according to current NCCN guidelines.

If a patient chooses to discontinue treatment, is removed for clinical reasons, and/or received non-protocol treatment, the study team will continue to follow the patient based on intent-to-treat and collect data for up to five years.

Patients may discontinue treatment for any of the following reasons:

1. Unacceptable toxicity, both severe and unexpected
2. Investigator considers it would be in the patient’s best interest to not continue
3. Patient is unwilling or unable to continue study treatment
4. Patient becomes pregnant
5. Lost to follow-up

Patients that discontinue protocol treatment must be followed for existing adverse events (AEs) for 30 calendar days after the last day of study treatment. All Grade ≥3 events considered unexpected and SAEs (as outlined in 17.2) occurring during that period must be reported to the participating site IRB and MSK per section 17.2. These events must be followed up until resolved, unless the condition is unlikely to resolve because of underlying disease, in the treating physician’s opinion. After the 30-day period, patients will be followed for disease and survival status for up to 5 years. Limited information about subsequent treatment will be collected and may include chemotherapy, radiation therapy, and clinical exams, and imaging.

If a patient does not return for a scheduled visit, every effort should be made to contact him/her. In any such circumstance, every effort should be made to document the subject's outcome. Patients who leave MSKCC or the treating site or continue their care elsewhere will be contacted every 6 months by telephone, email, or mail. After three failed attempts to reach the patient, either via mail or telephone or a combination of the two, contact will cease and the patient will be considered lost to follow-up.

In the event of death while on study, death will be recorded as a Grade 5 SAE. If death is due to progression, this will be captured accordingly as death is a possible outcome.

14.0 BIOSTATISTICS

14.1 Primary objective: *To evaluate 3-year DFS survival in patients managed with TNT and TME or NOM, compared with standard historical controls managed according to standard of care (CRT and TME followed by ACT).*

We hypothesize that 85% of patients with LARC treated with TNT and selective NOM will be alive and free of disease at 3 years. The 3-year DFS for similar patients (stage II/III LARC within 6 cm from the anal verge) treated according to the standard of care (CRT, TME, and ACT) is 75%. (Survival data provided by Maas et al from a pooled analysis of 3105 rectal cancer patients treated with CRT and TME). [35] In this trial, each arm is designed as a single-stage study that discriminates between 3-year DFS rates of 75% (historical control) and 85% (Study Arms, INCT and CNCT). No formal statistical comparison of the two arms is planned.

For our power calculation, we assume uniform accrual over time and an exponential distribution for time to death. Based on our previous experience, we can assume that we will be able to accrue 2-4 patients per arm per month. Using the approach proposed by Lawless (1982), for 86% power, a two-sided type I error of 5% we will require 101 patients per arm. Based on our estimated accrual rate, a total of 202 patients can be accrued over 40 months with an additional 3-year follow-up.[95] With a sample size of 101 patients (each arm), we will consider the treatment regimen of an arm worthy of further study if the 3-year DFS rate exceeds (based on parametric exponential estimate) .82 (the upper critical value). The 3-year rates will be estimated using parametric exponential estimates as well as on Kaplan-Meier estimates. Analysis of the primary endpoint will occur 3 years following the completion of accrual. We anticipate about 10% loss to follow-up (dropouts that occur before the entire regimen has been completed), and will recruit an additional 10 patients per arm to account for this. Patient accrual for the entire trial is expected to take 40 months, with approximately 6 patients (2-4 per arm) per month. We will update the analysis after we have completed follow-up. Accrual has been completed a year ahead of schedule. Preliminary results indicate that we may require additional patients to detect differences in organ preservation rates, one of the secondary aims of this study. Based on this new information, we plan to increase accrual to 325 patients. As accrual has been progressing faster than initially estimated, we expect that, with the proposed increase, accrual will still be completed within the initially proposed accrual period.

This trial could complete with 3 possible results: both arms are worthy of further study, one arm is worthy of further study, no arm is worthy of further design. In all three cases, all Aim 2 analyses will be performed. In the ambiguous case when both the INCT and CNCT arms are worthy of further study, we will use a pick-the-winner strategy based on the number of NOM patients. This is described in more detail in section 14.2.

14.2 Secondary objective 1.: *To compare outcomes in patients treated on the two arms of this study, with respect to rates of organ preservation, compliance with the neoadjuvant protocol, and major adverse events.*

Based on previous data, we anticipate a 20% NOM in the INCT arm, and 35% in the CNCT arm, after 3 years. The proportion of patients initially entered in the NOM protocol will be higher, but some will later relapse and require TME. For this reason, we anticipate that the proportion of patients with NOM at 3 years will be close to the pCR rates observed in previous studies. We estimated that the 222 patients required for the primary aim would be sufficient to detect differences in organ preservation rates. Our preliminary results indicate that the proportions of patients selected for NOM based on tumor response after completion of neoadjuvant therapy are much greater than expected: 66% in one arm and 79% in the other. Although many of these patients may eventually experience tumor regrowth requiring total mesorectal excision, the proportions of patients with a preserved rectum 3 years after diagnosis are likely to be greater than the original estimates. Therefore, based on the new information, we plan to increase accrual to 325 patients. There is limited information regarding treatment compliance and adverse events in patients who have received more than 4 or 6 cycles of INCT or CTNT. Therefore, these secondary objectives are considered exploratory. If both arms meet the endpoint in primary objective, we plan to use NOM rate to determine the more promising regimen using a “pick the winner” strategy. We will calculate the number of patients treated with NOM who are alive and free of disease 3 years following the start of the study. We will require at least 20 NOM patients in each arm to employ the following strategy: If there is a difference of 5 NOM patients between arms, the arm with more NOM patients will be deemed the winner. With 101 patients in each arm, 20% NOM in patients treated with INCT and 30% in patients treated with CNCT, we will have an 83% probability of selecting CNCT, a 1% probability of selecting INCT, and a 16% probability of considering the study inconclusive.

In addition, we will calculate therapy compliance using the following measures: the number of days RT was held, the number of RT delays of $\geq$ 1 week, the number of dose delays and the number of dose reductions in INCT and CNCT. We will also calculate the rate of select hematologic events (as outlined in 17.2), select non-hematologic events (as outlined in 16.0), and unexpected Grade 3 or higher non-hematologic adverse events and surgical complications in each treatment arm. Comparisons will be done using the Wilcoxon Rank Sum test or the Fisher’s Exact test.

14.3 Secondary objective 2.: *To measure patient-reported functional outcomes and QoL in patients with LARC treated with NCT, CRT and NOM, and compare these to patients treated with TME.*

The primary analyses for this aim will use data combined across both treatment arms; however, differences according to treatment will also be investigated. The primary endpoint will be QoL as measured one year from the end of TNT. Secondary endpoints will be bowel, sexual, and bladder function. While the subscales will be reported, the primary secondary endpoints will be the total scores on the BFI, FSFI and IIEF. With a total of 325 patients accrued, we expect that 129 patients will obtain complete clinical response and 196 will undergo TME. Approximately 15 to 30 patients initially treated with NOM are expected to cross over to TME at some point during follow-up. For the primary analyses, the NOM group will include these cross-overs on an intent-to-treat analysis. In secondary analysis, patients who eventually crossed over to receive surgery will be included in the TME group. We define this analysis as a comparison between TME vs. durable NOM. Durable NOM is defined as 3-year disease-free survival. We expect that 70% of patients will complete the QoL assessment at 1 year. This response rate takes into account refusals to complete the QoL

questionnaires, and the approximately 20% of patients who will develop distant disease or die before the 1-year time point. Thus, at 1 year we expect to have information on 94 TME patients and 62 NOM patients.

The primary endpoint of QoL will be assessed using the EQ-5D index, an overall measure of QoL and health ranging from 0 (worst health) to 1 (best health). Comparison will be done using the two-sample t-test. The minimally important difference (MID) for 534 cancer patients in the United States has been estimated to be in the range of .05 to .09 when using ECOG performance status as an anchor (Pickard, Neary, Cella 2007). The standard deviation of the EQ-index was .15 in this patient population. With our sample size of 156, we will be able to detect a difference of .07 with 80% power and type I error of 5%, assuming a standard deviation of .15.

In secondary analyses we will use a t-test to compare the EQ-5D index, measured at 1 year, in patients with durable NOM vs. TME. The differences in EQ-5D index from 1 year to 3 years will also be compared in patients with durable NOM vs TME, using an ANCOVA model that adjusts for variables found to be significantly different by NOM vs TME. Trends in the EQ-5D from baseline, 12, 24, and 36 months will be graphed, and evaluated descriptively.

Similar analyses will be done to study all other patient-reported functional and QoL endpoint.

14.4 Correlative studies: objectives

The correlatives studies will be conducted in patients accrued at MSKCC only. During the past five years, an average of 120 patients with stage II and III rectal cancer have been treated at MSKCC; 43 of them had tumors located within 6 cm from the anal verge, and are potentially eligible for the trial. We estimate that 20 patients (less than 50% of eligible patients) will be accrued to the trial every year, for a total of 80 patients over the 4-year accrual period. Assuming equal distribution between arms, we anticipate that 56 patients will ultimately be treated with TME and 24 with NOM.

Endpoints for each of the aims will be compared between patients treated with TME and patients treated with NOM. Endpoints for each of the aims are as follows:

14.4.1 To investigate the diagnostic performance of conventional and diffusion-weighted magnetic resonance imaging (DW-MRI) in identifying patients with LARC treated with TNT, who may benefit from NOM.

Measurements used for correlation include mrTRG in T2-w imaging, assessment of DW signal intensity (SI), mrΔvolume between baseline and post-TNT examinations in both T2-w and DW imaging, and changes in ADC, true diffusion (D), and perfusion fraction (f) between baseline and post-TNT DW-MRI. The outcome will be the proportion of patients treated successfully with NOM, meaning the patient is free of disease at the 3-year endpoint, plus patients with ypT0 after TME.

This will be the first study investigating the diagnostic performance of conventional and DW-MRI in LARC patients treated with TNT and NOM. Therefore, this aim will be considered exploratory.

DW-MRI will be done for all MSKCC patients. Participating sites may perform DW-MRI, but this is not required. If one exam for a patient is performed as DW-MRI, all subsequent exams for the patient should be done using the same technique. If performed, DW-MRI will be captured in the appropriate case report forms.

14.4.2 To evaluate the feasibility of using circulating tumor DNA and miRNA profiles in plasma to monitor tumor response to TNT in rectal cancer patients treated in both protocol arms.

The measurement will be the presence of tumor specific mutations—defined as mutations present in the pretreatment biopsy— in plasma DNA of patients in both study groups at different time points (see Section 10.0). A second measurement for this aim will be the plasma miRNA profiles as the same time points as above. We will graphically examine these two measurements in patients treated successfully with NOM plus patients with ypT0 after TME and with patients who had residual tumor after TME.

As this will be the first prospective study investigating the circulating tumor DNA and miRNA profiles in rectal cancer patients treated with TNT and NOM, this aim will be considered exploratory.

Patients who take part in this portion of the study will be from Memorial Sloan Kettering only. Given the smaller sample size, all analyses will be descriptive. We will graph each measurement over time for the two groups defined above. If possible, comparisons at each time point will be done using nonparametric methods, such as the Wilcoxon rank-sum test.

14.4.3 Use of genomic analysis by next generation sequencing to profile rectal cancer treated with neoadjuvant chemotherapy and radiation.

The measurements for this study will be the mutation profile, copy number alteration profile, and gene expression profile in pretreatment tumor biopsies of patients treated in both study arms. We will compare the results in patients treated successfully with NOM plus patients with ypT0 after TME, with patients who had residual tumor after TME.

As this will be the first prospective study investigating the genomic profile of rectal cancer patients treated with TNT and NOM, this aim will be considered exploratory.

14.4.4 Investigation of the molecular mechanisms of tumor resistance to neoadjuvant therapy by genomic analysis of rectal cancer before and after treatment.

The endpoint for this study will be the proportion of cells expressing the colonic stem cell biomarkers in pretreatment tumor biopsies of patients treated in both groups. We will compare the results in patients treated successfully with NOM plus patients with ypT0 after TME and compare them with patients who had residual tumor after TME. In addition we will investigate the expression of colonic stem cell biomarkers in the surgical specimens of patients with residual tumor after TME.

14.5 Stopping rules

To ensure patient safety, we have introduced a stopping rule for R1 resections among NOM patients requiring TME. The rate of R1 resection after TME in similar patient populations ranges from 5% to 10%. For our trial, we consider an R1 resection rate of 10% acceptable, and 25% unacceptable. Continuous monitoring based on repeated significance testing will be implemented.[96] We will stop the trial if the number of R1 resections in the NOM group requiring TME exceeds the numbers listed below. Based on these rules, the probability of crossing the boundary is 10% if the true toxicity rate is 10% and is 99% if the true toxicity rate is 25%.

	Patients in NOM who require TME	2	6	11	17	23	29	36	43	50	57	64	71	79
	R1 Resection (positive margins)	1	2	3	4	5	6	7	8	9	10	11	12	13

15.0 RESEARCH PARTICIPANT REGISTRATION AND RANDOMIZATION PROCEDURES

15.1 Research Participant Registration

MSKCC only

Confirm eligibility as defined in the section entitled Criteria for Patient/Subject Eligibility.

Obtain informed consent, by following procedures defined in section entitled Informed Consent Procedures.

During the registration process registering individuals will be required to complete a protocol specific Eligibility Checklist. The individual signing the Eligibility Checklist is confirming whether or not the participant is eligible to enroll in the study. Study staff are responsible for ensuring that all institutional requirements necessary to enroll a participant to the study have been completed. See related Clinical Research Policy and Procedure #401 (Protocol Participant Registration).

15.1.1 For Participating Sites:

Central registration for this study will take place at MSKCC.

To complete registration and enroll a participant from another institution, the study staff at that site must contact the designated research staff at MSKCC to notify him/her of the participant registration. The site staff then needs to fax registration/eligibility documents to the Department of Surgery at MSKCC: 646-227-7267, Attn: MSKCC Study Coordinator. If scanned copies are sent, they must be sent using secured or password-protected e-mail. Scanned copies can be e-mailed to the MSKCC Study Coordinator ski13213@mskcc.org. Faxed submissions should include a cover page listing all documents included per participant.

The following documents must be sent for each enrollment within 24 hours of the informed consent form being signed:

- The completed or partially completed MSKCC Eligibility Checklist
- The signed Informed Consent and HIPAA Authorization form
- Supporting source documentation for eligibility questions (laboratory results, pathology report, radiology reports, endoscopic reports, MD notes, physical exam sheets, medical history, prior treatment records, and EKG report).

Upon receipt, the research staff at Memorial Sloan Kettering Cancer Center will conduct an interim review of all documents and will associate the participant to the study in MSK's Clinical Trials Management System (CTMS). The participant will be assigned a protocol participant number in CTMS. This number will be relayed back to the study staff at the registering participating site via e-mail and will serve as enrollment confirmation. The number is unique and must be written on all data and correspondence for the participant.

If the eligibility checklist is not complete or source documentation is missing, the participating site will be responsible for sending the completed registration documents within 30 days of the consent. Once the external registration submission is complete, if the participating site IRB has granted approval for the protocol and the participating site is in good standing, the MSK study coordinator will fully register the participant in CTMS. The participating site will be notified by the MSK study coordinator when registration is complete.

15.2 Randomization

This is a randomized phase II study with Arm 1 receiving INCT prior to CRT while Arm 2 receives CRT followed by CNCT. After eligibility is established and after consent is obtained, patients will be registered in the Protocol Participant Registration (PPR) system, and randomized using the Clinical Research Database (CRDBi-Multicenter). If a patient is pending registration based on limited available information, randomization will occur after all completed documentations is received. A patient may be registered as pending for up to 30 days from the initial registration submission to PPR. Randomization will be stratified per institution and accomplished by the method of random permuted block. Since this is not a blinded study, all study investigators may view randomization in the CRDBi-Multicenter.

For participating institutions other than MSKCC, randomization will be stratified per institution and determined using the same method for MSKCC patients as noted above. Confirmation of the treatment plan for randomized participants will be sent by the MSKCC research staff via e-mail, along with the participant ID, to the study coordinator and the site Principal Investigator within 48 business hours of receiving all completed required enrollment documents.

16.0 DATA MANAGEMENT ISSUES

A Clinical Research Coordinator(CRC) will be assigned to the study at each site. The responsibilities of the CRC include project compliance, data collection, abstraction and entry, data reporting, regulatory monitoring, problem resolution and prioritization, and coordination of the activities of the protocol study team. The data collected for this study will be entered into a secured database (Clinical Research Database, CRDBi-Multicenter) at MSKCC. Source documentation will be available to support the computerized patient record. Electronic files with copies of the informed consent, eligibility checklists, adverse events, and outcomes assessments will be kept in a secure institutional network. The study CRC, Clinical Research Supervisor, and the supervising Clinical Research Manager will also have access to these files.

Data collection will principally assess:

- Adherence to eligibility criteria: The study CRC will ensure that all eligibility criteria are met and that the checklist is complete and signed by the consenting professional, prior to initiation of treatment. All supporting source documentation must be maintained in the patient's research file.
- Safety Evaluation: Adverse events (including hematologic and non-hematologic toxicities) will be recorded as they occur, and graded according to the Cancer

Therapy Evaluation Program Common Terminology Criteria for Adverse Events (CTCAE) Version 4.0 (http://ctep.info.nih.gov). Toxicities that cannot be graded using the NCI CTCAE v4.0 will be graded as grade 1 (asymptomatic), grade 2 (symptomatic but not interfering significantly with function), grade 3 (causing significant interference with function), grade 4 (life-threatening), or grade 5 (death). For study purposes, reportable non-hematologic events will be limited to Grade ≥3 diarrhea, Grade ≥3 neuropathy, Grade ≥3 DVT, and those events that are Grade ≥3 and considered unexpected. Reportable hematologic events will be limited to Grade ≥ 4 neutropenia/decreased neutrophil count, Grade ≥ 3 thrombocytopenia, Grade ≥ 3 neutropenic fever (with fever greater than 38.2°C) (outlined in section 17.2). Post-operative complications will be graded according to the Clavien-Dindo classification (see Appendix N).

16.0.1 Data Entry and Source Documentation Requirements for Participating Sites

Data Entry

The participating site(s) will enter data remotely into MSKCC's internet-based Clinical Research Database, termed CRDBi -Multicenter. In the event that there are problems using this system, the MSKCC research team should be contacted directly. The site staff will receive CRDBi-Multicenter training prior to enrolling its first patient, and a data entry manual will be provided. The participating site PI is responsible for ensuring that data provided to MSKCC are complete, accurate and provided in a timely manner. A schedule of required forms is show in section 16.0.2

Source Documentation

Source documentation refers to original records of observations, clinical findings and evaluations that are subsequently recorded as data. Source documentation should be consistent with data entered into CRDBi-Multicenter. Relevant source documentation to be submitted throughout the study includes:

- Baseline MRI images and report to confirm staging (see Section 9.3.4.2)
- Baseline pathology report to confirm diagnosis
- Chemotherapy flow sheets/pill diaries from both groups (induction and consolidation) and from each treatment arm (chemoradiation and neoadjuvant chemotherapy) (see section 9.1.1, 9.2.6.1)
- Radiation simulation films and treatment reports (see Section 9.2.5)
- Grade 3-5 toxicities/adverse events (see section 16.0, 17.2 for list of reportable events)
- Proctoscopy/sigmoidoscopy reports and images (when clinically feasible), and MRI images and reports
- Surgery report (if applicable)
- Pathology report (if applicable)

Source documentation should include a minimum of two identifiers to allow for data verification. MSK will maintain the confidentiality of any subject-identifiable information it may encounter.

Source documentation should be sent as requested to MSKCC to the contact provided below. Please note that source documents may be faxed, but scanned copies are preferred

to help expedite review. If scanned copies are sent, they must be sent using secured or password-protected e-mail. Faxed submissions should include a cover page listing all documents included per participant.

FAX: 646-227-7267, to the attention of MSKCC Study Coordinator
EMAIL: ski13213@mskcc.org, to the attention of MSKCC Study Coordinator

For urgent treatment specific questions or time-sensitive matters, email the central study address ski13213@mskcc.org.

16.0.2 Data and Source Documentation Submission Timelines for Participating Sites

Data and source documentation should be transmitted to MSKCC according to the Submission Schedule below. For questions regarding data entry, please contact the MSKCC Study Coordinator at ski13213@mskcc.org. See the CRDBi manual for detailed data entry instructions.

Time points	Baseline	Treatment Phase	Off Treatment	Long Term Follow up	SAE	Off Study
**Submission Schedule**						
**Source Documentation**	Within 24 hours (see section 15.1.1)	Within 14 days of visit or treatment end date			Within 3 business days of learning of the event(see section 17.2); updates to be submitted as available	Within 14 days of visit
**eCRFs**	Within 7 days of visit					
**Required Forms**						
Patient Information	X					
Minimal Dataset	X	X	X	X		X
Physical Exam	X					
Laboratory	X	X	X			
Treatment		X				
External Beam Radiation		X				
Questionnaires	X	X	X	X		
Toxicity[1]		X	X			X
Comorbidity	X					

Serious Adverse Events		X	X		X	

[1]Adverse events should be entered into the CRDB toxicity form as applicable during the treatment. Refer to section 16.0 for reportable non hematologic events and 17.2 for reportable hematologic events.

16.1 Quality Assurance

16.1 Quality Assurance for MSKCC

Completeness of registration data will be monitored by the CRC on a regular basis. Routine data quality reports will be generated to assess missing data and inconsistencies. Accrual rates, and extent and accuracy of evaluations and follow-up, will be monitored periodically throughout the study period, and potential problems will be brought to the attention of the study team for discussion and action. Random-sample data quality and protocol compliance audits will be conducted by the study team at a minimum of twice per year, or more frequently if indicated.

16.1.1 Quality Assurance for Participating Sites

Each site accruing participants to this protocol will be audited by the staff of the MSK study team for protocol and regulatory compliance, data verification and source documentation.

Audits will be conducted annually during the study (or more frequently if indicated) and at the end or closeout of the trial. Ideally the first audit will occur shortly after the first patients are enrolled. The number of participants audited will be determined by auditor availability and the complexity of the protocol. Each audit will be summarized and a final report will be sent to the PI at the audited participating site within 30 days of the audit.

16.1.2 Response Review

Since therapeutic efficacy is a stated primary objective, all sites participants' responses are subject to review by MSK's Therapeutic Response Review Committee (TRRC). Radiology, additional lab reports and possibly bone marrow biopsies and/or aspirates will need to be obtained from the participating sites for MSK TRRC review and confirmation of response assessment. These materials must be sent to MSK promptly upon request.

16.2 Data and Safety Monitoring

The Data and Safety Monitoring Plan utilized for this study must align with the MSK DSM Plan where applicable.

The Data and Safety Monitoring (DSM) Plans at Memorial Sloan Kettering were approved by the National Cancer Institute in August 2018. The plans address the new policies set forth by the NCI in the document entitled "Policy of the National Cancer Institute for Data and Safety Monitoring of Clinical Trials."

There are several different mechanisms by which clinical studies are monitored for data safety and quality. At a departmental/PI level, there exist procedures for quality control by the research team(s). Institutional processes in place for quality assurance include protocol monitoring, compliance and data verification audits, staff education on clinical research QA, and two institutional committees that

are responsible for monitoring the activities of our clinical trials programs. The committees: Data and Safety Monitoring Committee (DSMC) for Phase I and II clinical trials, and the Data and Safety Monitoring Board (DSMB) for Phase III clinical trials, report to the Deputy Physician-in-Chief of Clinical Research.

The degree of monitoring required will be determined based on level of risk and documented.

The MSK DSMB monitors phase III trials and the DSMC monitors non-phase III trials. The DSMB/C have oversight over the following trials:

- MSK Investigator-Initiated Trials (IITs; MSK as sponsor)
- External studies where MSK is the data coordinating center
- Low risk studies identified as requiring DSMB/C review

The DSMC will initiate review following the enrollment of the first participant, or by the end of the year one if no accruals, and will continue for the study lifecycle until there are no participants under active therapy and the protocol has closed to accrual. The DSMB will initiate review once the protocol is open to accrual.

16.3 Regulatory Documentation

Prior to implementing this protocol at MSKCC, the protocol, informed consent form, HIPAA authorization and any other information pertaining to participants must be approved by the MSKCC Institutional Review Board/Privacy Board (IRB/PB). Prior to implementing this protocol at the participating sites, approval for the MSKCC IRB/PB approved protocol must be obtained from the participating site's IRB.

The following documents must be provided to MSKCC before the participating site can be initiated and begin enrolling participants:

- Participating Site IRB approval(s) for the protocol, appendices, informed consent form and HIPAA authorization
- Participating Site IRB approved consent form
- Participating Site IRB membership list
- Participating Site IRB's Federal Wide Assurance number and OHRP Registration number
- Curriculum vitae and medical license for each investigator and consenting professional
- Documentation of Human Subject Research Certification training for investigators and key staff members at the Participating Site
- Documentation of Good Clinical Practice (GCP) training for the PI and co-PI at each participating site.
- Participating site laboratory certifications and normals

All applicable regulatory documentation will be needed if patients are treated at the participating sites' affiliate sites. Upon receipt of the required documents, MSKCC will formally contact the site and grant permission to proceed with enrollment.

16.3.1 Amendments

Each change to the protocol document must be organized and documented by MSKCC and first approved by the MSKCC IRB/PB. Upon receipt of MSKCC IRB/PB approval, MSKCC will immediately distribute all non-expedited amendments to the participating sites, for submission to their local IRBs.

Participating sites must obtain approval for all non expedited amendments from their IRB within 90 calendar days of MSKCC IRB/PB approval. If the amendment is the result of a safety issue or makes eligibility criteria more restrictive, sites will not be permitted to continuing enrolling new participants until the participating site IRB approval has been granted.

The following documents must be provided to MSKCC for each amendment within the stated timelines:

- Participating Site IRB approval
- Participating Site IRB approved informed consent form and HIPAA authorization

16.3.2 Additional IRB Correspondence

Continuing Review Approval

The Continuing Review Approval letter from the participating site's IRB and the most current approved version of the informed consent form should be submitted to MSKCC within 7 days of expiration. Failure to submit the re-approval in the stated timeline will result in suspension of study activities.

Deviations

A prospective deviation on this study is defined as a request to treat a research participant who does not meet all the eligibility criteria, pretreatment evaluation, or who requires alteration in their study plan. If a deviation from this protocol is proposed for a potential or existing participant at MSKCC or a participating site, approval from the MSKCC IRB/PB is required prior to the action. Participating sites should contact the MSKCC PI who will in turn seek approval from the MSKCC IRB/PB.

A retrospective deviation is anything that occurs with a participant, which deviated from the protocol without prior approval from the MSKCC IRB/PB. For protocol violations that are identified after they occur, the participating site should report to MSKCC as soon as possible. The MSKCC PI will in turn report the deviaiton to the MSKCC IRB/PB.

Participating sites should report deviations to their institution's IRBs as soon as possible per that site's institutional guidelines. Approvals/acknowledgments from the participating site IRB for protocol deviations should be submitted to MSKCC as received.

Other Correspondence

Participating sites should submit other correspondence to their institution's IRB according to local guidelines, and submit copies of that correspondence to MSKCC.

16.3.3 Document maintenance

The MSKCC PI and the Participating Site PI will maintain adequate and accurate records to enable the implementation of the protocol to be fully documented and the data to be subsequently verified.

The participating sites will ensure that all participating site IRB correspondence (IRB approval letters referencing protocol version date and amendment number, IRB approved protocol, appendices, informed consent forms, deviations, violations, and approval of continuing reviews) is maintained in the regulatory binder on site and sent to MSKCC.

A regulatory binder for each site will also be maintained at MSKCC; this binder may be paper or electronic.

After study closure, the participating site will maintain all source documents, study related documents and CRFs for 3 years

16.4 Noncompliance

If a participating site is noncompliant with the protocol document, accrual privileges may be suspended and/or contract payments may be withheld, until the outstanding issues have been resolved.

17.0 PROTECTION OF HUMAN SUBJECTS

Participation in this trial is voluntary. All patients will be required to sign a statement of informed consent, which must conform to MSKCC IRB guidelines. The informed consent must contain a full explanation of the possible advantages, benefits, risks, alternative treatment options, and availability of treatment in the case of injury, in accordance with Federal Regulations as detailed in 21CFR50. The investigator is responsible for obtaining written informed consent from potential patients before performing any trial tests or assessments required by the protocol. A copy of the signed document will be given to the patient, and the original will be retained by the investigator with his/her copy of the record forms.

Benefits

It is possible that TNT may reduce the risk of distant metastasis and improve survival compared to standard of care. It is not known whether this treatment will affect the overall survival of patients in the study. It is also possible that this treatment may cure some tumors, without the need for surgery. Of course, it is not known whether these or any other favorable events will occur.

Risks to the patient

The main risk to the patient is tumor progression at different times during treatment. Tumor progression, either at the site of the primary tumor or at distant organ(s), can occur while the patient is receiving neoadjuvant treatment. The MSKCC experience and the results of the TIMING trial indicate that tumor progression is uncommon during INCT or CNCT. However, to protect patients against the risk of tumor progression, we will conduct an interim evaluation to monitor for tumor local response and DM during treatment.

It is also possible that tumor relapse during NOM may not be salvageable by TME, and may ultimately compromise survival in patients deemed curable at registration. The experience accumulated so far indicates that, with close surveillance, patients who develop tumor relapse during NOM are successfully salvaged with TME.[41, 43, 62]

Costs

The patient will be responsible for the costs of standard medical care, including all drug administration fees and all hospitalizations, even when these are related to complications of treatment. The patient will not be responsible for the cost of shipping or processing of tissue or blood samples.

Incentives

No incentives will be offered to patients/subjects for participation in the study.

Alternatives

For patients with low LARC, standard therapy including CRT, TME, and ACT may be an option. Patients who decline to participate in the study will be recommended to follow standard care.

Inclusion of Women and Minorities

Patients of all races, both male and female, will be accepted into this protocol. We would expect that at least the same percentage of minority patients will participate in this study as are present in the treating institutions general patient population, and we will actively attempt to accrue minority patients to this protocol.

Exclusion of Children and Lactating or Pregnant Women

Children have been excluded from this study, as there is absolutely no data in existence to judge potential risks in children. This is in keeping with the NCI requirements for this pilot trial. This argument extends to all of our phase II combination trials. The preclinical models tested have been tested in adult cancers (breast, colon, stomach) only. Thus, the relevance of this drug combination to the pediatric population has not been established. Lactating and pregnant women are also excluded because of potential antiproliferative effects of the drug combination, which may be harmful to the developing fetus or nursing infant.

17.1 Privacy

MSKCC's Privacy Office may allow the use and disclosure of protected health information pursuant to a completed and signed Research Authorization form. The use and disclosure of protected health information will be limited to the individuals described in the Research Authorization form. A Research Authorization form must be completed by the Principal Investigator and approved by the IRB and Privacy Board (IRB/PB). The consent indicates that individualized de identified information collected for the purposes of this study may be shared with other qualified researchers. Only researchers who have received approval from MSK will be allowed to access this information which will not include protected health information, such as the participant's name, except for dates. It is also stated in the Research Authorization that their research data may be shared with other qualified researchers.

The consent indicates that samples and genetic information collected may be shared with other qualified researchers and placed in online databases. An example of an online database is the NIH dbGAP database, which is monitored by the National Institutes of Health, and may be made accessible to investigators approved by the U.S. government. Such information will not include identifying information such as name. It is also stated in the Research Authorization that research data (e.g. genomic sequence) may be shared with regulators.
The requirements for submission of genotype/phenotype data into the NIH dbGAP or any other public database will be followed as per the IRB SOP for Genomic Data Sharing.

Confidentiality

Every effort will be made to maintain patient confidentiality. Research and hospital records are confidential. Patient's name or any other personal identifying information will not be used in reports or publications resulting from this study. The Food and Drug Administration or other authorized agencies (e.g., qualified monitors from the treating institution, etc.) may review patients' records and pathology slides, as required.

17.2 Serious Adverse Event (SAE) Reporting

An adverse event is considered serious if it results in ANY of the following outcomes:

- Death
- A life-threatening adverse event
- An adverse event that results in inpatient hospitalization or prolongation of existing hospitalization
- A persistent or significant incapacity or substantial disruption of the ability to conduct normal life functions
- A congenital anomaly/birth defect
- Important Medical Events (IME) that may not result in death, be life threatening, or require hospitalization may be considered serious when, based upon medical judgment, they may jeopardize the patient or participant and may require medical or surgical intervention to prevent one of the outcomes listed in this definition

Note: Hospital admission for a planned procedure/disease treatment is not considered an SAE.

SAE reporting is required as soon as the participant starts investigational treatment/intervention. SAE reporting is required for 30-days after the participant's last investigational treatment/intervention. Any event that occur after the 30-day period that is unexpected and at least possibly related to protocol treatment must be reported.

Please note: Any SAE that occurs prior to the start of investigational treatment/intervention and is related to a screening test or procedure (i.e., a screening biopsy) must be reported.

All SAEs must be submitted in PIMS. If an SAE requires submission to the HRPP office per IRB SOP RR-408 'Reporting of Serious Adverse Events', the SAE report must be submitted within 5 calendar days of the event. All other SAEs must be submitted within 30 calendar days of the event.

The report should contain the following information:

- The date the adverse event occurred
- The adverse event
- The grade of the event
- Relationship of the adverse event to the treatment(s)
- If the AE was expected
- Detailed text that includes the following
  - An explanation of how the AE was handled
  - A description of the participant's condition
  - Indication if the participant remains on the study
- If an amendment will need to be made to the protocol and/or consent form

- If the SAE is an Unanticipated Problem

For IND/IDE protocols: The SAE report should be completed as per above instructions. If appropriate, the report will be forwarded to the FDA by the IND Office

18.0 INFORMED CONSENT PROCEDURES

Before protocol-specified procedures are carried out, consenting professionals will explain full details of the protocol and study procedures as well as the risks involved to participants prior to their inclusion in the study. Participants will also be informed that they are free to withdraw from the study at any time. All participants must sign an IRB/PB-approved consent form indicating their consent to participate. This consent form meets the requirements of the Code of Federal Regulations and the Institutional Review Board/Privacy Board of this Center. The consent form will include the following:

1. The nature and objectives, potential risks and benefits of the intended study.
2. The length of study and the likely follow-up required.
3. Alternatives to the proposed study. (This will include available standard and investigational therapies. In addition, patients will be offered an option of supportive care for therapeutic studies.)
4. The name of the investigator(s) responsible for the protocol.
5. The right of the participant to accept or refuse study interventions/interactions and to withdraw from participation at any time.

Before any protocol-specific procedures can be carried out, the consenting professional will fully explain the aspects of patient privacy concerning research specific information. In addition to signing the IRB Informed Consent, all patients must agree to the Research Authorization component of the informed consent form.

Each participant and consenting professional will sign the consent form. The participant must receive a copy of the signed informed consent form.

18.1 For participating sites

The investigators listed on the Consenting Professionals List at each participating site may obtain informed consent and care for the participants according to good clinical practice and protocol guidelines.

A note will be placed in the medical record documenting that informed consent was obtained for this study, and that the participant acknowledges the risks of participation.

19.0 REFERENCES

1. Sauer, R., et al., *Preoperative versus postoperative chemoradiotherapy for rectal cancer.* N Engl J Med, 2004. **351**(17): p. 1731-40.

2. Minsky, B.D., C. Roedel, and V. Valentini, *Combined modality therapy for rectal cancer.* Cancer J, 2010. **16**(3): p. 253-61.
3. Pucciarelli, S., Gagliardi, G., Maretto, I., Lonardi, S., Friso, M.L., Urso, E., Toppan, P., Nitti, D., *Long-term oncologic results and complications after preoperative chemoradiotherapy for rectal cancer: a single-institution experience after a median follow-up of 95 months.* Ann Surg Oncol, 2009. **16**(4): p. 893-899.
4. Engstrom, P.F., et al., *NCCN Clinical Practice Guidelines in Oncology: colon cancer.* J Natl Compr Canc Netw, 2009. **7**(8): p. 778-831.
5. Petersen, S.H., et al., *Postoperative adjuvant chemotherapy in rectal cancer operated for cure.* Cochrane Database Syst Rev, 2012. **3**: p. CD004078.
6. Bosset, J.F., et al., *Chemotherapy with preoperative radiotherapy in rectal cancer.* N Engl J Med, 2006. **355**(11): p. 1114-23.
7. Aschele, C., et al., *Primary tumor response to preoperative chemoradiation with or without oxaliplatin in locally advanced rectal cancer: pathologic results of the STAR-01 randomized phase III trial.* J Clin Oncol, 2011. **29**(20): p. 2773-80.
8. Gerard, J.P., et al., *Comparison of two neoadjuvant chemoradiotherapy regimens for locally advanced rectal cancer: results of the phase III trial ACCORD 12/0405-Prodige 2.* J Clin Oncol, 2010. **28**(10): p. 1638-44.
9. Roh MS, Y.G., O'Connell MJ, Beart RW, Pitot HC, Shields AF, et al, *The impact of capecitabine and oxaliplatin in the preoperative multimodality treatment in patients with carcinoma of the rectum: NSABP R-04.* J Clin Oncol, 2011. **29**(Suppl 15, Abstract No. 3503): p. 221s.
10. Fernandez-Martos, C., et al., *Phase II, randomized study of concomitant chemoradiotherapy followed by surgery and adjuvant capecitabine plus oxaliplatin (CAPOX) compared with induction CAPOX followed by concomitant chemoradiotherapy and surgery in magnetic resonance imaging-defined, locally advanced rectal cancer: Grupo cancer de recto 3 study.* J Clin Oncol, 2010. **28**(5): p. 859-65.
11. Rodel, C., et al., *Preoperative chemoradiotherapy and postoperative chemotherapy with fluorouracil and oxaliplatin versus fluorouracil alone in locally advanced rectal cancer: initial results of the German CAO/ARO/AIO-04 randomised phase 3 trial.* Lancet Oncol, 2012. **13**(7): p. 679-87.
12. Dewdney, A., et al., *Multicenter Randomized Phase II Clinical Trial Comparing Neoadjuvant Oxaliplatin, Capecitabine, and Preoperative Radiotherapy With or Without Cetuximab Followed by Total Mesorectal Excision in Patients With High-Risk Rectal Cancer (EXPERT-C).* J Clin Oncol, 2012. **30**(14): p. 1620-7.
13. Glynne-Jones, R., Grainger J, Harrison M, Ostler P, Makris A, *Neoadjuvant chemotherapy prior to preoperative chemoradiation or radiation in rectal cancer: should we be more cautious?* Br J Cancer, 2006. **94**(3): p. 363-371.
14. Chau I, B.G., Cunningham D, Tait D, Wotherspoon A, Norman AR, Tebbutt N, Hill M, Ross PJ, Massey A, Oates J, *Neoadjuvant capecitabine and oxaliplatin followed by synchronous chemoradiation and total mesorectal excision in magnetic resonance imagine-defined poor-risk rectal cancer.* J Clin Oncol, 2006. **24**(4): p. 668-674.
15. Calvo FA, S.F., Diaz-Gonzalez JA, Gomez Espi M, Lozano E, Garcia R, de la Mata D, Arranz JA, Garcia-Alfonso P, Perez-Manga G, Alvarez E, *Improved incidence of pT0 downstaged surgical specimens in locally advanced rectal cancer (LARC) treated with induction oxaliplatin plus 5-fluorouracil and preoperative chemoradiation.* Ann Oncol, 2006. **17**(7): p. 1103-1110.
16. Koeberle, D., et al., *Phase II study of capecitabine and oxaliplatin given prior to and concurrently with preoperative pelvic radiotherapy in patients with locally advanced rectal cancer.* Br J Cancer, 2008. **98**(7): p. 1204-9.
17. Chua, Y.J., et al., *Neoadjuvant capecitabine and oxaliplatin before chemoradiotherapy and total mesorectal excision in MRI-defined poor-risk rectal cancer: a phase 2 trial.* Lancet Oncol, 2010. **11**(3): p. 241-8.

18. Schou JV, L.F., Rasch L, Linnemann D, Langhoff J, Hogdall E, Nielsen DL, Vistisen K, Fromm A, Jensen BV, *Induction chemotherapy with capecitabine and oxaliplatin followed by chemoradiotherapy before total mesorectal excision in patients with locally advanced rectal cancer.* Ann Oncol, 2012. **23**(10): p. 2627-2633.
19. Marechal R, V.B., Polus M, Delaunoit T, Peeters M, Demetter P, Hendisz A, Demols A, Franchimont D, Verset G, Van Houtte P, Van de Stadt J, Van Laethem JL, *Short course chemotherapy followed by concomitant chemoradiotherapy and surgery in locally advanced rectal cancer: a randomized multicentric phase II study.* Ann Oncol, 2011. **23**(6): p. 1525-1530.
20. Schrag D, W.M., Goodman KA, Gonen M, Cercek A, Reidy DL, et al, *Neoadjuvant FOLFOX-bev, without radiation, for locally advanced rectal cancer.* J Clin Oncol, 2010. **28**(Suppl): p. 15S.
21. Hajj C WM, M.A., Gollub M, Cercek A, Ding PR, Saltz L, Goodman KA, *Induction chemotherapy followed by chemoradiotherapy and TME for high-risk locally advanced rectal cancer with and without synchronous resectable metastases.* J Clin Oncol, 2012. **30**(Suppl 4): p. 599.
22. Garcia-Aguilar, J., et al., *Identification of a biomarker profile associated with resistance to neoadjuvant chemoradiation therapy in rectal cancer.* Ann Surg, 2011. **254**(3): p. 486-92; discussion 492-3.
23. Habr-Gama, A., et al., *Increasing the rates of complete response to neoadjuvant chemoradiotherapy for distal rectal cancer: results of a prospective study using additional chemotherapy during the resting period.* Dis Colon Rectum, 2009. **52**(12): p. 1927-34.
24. Peeters, K.C., et al., *Late side effects of short-course preoperative radiotherapy combined with total mesorectal excision for rectal cancer: increased bowel dysfunction in irradiated patients--a Dutch colorectal cancer group study.* J Clin Oncol, 2005. **23**(25): p. 6199-206.
25. Hendren, S.K., et al., *Prevalence of male and female sexual dysfunction is high following surgery for rectal cancer.* Ann Surg, 2005. **242**(2): p. 212-23.
26. Wiatrek, R.L., J.S. Thomas, and H.T. Papaconstantinou, *Perineal wound complications after abdominoperineal resection.* Clin Colon Rectal Surg, 2008. **21**(1): p. 76-85.
27. Robertson, I., et al., *Prospective analysis of stoma-related complications.* Colorectal Dis, 2005. **7**(3): p. 279-85.
28. Formijne Jonkers, H.A., et al., *Early complications after stoma formation: a prospective cohort study in 100 patients with 1-year follow-up.* Int J Colorectal Dis, 2012. **27**(8): p. 1095-9.
29. Bhangu, A., D. Nepogodiev, and K. Futaba, *Systematic review and meta-analysis of the incidence of incisional hernia at the site of stoma closure.* World J Surg, 2012. **36**(5): p. 973-83.
30. Zotti P, D.B.P., Serpentini S, Trevisanut P, Barba MC, Valentini V, De Paoli A, Pucciarelli S., *Validity and reliability of the MSKCC Bowel Function instrument in a sample of Italian rectal cancer patients.* Eur J Surg Oncol, 2011. **37**(7): p. 589-596.
31. Pollack J, H.T., Cedermark B, Altman D, Holmström B, Glimelius B, Mellgren A., *Late adverse effects of short-course preoperative radiotherapy in rectal cancer.* Br J Surg, 2006. **93**(12): p. 1519-1525.
32. Hoerske C, W.K., Goehl J, Hohenberger W, Merkel S., *Long-term outcomes and quality of life after rectal carcinoma surgery.* Br J Surg, 2010. **97**(8): p. 1295-1303.
33. Cornish JA, T.H., Heriot AG, Lavery IC, Fazio VW, Tekkis PP., *A meta-analysis of quality of life for abdominoperineal excision of rectum versus anterior resection for rectal cancer.* Ann Surg Oncol, 2007. **14**(7): p. 2056-68.
34. Kim, J.Y., et al., *A comparative study of voiding and sexual function after total mesorectal excision with autonomic nerve preservation for rectal cancer: laparoscopic versus robotic surgery.* Ann Surg Oncol, 2012. **19**(8): p. 2485-93.

35. Maas, M., et al., *Long-term outcome in patients with a pathological complete response after chemoradiation for rectal cancer: a pooled analysis of individual patient data.* Lancet Oncol, 2010. **11**(9): p. 835-44.
36. Glynne-Jones, R., Hughes R, *Critical appraisal of the "wait and see" approach in rectal cancer for clinical complete responders after chemoradiation.* Br J Surg, 2012. **99**: p. 897-909.
37. Dedemadi, G. and S.D. Wexner, *Complete response after neoadjuvant therapy in rectal cancer: to operate or not to operate?* Dig Dis, 2012. **30 Suppl 2**: p. 109-17.
38. Tepper, J.E. and B.H. O'Neil, *Minimizing therapy and maximizing outcomes in rectal cancer.* J Clin Oncol, 2011. **29**(35): p. 4604-6.
39. Kusters, M., et al., *Patterns of local recurrence in locally advanced rectal cancer after intra-operative radiotherapy containing multimodality treatment.* Radiother Oncol, 2009. **92**(2): p. 221-5.
40. Burt, C.G., et al., *Developing a research agenda for the American Society of Colon and Rectal Surgeons: results of a delphi approach.* Dis Colon Rectum, 2009. **52**(5): p. 898-905.
41. Habr-Gama, A., et al., *Operative versus nonoperative treatment for stage 0 distal rectal cancer following chemoradiation therapy: long-term results.* Ann Surg, 2004. **240**(4): p. 711-7; discussion 717-8.
42. Lambregts DMJ, V.V., Barbaro B, Bakers FCH, Lambrecht M, Maas M, Haustermans K, Valentini V, Beets GL, Beets-Tan RGH, *Diffusion-weighted MRI for selection of completion responders after chemoradiation for locally advanced rectal cancer: a multicenter study.* Ann Surg Oncol, 2011. **18**: p. 2224-2231.
43. Smith JD, R.J., Goodman K, Saltz L, Guillem JG, Weiser MR, et al., *Non-operative management of rectal cancer with complete clinical response following neoadjuvant therapy.* 2012.
44. Temple, L.K., et al., *The development of a validated instrument to evaluate bowel function after sphincter-preserving surgery for rectal cancer.* Dis Colon Rectum, 2005. **48**(7): p. 1353-65.
45. Weiser MR, G.J., Nash G, Paty PB, Temple L, Patil S, Yeo HL, *Understanding bowel dysfunction after sphincter preserving surgery: results from a prospective study*, in *Society of Surgical Oncology 64th Annual Cancer Symposium*. 2011: San Antonio, Texas.
46. Masya, L.M., et al., *Preferences for outcomes of treatment for rectal cancer: patient and clinician utilities and their application in an interactive computer-based decision aid.* Dis Colon Rectum, 2009. **52**(12): p. 1994-2002.
47. Kennedy, E.D., et al., *Do patients consider preoperative chemoradiation for primary rectal cancer worthwhile?* Cancer, 2011. **117**(13): p. 2853-62.
48. Guillem JG, C.D., Shia J, Moore HG, Mazumdar M, Bernard B, Paty PB, Saltz L, Minsky BD, Weiser MR, Temple LKF, Cohen AM, Wong WD, *Clinical examination following preoperative chemoradiation for rectal cancer is not a reliable surrogate end point.* J Clin Oncol, 2005. **23**(15): p. 3475-3479.
49. Pastor C, S.J., Sola J, Baixauli J, Beorlegui C, Arbea L, Aristu J, Hernandez-Lizoain JL., *Accuracy of endoscopic ultrasound to assess tumor response after neoadjuvant treatment in rectal cancer: can we trust the findings?*
Dis Colon Rectum, 2011. **54**(9): p. 1141-6.
50. Patel UB, T.F., Blomqvist L, George C, Evans H, Tekkis P, Quirke P, Sebag-Montefiore D, Moran B, Heald R, Guthrie A, Bees N, Swift I, Pennert K, Brown G, *Magnetic resonance imaging-detected tumor response for locally advanced rectal cancer predicts survival outcomes: MERCURY experience.* J Clin Oncol, 2011. **29**(28): p. 3753-3760.
51. Gollub MJ, G.D., Akin O, Do RK, Fuqua 3rd JL, Gonen M, Kuk D, Weiser M, Saltz L, Schrag D, Goodman K, Paty P, Guillem J, Nash GM, Temple L, Shia J, Schwartz LH, *Dynamic contrast enhanced-MRI for the detection of pathological complete response to neoadjuvant chemotherapy for locally advanced rectal cancer.* Eur Radiol, 2012. **22**: p. 821-831.

52. Al-Sukhni E, M.D., Charles Victor J, McLeod RS, Kennedy ED., *Do MRI Reports Contain Adequate Preoperative Staging Information for End Users to Make Appropriate Treatment Decisions for Rectal Cancer?* Ann Surg Oncol, 2012. **[Epub ahead of print]**.
53. Dresen RC, B.G., Rutten HJT, Engelen SME, Lahaye MJ, Vliegen RFA, de Bruine AP, Kessels AGH, Lammering G, Beets-Tan RGH, *Locally advanced rectal cancer: MR imaging for restaging after neoadjuvant radiaiton therapy with concomitant chemotherapy Part I. Are we able to predict tumor confined to the rectal wall?* Radiology, 2009. **252**(1): p. 71-80.
54. Nougaret S, R.P., Molinari N, Pierredon MA, Bibeau F, Azria D, Lemanski C, Assenat E, Duffour J, Ychou M, Reinhold C, Gallix B, *MR volumetric measurement of low rectal cancer helps predict tumor response and outcome after combined chemotherapy and radiation therapy.* Radiology, 2012. **263**(2): p. 409-418.
55. Hejjmen L, V.M., ter Voert EEGW, Punt CJA, Oyen WJG, de Geus-Oei LF, Hermans JJ, Heerschap A, van Laarhoven HWM, *Tumour response prediction by diffusion-weighted MR imaging: ready for clinical use?* Crit Rev Oncol Hematol, 2012. **83**: p. 194-207.
56. Dzik-Jurasz, A., Domenig C, George M, Wolber J, Padhani A, Brown G, Doran S, *Diffusion MRI for prediction of response of rectal cancer to chemoradiation.* Lancet, 2002. **360**: p. 307-308.
57. Kim SH, L.J., Hong SH, Kim GH, Lee JY, Han JK, Choi BI, *Locally advanced rectal cancer: added value of diffusion-weighted MR imaging in the evaluation of tumor response to neoadjuvant chemo- and radiation therapy.* Radiology, 2009. **253**(1): p. 116-125.
58. Kim YC, L.J., Keum KC, Kim KA, Myoung S, Shin SJ, Kim MJ, Kim NK, Suh J, Kim KW, *Comparison of diffusion-weighted MRI and MR volumetry in the evaluation of early treatment outcomes after preoperative chemoradiotherapy for locally advanced rectal cancer* J Magn Reson Imaging, 2011. **34**: p. 570-576.
59. Barbaro B, V.R., Valentini V, Illuminati S, Vecchio FM, Rizzo G, Gambacorta MA, Coco C, Crucitti A, Persiani R, Sofo L, Bonomo L, *Diffusion-weighted magnetic resonance imaging in monitoring rectal cancer response to neoadjuvant chemoradiotherapy.* Int J Radiat Oncol Biol Phys, 2012. **83**(2): p. 594-599.
60. Kim, S.H., et al., *Apparent diffusion coefficient for evaluating tumour response to neoadjuvant chemoradiation therapy for locally advanced rectal cancer.* Eur Radiol, 2011. **21**(5): p. 987-95.
61. Lambrecht M, V.V., De Keyzer F, Roels S, Penninckx F, Van Cutsem E, Filip C, Haustermans K, *Value of diffusion-weighted magnetic resonance imaging for prediction and early assessment of response to neoadjuvant radiochemotherapy in rectal cancer: preliminary results.* Int J Radiat Oncol Biol Phys, 2012. **82**(2): p. 863-870.
62. Curvo-Semedo L, L.D., Maas M, Thywissen T, Mehsen RT, Lammering G, Beets GL, Caseiro-Alves F, Beets-Tan RGH, *Rectal cancer: Assessment of complete response to preoperative combined radiation therapy with chemotherapy--conventional MR volumtry versus diffusion-weighted MR imaging.* Radiology, 2011. **260**(3): p. 734-743.
63. Lecomte, T., et al., *Detection of free-circulating tumor-associated DNA in plasma of colorectal cancer patients and its association with prognosis.* Int J Cancer, 2002. **100**(5): p. 542-8.
64. Yen, L.C., et al., *Detection of KRAS oncogene in peripheral blood as a predictor of the response to cetuximab plus chemotherapy in patients with metastatic colorectal cancer.* Clin Cancer Res, 2009. **15**(13): p. 4508-13.
65. Diehl, F., et al., *Circulating mutant DNA to assess tumor dynamics.* Nat Med, 2008. **14**(9): p. 985-90.
66. Kloosterman, W.P. and R.H. Plasterk, *The diverse functions of microRNAs in animal development and disease.* Dev Cell, 2006. **11**(4): p. 441-50.
67. Deng, S., et al., *Mechanisms of microRNA deregulation in human cancer.* Cell Cycle, 2008. **7**(17): p. 2643-6.
68. Di Leva, G. and C.M. Croce, *Roles of small RNAs in tumor formation.* Trends Mol Med, 2010. **16**(6): p. 257-67.

69. Dykxhoorn, D.M., *MicroRNAs and metastasis: little RNAs go a long way.* Cancer Res, 2010. **70**(16): p. 6401-6.
70. Iorio, M.V. and C.M. Croce, *MicroRNAs in cancer: small molecules with a huge impact.* J Clin Oncol, 2009. **27**(34): p. 5848-56.
71. Kumar, M.S., et al., *Impaired microRNA processing enhances cellular transformation and tumorigenesis.* Nat Genet, 2007. **39**(5): p. 673-7.
72. Spizzo, R., et al., *SnapShot: MicroRNAs in Cancer.* Cell, 2009. **137**(3): p. 586-586 e1.
73. Lu, J., et al., *MicroRNA expression profiles classify human cancers.* Nature, 2005. **435**(7043): p. 834-8.
74. Ng, E.K., et al., *Differential expression of microRNAs in plasma of patients with colorectal cancer: a potential marker for colorectal cancer screening.* Gut, 2009. **58**(10): p. 1375-81.
75. Cummins, J.M., et al., *The colorectal microRNAome.* Proc Natl Acad Sci U S A, 2006. **103**(10): p. 3687-92.
76. Schetter, A.J., et al., *MicroRNA expression profiles associated with prognosis and therapeutic outcome in colon adenocarcinoma.* JAMA, 2008. **299**(4): p. 425-36.
77. Garcia-Aguilar, J., et al., *Optimal timing of surgery after chemoradiation for advanced rectal cancer: preliminary results of a multicenter, nonrandomized phase II prospective trial.* Ann Surg, 2011. **254**(1): p. 97-102.
78. Spindler, K.L., et al., *Germline polymorphisms may act as predictors of response to preoperative chemoradiation in locally advanced T3 rectal tumors.* Dis Colon Rectum, 2007. **50**(9): p. 1363-9.
79. Chen, Z., et al., *Chromosomal copy number alterations are associated with persistent lymph node metastasis after chemoradiation in locally advanced rectal cancer.* Dis Colon Rectum, 2012. **55**(6): p. 677-85.
80. Chen, Z., et al., *Chromosomal copy number alterations are associated with tumor response to chemoradiation in locally advanced rectal cancer.* Genes Chromosomes Cancer, 2011. **50**(9): p. 689-99.
81. Brettingham-Moore, K.H., et al., *Pretreatment transcriptional profiling for predicting response to neoadjuvant chemoradiotherapy in rectal adenocarcinoma.* Clin Cancer Res, 2011. **17**(9): p. 3039-47.
82. Rimkus, C., et al., *Microarray-based prediction of tumor response to neoadjuvant radiochemotherapy of patients with locally advanced rectal cancer.* Clin Gastroenterol Hepatol, 2008. **6**(1): p. 53-61.
83. Cheng, L., et al., *The clinical and therapeutic implications of cancer stem cell biology.* Expert Rev Anticancer Ther, 2011. **11**(7): p. 1131-43.
84. Yu, Y., G. Ramena, and R.C. Elble, *The role of cancer stem cells in relapse of solid tumors.* Front Biosci (Elite Ed), 2012. **4**: p. 1528-41.
85. Walker, F., et al., *LGR5 is a negative regulator of tumourigenicity, antagonizes Wnt signalling and regulates cell adhesion in colorectal cancer cell lines.* PLoS One, 2011. **6**(7): p. e22733.
86. Buczacki, S., R.J. Davies, and D.J. Winton, *Stem cells, quiescence and rectal carcinoma: an unexplored relationship and potential therapeutic target.* Br J Cancer, 2011. **105**(9): p. 1253-9.
87. *AJCC Cancer Staging Manual*. 7th ed, ed. E.S.B.D.C.C.F.A.G.F.T. A. 2009, New York: Springer-Verlag.
88. Dindo, D., N. Demartines, and P.A. Clavien, *Classification of surgical complications: a new proposal with evaluation in a cohort of 6336 patients and results of a survey.* Ann Surg, 2004. **240**(2): p. 205-13.
89. Garcia-Aguilar J, S.Q., Thomas CR Jr, Chan E, Cataldo P, Marcet J, Medich D, Pigazzi A, Oommen S, Posner MC, *A phase II trial of neoadjuvant chemoradiation and local excision for T2N0 rectal cancer: preliminary results of the ACOSOG Z6041 trial.* Ann Surg Oncol, 2012. **19**(2): p. 384-391.
90. Baxter, N.N., et al., *A stoma quality of life scale.* Dis Colon Rectum, 2006. **49**(2): p. 205-12.

91. Rosen, R.C., et al., *The international index of erectile function (IIEF): a multidimensional scale for assessment of erectile dysfunction.* Urology, 1997. **49**(6): p. 822-30.
92. Rosen RC, C.J., Smith MD, Lisky J, Pena BM, *Development and evaluation of an abridged, 5-item version of the International Index of Erectile Function (IIEF-5) as a diagnostic tool for erectile dysfunction.* Int J Impotence Res, 1999. **11**: p. 319-326.
93. Rosen, R., et al., *The Female Sexual Function Index (FSFI): a multidimensional self-report instrument for the assessment of female sexual function.* J Sex Marital Ther, 2000. **26**(2): p. 191-208.
94. Herdman, M., et al., *Development and preliminary testing of the new five-level version of EQ-5D (EQ-5D-5L).* Qual Life Res, 2011. **20**(10): p. 1727-36.
95. JF, L., *Statistical Models and Methods for Lifetime Data*. 1982, New York: John Wiley & Sons, Inc.
96. Jennison C, T.B., *Group Sequential Meethods with Applications to Clinical Trials*. 1 ed. 1999: Chapman and Hall/CRC Interdisciplinary Statistics.
97. Emmertsen KJ, Laurberg S. Low anterior resection syndrome score: development and validation of a symptom-based scoring system for bowel dysfunction after low anterior resection for rectal cancer. *Annals of surgery.* 2012;255(5):922-928.
98. Emmertsen KJ, Laurberg S, Rectal Cancer Function Study G, et al. Impact of bowel dysfunction on quality of life after sphincter-preserving resection for rectal cancer. *The British journal of surgery.* 2013;100(10):1377-1387.

20.0 APPENDICES

Appendix A: Stoma Male Survey

Appendix B: Stoma Female Survey

Appendix C: No Stoma Male Survey

Appendix D: No Stoma Female Survey

Appendix E: Stoma Male and Female Survey (MSKCC patients only)

Appendix F: MRI Technical and Quality Requirements

Appendix G: Capecitabine Pill Diary during Chemotherapy

Appendix H: Capecitabine Pill Diary during Radiation Therapy

Appendix I: Radiation Therapy QA Sheet (document no longer in use)

Appendix J: Chemotherapy QA Sheet (document no longer in use)

Appendix K: Surgeon Response Criteria (document no longer in use)

Appendix L: Radiologist Response Criteria (document no longer in use)

Appendix M: MSKCC Lab Requisition Form

Appendix N: Clavien-Dindo Classification of Surgical Complications

Appendix O: MSKCC Regional Sites Lab Requisition Form

Appendix P: External Site SAE Form
