# Supplementary material for: Organ Preservation and Survival by Clinical Response Grade in Patients With Rectal Cancer Treated With Total Neoadjuvant Therapy: A Secondary Analysis of the OPRA Randomized Clinical Trial
Source: JAMA Netw Open. 2024 Jan 9;7(1):e2350903. doi: 10.1001/jamanetworkopen.2023.50903 (PMC10777257; doi:10.1001/jamanetworkopen.2023.50903)
Supplement: Supplement 2. — eFigure 1. Three-Tier Schema for Grading Tumor Response eTable. Local and Distant Metastases for TME at Restaging Versus TME After Local Regrowth in Relation to Clinical Tumor Response eFigure 2. Rates of Organ Preservation and Disease-Free Survival for the Full Cohort eFigure 3. Rates of Local Regrowth [file jamanetwopen-e2350903-s002.pdf]

Supplemental Online Content

Thompson HM, Omer DM, Lin S, et al; on behalf of the OPRA Consortium. Organ preservation and survival by clinical response grade in patients with rectal cancer treated with total neoadjuvant therapy. *JAMA Netw Open*. 2024;7(1):e2350903. doi:10.1001/jamanetworkopen.2023.50903

eFigure 1. Three-Tier Schema for Grading Tumor Reponse
eTable. Local and Distant Metastases for TME at Restaging Versus TME After Local Regrowth in Relation to Clinical Tumor Reponse
eFigure 2. Rates of Organ Preservation and Disease-Free Survival for the Full Cohort
eFigure 3. Rates of Local Regrowth

This supplemental material has been provided by the authors to give readers additional information about their work.

© 2023 Thompson HM et al. *JAMA Network Open.*

eFigure 1. Three-Tier Schema for Grading Tumor Response

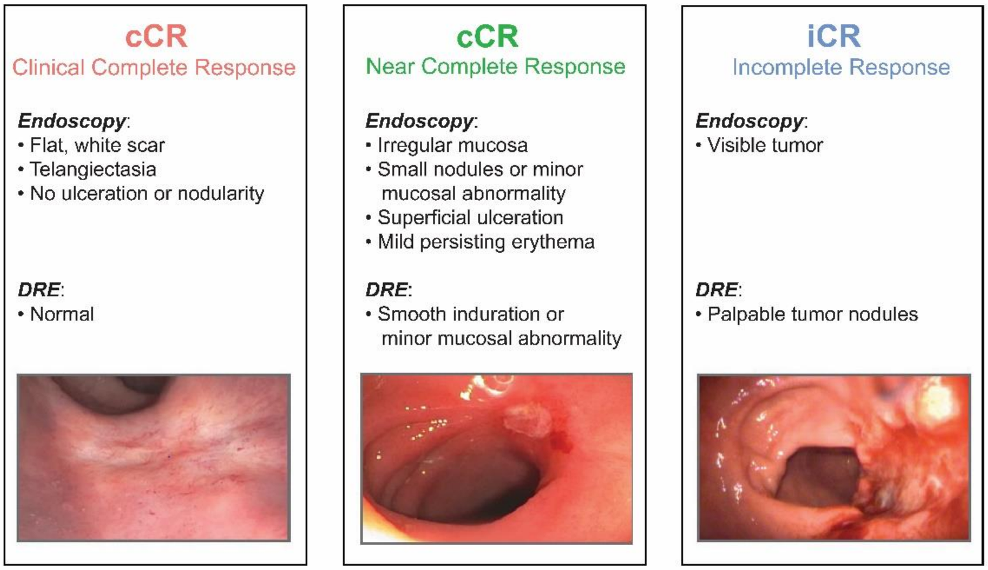

eTable. Local and Distant Metastases for TME at Restaging Versus TME After Local Regrowth in Relation to Clinical Tumor Response

	TME Recommended at Restaging (n = 79)			TME Recommended after Local Regrowth (n = 80)		
	cCR (n = 2)*	nCR (n = 20)	iCR (n = 57)†	cCR (n = 27)	nCR (n = 48)**	iCR (n = 5)
Local recurrence (n = 8)	0	3 (15%)	2 (3.5%)	1 (3.7%)	2 (4.2%)	1 (20%)
Distant recurrence (n = 30)	0	2 (10%)	12 (21.1%)	6 (22.2%)	10 (20.8%)	0
Both distant and local recurrence (n = 13)	0	1 (5%)	6 (10.5%)	2 (7.4%)	4 (8.3%)	0

*One patient refused. They are counted as persistent disease.
**Eight refused (1 of them also had distant recurrence). They are counted as persistent disease.
†Three patients refused. They are counted as persistent disease.

© 2023 Thompson HM et al. *JAMA Network Open.*

eFigure 2. Rates of Organ Preservation and Disease-Free Survival

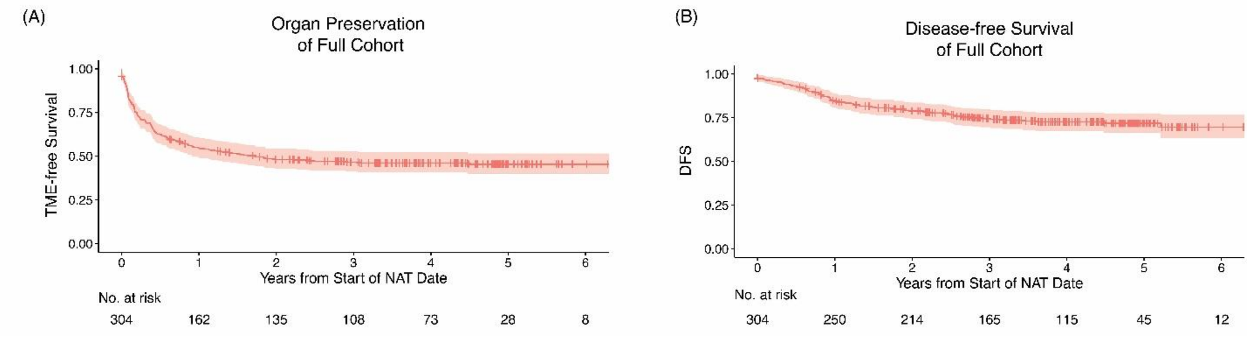

.

eFigure 3. Rates of Local Regrowth

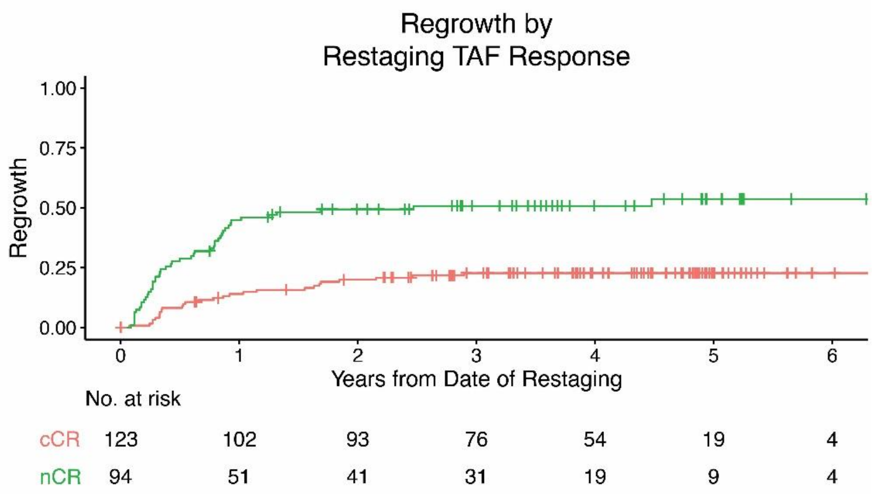

© 2023 Thompson HM et al. *JAMA Network Open.*
