# Supplementary material for: Organ Preservation and Survival by Clinical Response Grade in Patients With Rectal Cancer Treated With Total Neoadjuvant Therapy: A Secondary Analysis of the OPRA Randomized Clinical Trial
Source: JAMA Netw Open. 2024 Jan 9;7(1):e2350903. doi: 10.1001/jamanetworkopen.2023.50903 (PMC10777257; doi:10.1001/jamanetworkopen.2023.50903)
Supplement: Supplement 3. — Data Sharing Statement [file jamanetwopen-e2350903-s003.pdf]

Data Sharing Statement

Thompson. Organ Preservation and Survival by Clinical Response Grade in Patients With Rectal Cancer Treated with Total Neoadjuvant Therapy. *JAMA Netw Open*. Published January 09, 2024. doi:10.1001/jamanetworkopen.2023.50903

Data
Data available: Yes
Data types: Deidentified participant data
How to access data: garciaaj@mskcc.org
When available: With publication

Supporting Documents
Document types: Informed consent form
How to access documents: garciaaj@mskcc.org
When available: With publication

Additional Information
Who can access the data: Researchers whose proposed use of the data has been approved.
Types of analyses: For a specified purpose.
Mechanisms of data availability: With a signed data access agreement.
